# Qili Qiangxin capsule attenuates myocardial fibrosis by modulating collagen homeostasis post-infarction in rats

**DOI:** 10.1371/journal.pone.0310897

**Published:** 2024-09-27

**Authors:** Minyan Sun, Chunhua Liu, Kehan Gao, Xingming Xu, Kunhan Chen, Liang Qiu, Xiaomin Wang

**Affiliations:** 1 Top Discipline of Jiangxi Province, Discipline of Chinese and Western Integrative Medicine, Jiangxi University of Chinese Medicine, Nanchang, Jiangxi, China; 2 Key Laboratory of Chinese Medicine for Prevention and Treatment of Vascular Remodeling Related Diseases, Jiangxi University of Chinese Medicine, Nanchang, Jiangxi, China; Georgia State University, UNITED STATES OF AMERICA

## Abstract

Myocardial fibrosis (MF) is a major cause of morbidity and mortality worldwide. Qili Qiangxin capsule (QLQX) is a traditional Chinese medicine (TCM) formula used for treating MF, QLQX can affect ventricular remodeling by regulating collagen deposition; however, the specific mechanism by which QLQX modulates collagen homeostasis remains unclear. Thus, this study aimed to explore the effect of QLQX on collagen fibers and its mechanism of action in rats after myocardial infarction (MI). Rats were subjected to left anterior descending artery ligation and then were divided equally into five groups: sham, model, low-dose QLQX, high-dose QLQX and empagliflozin groups. QLQX treatment for 28 days significantly improved cardiac function, as evidenced by decreases in heart mass index, cardiac volume, left ventricular end-diastolic diameter, left ventricular end-systolic diameter, N-terminal B-type natriuretic peptide levels, and high-sensitivity cardiac troponin I levels and increases in left ventricular ejection fraction and left ventricular fraction shortening. Hematoxylin and eosin, Masson, and Picrosirius red staining under a light microscope indicated that QLQX treatment suppressed fibrosis and promoted angiogenesis by decreasing the protein expression levels of proteins related to cardiac remodeling including transforming growth factor-β1, metalloproteinase-9 and α-smooth muscle actin and increasing the expression of tissue inhibitor of matrix metalloproteinase-1 concentration. Picrosirius red staining under the polarized light microscope and western blotting showed that MI increased the contents of collagen I and III, and reduced the contents of collagen II and IV. QLQX treatment improved cardiac function and attenuated MF by modulating collagen homeostasis and promoting angiogenesis. This study provides novel insights into the mechanism of action of QLQX in preventing MF after MI.

## Introduction

Cardiovascular disease is a global health issue that is responsible for approximately 18 million deaths every year [1]. Myocardial infarction (MI) is a major cause of morbidity and mortality worldwide [2]. Primary percutaneous coronary intervention is the main clinical treatment for MI to restore coronary blood supply [3]. However, despite receiving this intervention, a significant proportion of patients with MI (15%–25%) develop chronic congestive heart failure (HF). These patients have a high in-hospital mortality rate of up to 40%, with the recurrence rate of HF reaching 23.4% within a year among those who survive MI [4]. Currently recommended pharmacologic treatments for HF include angiotensin-converting enzyme inhibition, angiotensin type I receptor blocker therapy, and β-adrenergic receptor blocker therapy. However, these strategies have limited effectiveness in preventing MI. Myocardial fibrosis (MF) is the excessive accumulation of extracellular matrix (ECM) and fibroblast deposition in the myocardium [5]. Weisman et al. suggested that MF is closely related to HF after MI and is inevitable in HF development [6]. Consequently, novel strategies to prevent MF, reverse ventricular remodeling, minimize patient mortality and effective interventions to minimize infarct extension and improve cardiac function are urgently needed.

Qili Qiangxin capsule (QLQX or QL), a traditional Chinese medicine (TCM) formula approved by the China Food and Drug Administration [7], has been proven safe and effective for the treatment of chronic HF. QLQX enhances myocardial contractility and diuresis; inhibits over-activation of neuroendocrine systems such as the renin-angiotensin-aldosterone system (RAAS); inhibits inflammation, MF, apoptosis, and autophagy; improves myocardial energy metabolism; promotes angiogenesis and enhances endothelial function [8]. *According to the drug instructions and the Chinese Pharmacopoeia*, it consists of eleven Chinese herbs: *Astragalus membranaceus (Fisch*.*) Bge*. *var*. *mongholicus (Bge*.*) Hsiao*, *Panax ginseng C*. *A*. *Mey*, *Salvia miltiorrhiza Bunge*, *Alisma orientale (Sam*.*) Juzep*, *Rhizoma Atractylodis Macrocephalae*, *Lepidium apetalum Willd*, *Aconitum carmichaelii Debx*, *Cinnamomum cassia (L*.*) J*. *Presl*, *Carthamus tinctorius L*, *Citrus reticulata Blanco and Rhizoma Polygonati* as shown in Table 1. A previous study has shown that QLQX can improve cardiac function, and attenuate MF and ventricular remodeling in doxorubicin-induced HF rats by promoting transforming growth factor (TGF)-β3 / Smad7 and inhibiting TGF-β1 / Smad3 [9]. It also attenuated cardiac remodeling in rats with MI by inhibiting TGF-β1 / Smad3 and NF-κB signaling pathways [10]. A randomized controlled study demonstrated that QLQX in combination with conventional therapy can significantly decrease cardiovascular mortality and rehospitalization rates [11]. Clinical studies also found that treatment with QLQX shows superior performance to a placebo treatment in improving functional class, left ventricular ejection fraction, 6-minute walk distance, and quality of life in accordance with the criteria of the New York Heart Association [12]. These results clearly demonstrate the protective effect of QLQX against cardiovascular diseases. Furthermore, recent studies have shown that QLQX can affect ventricular remodeling by regulating collagen deposition. However, the specific mechanism by which QLQX modulates collagen homeostasis remains unclear.

**Table 1:** The components of Qili Qiangxin capsule.

Scientific name	Local name	Family	Amount
*Astragalus membranaceus (Fisch*.*) Bge*. *var*. *mongholicus (Bge*.*) Hsiao*	Huangqi	*Fabaceae*	450g
*Panax ginseng C*. *A*. *Mey*	Renshen	*Araliaceae*	225g
*Salvia miltiorrhiza Bunge*	Danshen	*Lamiaceae*	225g
*Alisma orientale (Sam*.*) Juzep*	Zexie	*Alismataceae*	225g
*Rhizoma Atractylodis Macrocephalae*	Xiangjiapi	*Fabaceae*	180g
*Lepidium apetalum Willd*	Tinglizi	*Brassicaceae*	150g
*Aconitum carmichaelii Debx*	Fuzi	*Ranunculaceae*	112.5g
*Cinnamomum cassia (L*.*) J*. *Presl*	Guizhi	*Lauraceae*	90g
*Carthamus tinctorius L*.	Honghua	*Asteraceae*	90g
*Citrus reticulata Blanco*	Chenpi	*Rutaceae*	75g
*Rhizoma Polygonati*	Yuzhu	*Asparagaceae*	75g

The main objective of this study was to investigate the mechanism by which QLQX improves MF through the regulation of collagen homeostasis. To achieve this objective, a comprehensive approach was employed. Firstly, a rat model of MI was induced by ligation of the left anterior descending (LAD) artery to investigate the anti-MF mechanism of QLQX post-MI. The study aimed to elucidate the specific role and possible mechanisms underlying the changes in collagen balance. Subsequently, To evaluate the effectiveness of QLQX, several assessments were performed. Cardiac function and the extent of fibrosis were evaluated to determine the impact of QLQX treatment. The picrosirius-polarization method and Western blot analysis were utilized to examine the types and content of collagen. Furthermore, the expression levels of key proteins involved in collagen metabolism, including metalloproteinase MMP-9, metalloproteinase inhibitor TIMP-1, as well as AT1 and α-SMA, were examined in myocardial tissue. In summary, this study employed a multi-faceted approach and various assessments to investigate the potential of QLQX in improving MF by modulating collagen homeostasis.

We hypothesized that myocardial collagen content is tightly regulated by the balance between collagen production and degradation. In this study, we aimed to investigate the effect of QLQX on collagen fibers and elucidate its mechanism of action in rats after MI. This study provides novel insights into the mechanism by which QL prevents MF after MI.

## Materials and methods

### Animals

Specific Pathogen Free male Sprague-Dawley (SD) rats aged 5–6 weeks old and weighing 180 ± 20 g were acquired from Hunan Slaik Jingda Experimental Animal Co., Ltd. (certificate No. SCXK (Xiang), 2021–0004). The rats were maintained in a temperature-controlled room (23 ± 2°C) with a 12/12 h light-dark cycle and sufficient food and water. All procedures involving the care and use of animals were carried out in accordance with the Guidelines for the Human Use and Care of Laboratory Animals for Biomedical Research. The experimental protocol was approved by the Ethical Animal Committee of Jiangxi University of Chinese Medicine(No.JZLLSC2023-0702), China.

### Drugs and reagents

QLQX Capsule (batch number: Z20040141) were provided by Shijiazhuang Yiling Pharmaceutical Corporation (Shijiazhuang, China). Empagliflozin (batch number: Z20049861) was purchased from Beijing Novartis Pharmaceuticals Co., Ltd. (Beijing, China). Hematoxylin and eosin (H&E) staining solution (batch number:717112, 717113) was purchased from Zhuhai Beso Biotechnology Co., Ltd. (Zhuhai, China). Masson’s staining (batch number: G1340)and Picrosirius red (PSR) solution(batch number: G1472)were purchased from Beijing Solarbio Biotechnology Co., Ltd. (Beijing, China). High-sensitivity cardiac troponin I (hs-cTnI) and N-terminal B-type natriuretic peptide (NT-proBNP) kits (batch number:20211215,30112A) were purchased from Shanghai Enzyme Link Biotechnology (Shanghai, China). Tissue inhibitor of matrix metalloproteinase-1 (TIMP-1), metalloproteinase (MMP)-9, collagen II, collagen III, collagen IV, angiotensin type 1 receptor (AT1), TGF-β1, and α-smooth muscle actin (α-SMA) antibodies (batch number:WL02342, WL03096, WL03082, WL03186, WL02974, WL02193, WL02510) were purchased from Shenyang Wanleibio Biotechnology Co., Ltd.(Shenyang, China). Collagen IV (batch number: A24008) were purchased from Abclonal Biotechnology Co., Ltd. (Wuhan, China). Collagen I (batch number: GB114197), GAPDH (batch number: GB15002), bicinchoninic acid (BCA) protein assay kit (batch number: G2026), horseradish peroxidase (HRP)-labeled goat anti-mouse (batch number: GB23301) and HRP—labeled goat anti-rabbit antibodies (batch number: GB23303) were purchased from Wuhan Servicebio Biotechnology Co., Ltd. (Wuhan, China). Ultra-sensitive ECL chemiluminescence kit (batch number: MA0186) was purchased from Dalian Meilun Biotechnology Co., Ltd.(Dalian, China). Polyvinylidene fluoride (PVDF) membranes (batch number: R9M2784) were purchased from Merck Millipore (Germany).

### Establishment of MI model and grouping

The rats were subjected to ligation of the left anterior descending (LAD) coronary artery to establish an MI model. In brief, the rats were anesthetized by intraperitoneally injecting of 1% pentobarbital sodium (40 mg/kg) and then administering a small animal ventilator (5:4 respiratory ratio) for mechanical ventilation. The heart was exposed with a horizontal incision through the muscle between the 3–4 intercostal space of the left sternal border, and the LAD coronary artery was perennially ligated with a 6–0 silk surgical thread. After surgical ligation of the coronary artery, the color of the anterior wall of the left ventricle turned gray or cyanotic, the ventricular wall motion decreased and the electrocardiogram (ECG) showed ST-segment elevation, indicating successful establishment of the MI model. Penicillin was routinely injected for 3 consecutive days after surgery to prevent infection. The Rats with LAD ligation were randomized and assigned to the low-concentration QLQX group (n = 6, QL-L), the high-concentration QLQX group (n = 6, QL-H), the empagliflozin group (n = 6), or model group (n = 6). Rats that underwent the same surgical procedure but without LAD ligation were assigned to the sham group (n = 6). As a marketed Chinese medicine formula, the adult clinical dose of QLQX is 0.056 g/(kg•day) [13]. The studies have shown that the drug exhibits efficacy and safety in the range of 0.25–1.2 g/(kg•day) [14]. In the present study, 0.6 g/(kg•day) QLQX (2 times the clinical dose) was administered as the low-dose group, and 1.2 g/(kg•day) (4 times the clinical dose) was administered to the high-dose group to observe the quantitative-effect relationship. Empagliflozin was converted to 1.0 mg per day in rats for subsequent experiments according to the previous study [15]. The rats in the sham and model groups were intragastrically administered with similar volumes of saline. After 28 days, the rats were anesthetized by intraperitoneally injecting of 1% (w/v) sodium pentobarbital (40 mg/kg) at a uniform rate. It was not until the rats were unconscious that the process began, and then the blood samples and myocardial tissue specimens were harvested. Part of the myocardial tissue was excised and fixed in 4% (w/v) paraformaldehyde and embedded in paraffin for H&E, Masson, PSR, and immunofluorescence (IF) staining, and the rest was stored in the refrigerator at -80°C for western blotting (WB) analysis.

### Determination of heart size and mass index

The body mass (BM) of each rat was measured and recorded before autopsy. After necropsy, the hearts were quickly removed on ice, excised, rinsed in pre-cooled saline, and then dried with a filter paper. The length, width, and thickness of the heart were measured. Heart mass (HM) was measured, and the heart mass index (HMI) = HM (mg)/BM (g) was calculated to assess the impact on the development of cardiac hypertrophy.

### ECG and echocardiography

The ECG of the rats in each group was examined before molding, after molding, and before autopsy. After 28 days of QLQX treatment, echocardiography was performed to assess cardiac function. The rats were anesthetized with 3% isoflurane and maintained with 1.5% isoflurane in an inhalation chamber during the examination. The rats were placed in supine position, the skin was prepared on the left anterior chest, and then the chest was applied with a coupling agent. Changes in the ventricular volume were evaluated by obtaining a long-axis section image of the parasternal left ventricle using a Vevo 2100 ultrasound imaging system (VisualSonics, Toronto, Canada) at a frequency of 15 MHz. The left ventricular end-diastolic diameter (LVIDd), left ventricular end-systolic diameter (LVIDs), left ventricular fraction shortening (LVFS), and left ventricular ejection fraction (LVEF) were measured for three cardiac cycles, and their mean values were calculated.

### Enzyme-linked Immunosorbent Assay (ELISA)

The blood samples were centrifuged at 2500 r·min−1 for 15 min at 15°C to obtain serum samples. Before the test, the supernatant was collected, promptly frozen at -80°C, and then thawed at 4°C. Serum levels of hs-cTnI and NT-proBNP were examined using an ELISA kit in accordance with manufacturer’s instructions.

### H&E staining

Myocardial tissue was fixed with 4% paraformaldehyde, dehydrated with gradient ethanol, diaphanized twice with xylene for 20 min each, embedded in paraffin, and then coronally sliced into 4-μm-thick sections. The sections were dewaxed twice with xylene for 10 min each, dehydrated with gradient ethanol. Finally, the sections were stained with Gill Hematoxylin for 10 min, rinsed with water, and then stained with 0.5% eosin solution for 1 min. After a final dehydration with gradient ethanol, the sections were sealed with neutral gum and observed under a light microscope to evaluate pathological changes.

### Masson staining

Masson staining was used to assess the severity of MF. Myocardial tissue was removed, fixed in 4% paraformaldehyde, dehydrated with alcohol, cleared with xylene, embedded in paraffin, and then sliced into 5-μm-thick sections. The sections were stained with Masson staining solution. The specific operational steps were performed in strict accordance with the manufacturer’s instructions. Images were acquired using an inverted optical microscope. The myocardial tissues were stained red, and collagen fibers were stained blue. The collagen volume fraction (CVF) of rat myocardial tissue was analyzed using ImageJ software (NIH Image, Bethesda, MD) with the following formula: CVF = myocardial collagen fiber area/(collagen area + myocardial tissue area)× 100.

### PSR staining

Paraffin-embedded heart specimens were sliced into 5-μm-thick sections. The sections were mounted onto glass slides and stained with PSR solution to detect MF-associated proteins, including collagen I, II, III and IV. The specific operating methods were performed in strict accordance with the manufacturer’s instructions and related literature [16]. The samples stained with PSR were examined using optical microscope (LeicaDL2500, Germany) and polarizing microscope (Leica DM2700P, Germany). The mean optical densities (MOD) of collagen types I, II, III and IV were analyzed using the Image-Pro Plus 6.0 (Media Cybernetics, Inc., Rockville, MD, USA).

### IF staining

After being dewaxed, rehydrated, and blocked, 5-μm-thick sections of myocardial tissue were incubated with MMP-9 and TIMP-1 antibodies overnight at 4°C and then with fluorescent-conjugated secondary antibodies. The nuclei were counterstained with DAPI. Images were observed using fluorescent microscope and ImageJ software was used for semi-quantitative analysis.

### Western blot

Myocardial tissue samples were added to protein lysis buffer and ultrasonically pulverized. After lysis on ice for 30 minutes, the protein concentrations in the supernatants of the tissue lysates were quantified using a BCA protein assay kit. Proteins were separated by 10%-12% sodium dodecyl sulfate-polyacrylamide gel electrophoresis and then transferred onto PVDF membranes. The membranes were incubated for 2 hours with 5% skim milk powder. Primary antibodies for collagen I(dilution 1:2000), collagen II (dilution 1:1000), collagen III (dilution 1:1000), collagen IV (dilution 1:1000), AT1 (dilution 1:1000), TGF-β1 antibody (dilution 1:1000), α-SMA antibody (dilution 1:1000) and anti-GAPDH (dilution 1:2000) were added and incubated at 4°C overnight. The membranes were then incubated with HRP-labeled goat anti-mouse or goat anti-rabbit IgG secondary antibodies (dilution 1:5000) for 2h at room temperature. The bands were visualized using an ECL luminous reagent, and an automatic gel imaging system (Bio-Rad, USA) was used for exposure imaging. The expression of protein bands was determined using ImageJ. The expression levels of collagen I-IV, α-SMA, AT1 and TGF-β1 were expressed by the ratio of the gray value of the target protein to the internal reference protein GAPDH. Two samples were collected from each group for analyses.

### Statistical analysis

SPSS 26.0 software (SPSS Inc., Chicago, IL, United States) and GraphPad Prism software 9.0 (GraphPad Software Inc.) were used for statistical analysis. Data are expressed as mean ± standard deviation. Statistical analysis was performed using one-way ANOVA between multiple groups, Fisher’s least significant difference test was used for two-by-two comparisons when the variances were in agreement, whereas Dunnett’s T3 test was used when the variances were not in agreement. Statistics were deemed significant at *P* < 0.05.

## Results

### Changes in heart volume and mass index

**Fig 1:**
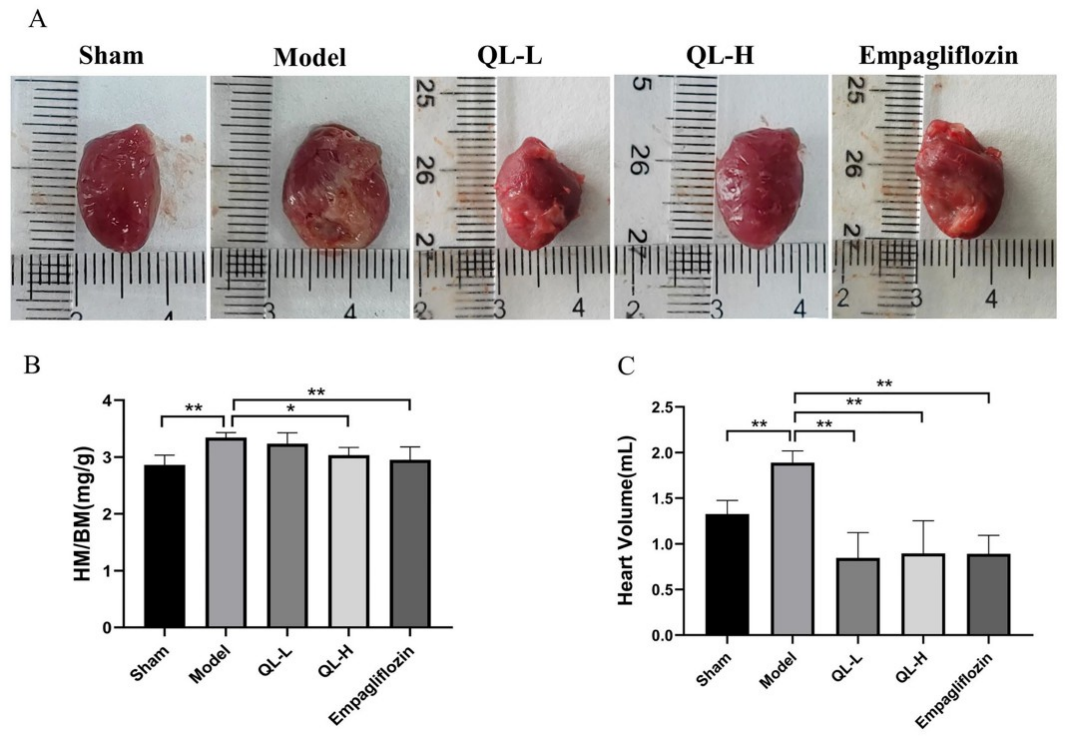
Qili Qiangxin capsule (QLQX) improved heart morphology. **A,** Representative images of heart morphology. **B,** Quantitative results of the heart mass index (HMI) in each group. **C,** Measured results of heart volume in each group. * *P*<0.05, ** *P*<0.01. Data were shown as the mean ± SD, n = 6 in each group, and all by analysis of variance.

The HMI and heart volume were significantly higher in the model group than in the sham group, indicating that the hearts of the rats in the model group were enlarged (Fig 1A–1C, *P* < 0.01). Compared with the model group, the QLQX-H and empagliflozin groups showed significantly lower HMI and heart volume (Fig 1B and 1C, *P* < 0.05 or *P* < 0.01), and the QLQX-L group had significantly lower heart volume (*P* < 0.01)but no significantly different HMI.

### Changes in ECG and echocardiography

**Fig 2:**
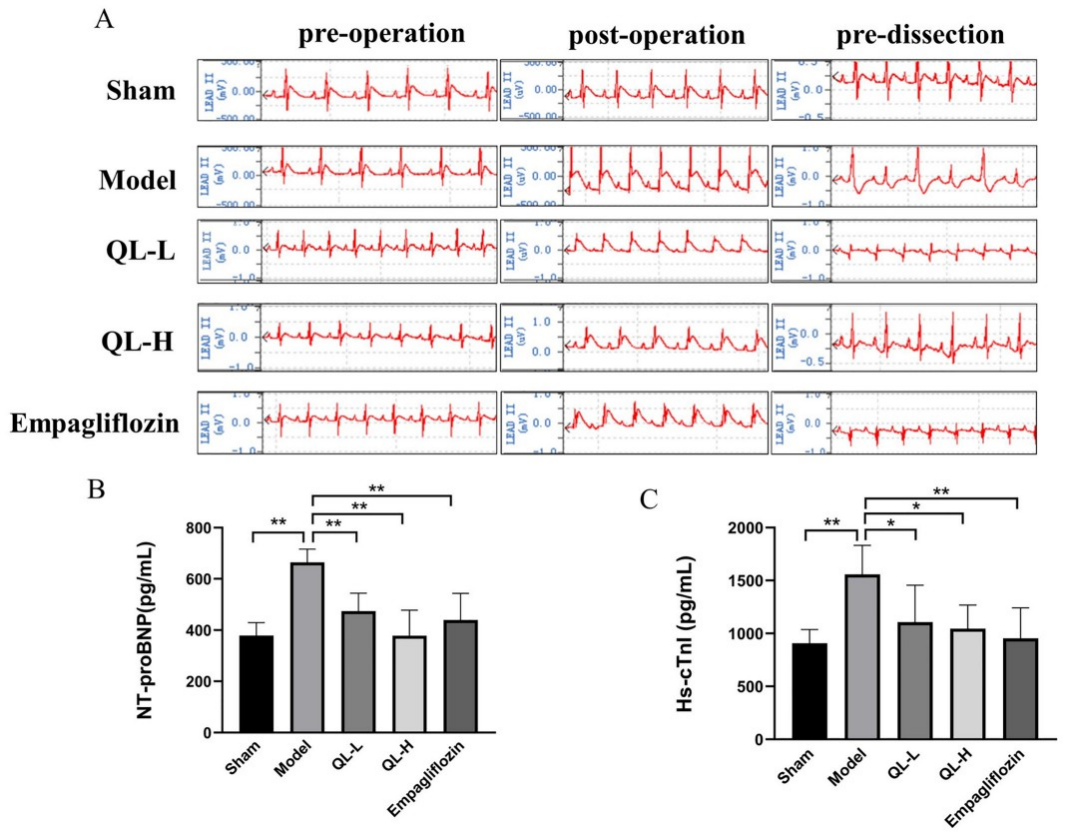
QLQX improved electrocardiographic manifestation and cardiac biochemical parameters. **A,** Representative images of electrocardiogram (ECG), the elevated ST-segment of lead II returned to baseline level, and the T wave returned to normal after treatment in the administered group. **B,** N-terminal B-type natriuretic peptide (NT-proBNP) levels. **C,** High-sensitivity cardiac troponin I (hs-cTnI) levels. * *P*<0.05, ** *P*<0.01. Data were shown as the mean ± SD, n = 6 in each group, and all by analysis of variance.

The ECG of the rats in each group was examined before surgery, 10 min after surgery, and before autopsy. After successful modeling of the MI, the ST segments in lead II were elevated upward and the T waves were elevated compared with those before surgery. After 28 days of drug administration, the ST segments of the drug-dosing groups were significantly depressed and approached baseline levels (Fig 2A).

### Expression of cardiac biochemical parameters in ELISA

Significant alterations in cardiac biochemical parameters NT-proBNP and hs-cTnI were observed. The serum levels of NT-proBNP (Fig 2B) and hs-cTnI (Fig 2C) were significantly higher (*P* < 0.01) in the model group than in the sham group. Compared with the model group, the QLQX-L, QLQX-H and empagliflozin groups had significantly lower serum levels of NT-proBNP (*P* < 0.01), and also showed a certain degree of hs-cTnI in the QLQX-L, QLQX-H (*P* < 0.05) and empagliflozin (*P* < 0.01) groups.

### Changes in echocardiography

**Fig 3:**
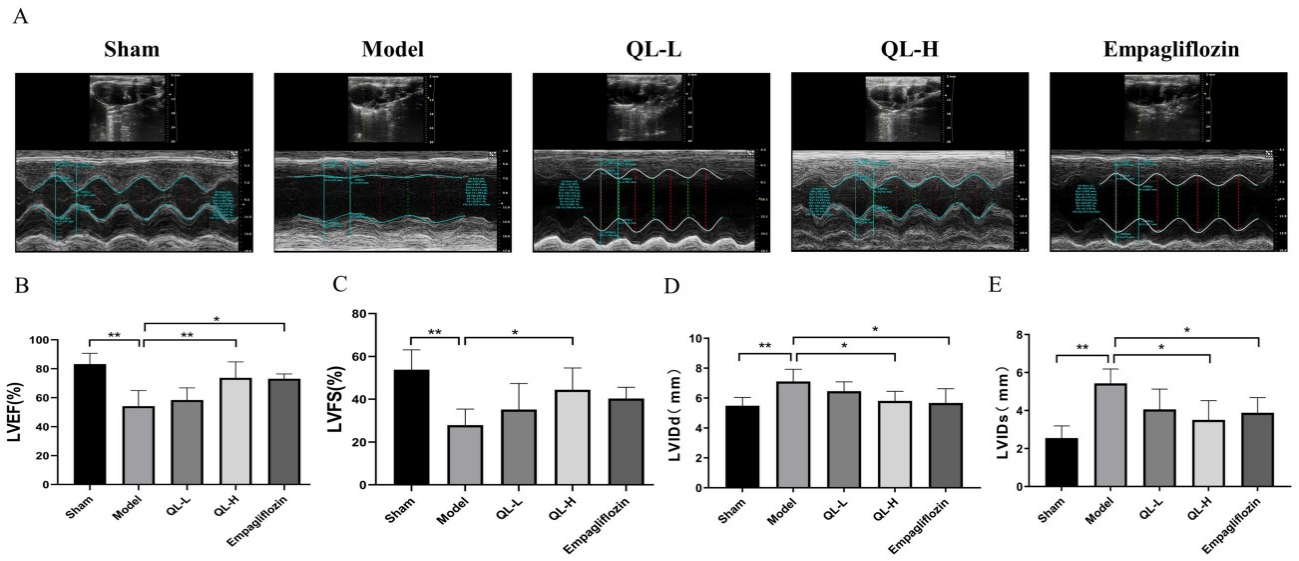
QLQX alleviated left ventricular dysfunction by echocardiography analysis. **A,** Representative echocardiogram of each group. **B,** Left ventricular ejection fraction (LVEF). **C,** Left ventricular fraction shortening (LVFS). **D,** Left ventricular end-diastolic diameter (LVIDd). **E,** Left ventricular end-systolic diameter (LVIDs). * *P*<0.05, ** *P*<0.01. Data were shown as the mean ± SD, n = 6 in each group, and all by analysis of variance.

The echocardiographic data (LVEF, LVFS, LVIDd, and LVIDs) and images of rats in the model group significantly differed from those of the rats in the sham group (Fig 3A–3E, *P* < 0.01), indicating was successful establishment of the MI model. Compared with the model group, the QLQX-H group had significantly lower LVEF and LVFS, and empagliflozin groups had significantly lower LVEF but no significantly different LVFS (Fig 3B and 3C, *P* < 0.05 or *P* < 0.01) The LVIDd and LVIDs in the QLQX-H and empagliflozin groups increased significantly (Fig 3D and 3E, *P* < 0.05), suggesting that treatment with QLQX-H or empagliflozin ameliorated cardiac function in the MI rats.

### Effect of QLQX in H&E staining

**Fig 4:**
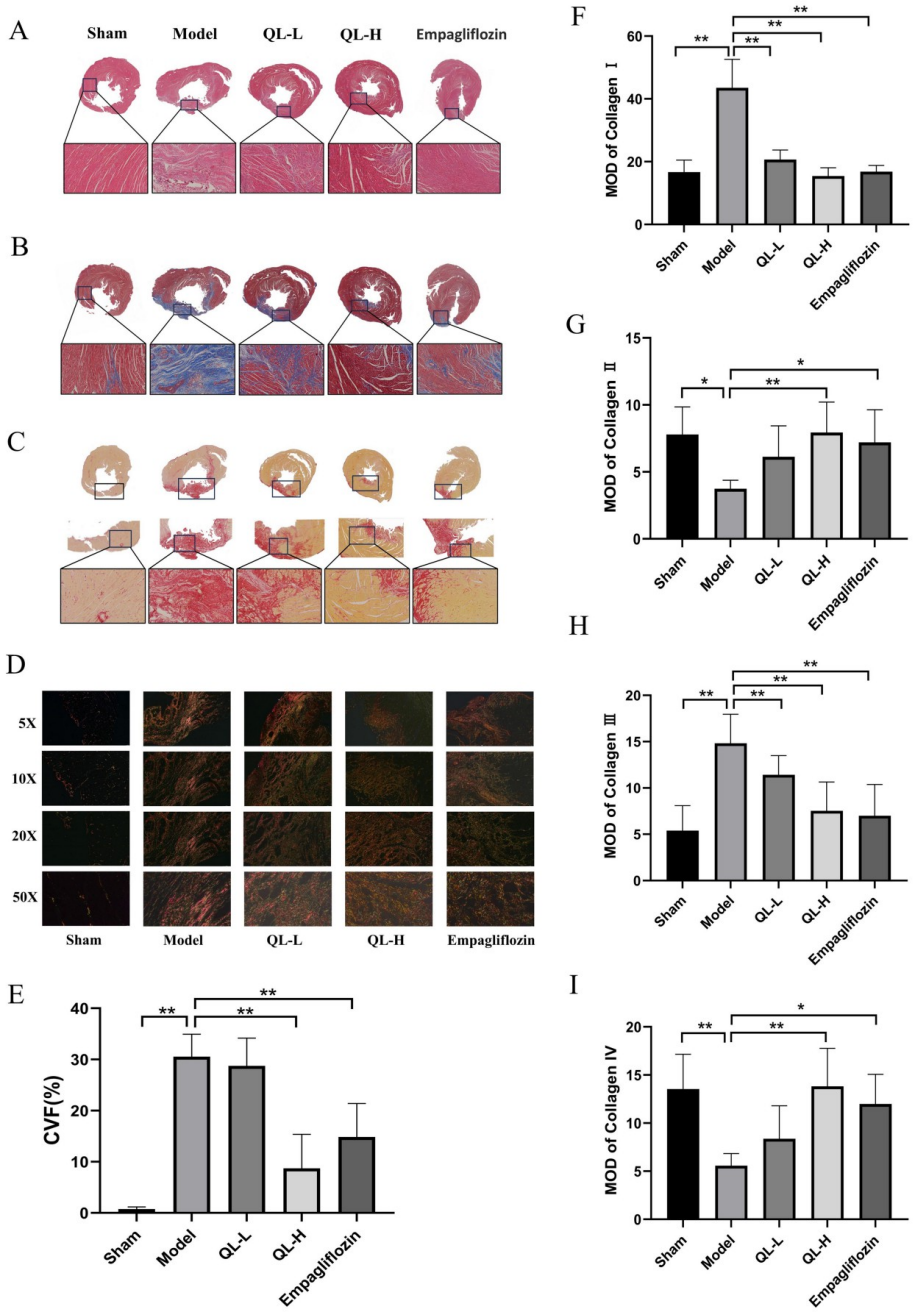
Histopathological changes in hematoxylin and eosin (H&E), Masson, and Picrosirius red staining (PSR). **A,** Representative images of H&E staining in each group. **B,** Representative images of Masson’s trichrome staining. The blue-stained positive area was collagen fibers in the heart tissues. **C,** PSR staining results obtained using an optical microscope. The red-stained positive area indicated fibrosis in the heart tissues.**D,** Representative images of Picrosirius red staining in a polarizing microscope. **E,** The results of collagen volume fraction (CVF) in Masson’s staining. **F-I,** The mean optical density (MOD) of collagen I, II, III and IV in polarizing light microscope. * *P*<0.05, ** *P*<0.01. Data were shown as the mean ± SD, n = 3 in each group, and all by analysis of variance.

Histopathological examination of the H&E-stained slides (Fig 4A) revealed clear differences among these groups. The heart sections from the sham group displayed normal architecture with intact cardiac muscle fibers, characterized by well-defined striations and intact cell membranes. By contrast, the myocardial cells in the model group were hypertrophied and disordered with enlarged cell gaps, some lost cell nuclei, and atrophied myocardial tissue in the infarcted area. Meanwhile, the myocardial tissue from the QLQX and empagliflozin groups did not display these histopathological alterations. Specifically, the sections from the QLQX and empagliflozin groups exhibited reduced necrosis, increased degree of angiogenesis, smaller myocardial interstitial space, enhanced maintenance of relative structural integrity, and reduced myocardial damage compared with those from the model group.

### QLQX alleviated myocardial fibrosis in Masson staining

Masson staining results revealed a greater number of the blue-stained collagen fibers, with fibrous scar formation and a larger myocardial collagen area in the model group than in the sham group (Fig 4B and 4E, *P*< 0.01). Compared with the model group, the QLQX-H and empagliflozin groups had smaller CVF (Fig 4E, *P* < 0.01), suggesting that treatment with QLQX-H or empagliflozin alleviated MF. However, no significant differences were found between the QLQX-L and model groups (*P*> 0.05).

### Collagen content changes in PSR staining

PSR staining was used for the quantitative analysis of collagen content in the paraffin-embedded heart tissue, This method relies on the birefringence of collagen fibers, which can highly and specifically underline fibers when combined with polarized light microscopy [17]. Under an optical microscope, the collagen fibers appeared red, the nuclei appeared black, and the remainder appeared yellow. Difference in birefringence and coloring can reveal four types of collagen fibers under a polarizing microscope. Under polarized light illumination, collagen I was tightly arranged, with strong birefringence and green-red coarse fibers. Collagen II-IV showed weak birefringence. Collagen II appeared as orange loosely reticulated fibers, collagen III as green fine fibers, and collagen IV as pale yellow fibers.

PSR staining under an optical microscope showed that compared with the sham group, the model group had more red-stained collagen fibers (Fig 4C). Myocardial collagen fibers were alleviated in the QLQX-H and empagliflozin groups, especially in the QLQX-H group (Fig 4C). PSR staining under a polarizing microscope showed that compared with the sham group, the model group had significantly higher contents of collagen I and III (Fig 4F and 4H, *P* < 0.01) but lower contents of collagen II and IV (Fig 4G and 4I, *P* < 0.05). Compared with the model group, the QLQX-L, QLQX-H and empagliflozin groups had significantly lower contents of collagen I and III (Fig 4F and 4H, *P* < 0.01). Meanwhile, the contents of collagen II and IV (Fig 4G and 4I) were significantly lower in the QLQX-H (*P* < 0.01) and empagliflozin groups (*P* < 0.05) than in the model group.

### Expression of MMP-9 and TIMP-1 in IF staining

**Fig 5:**
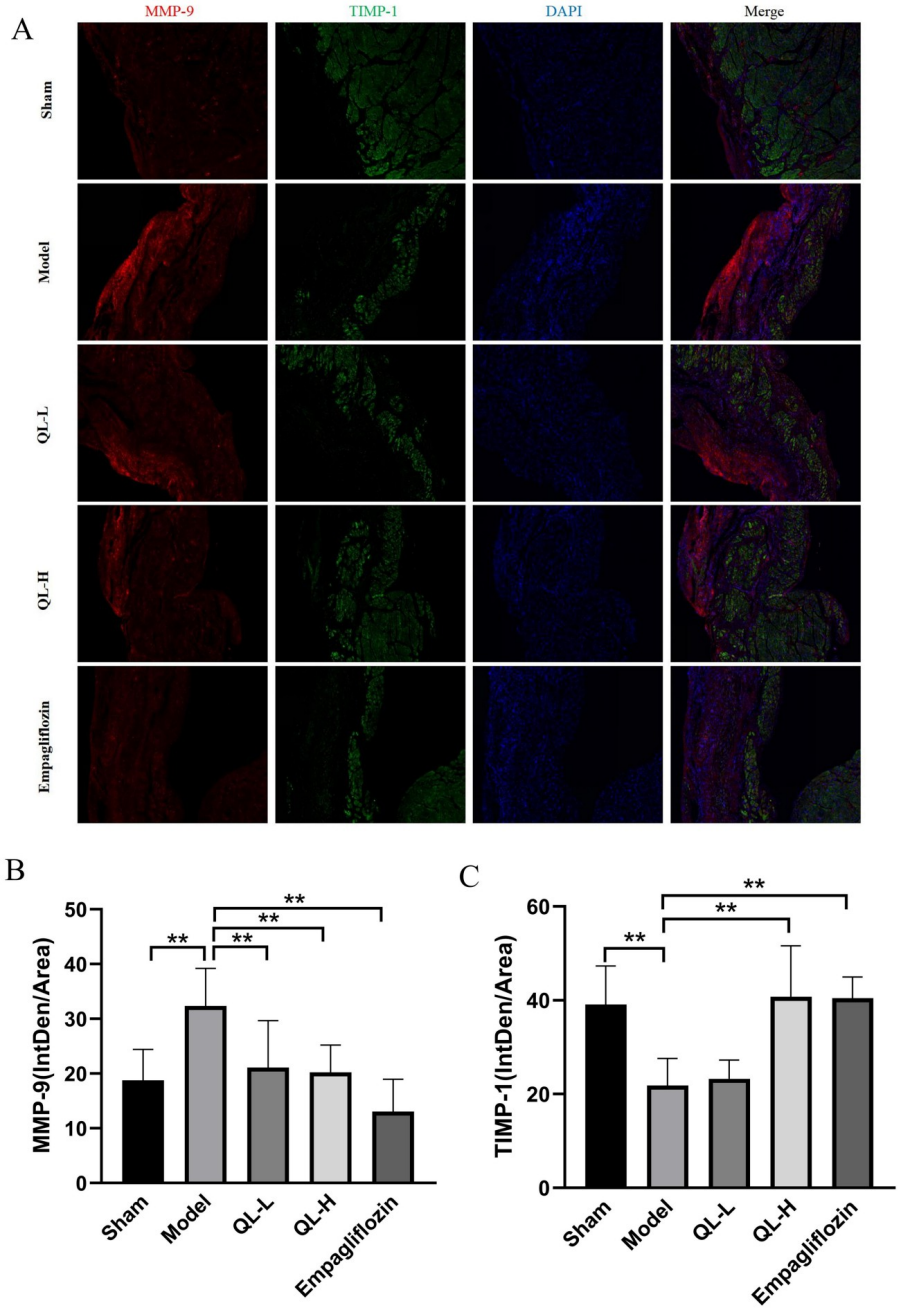
Expression changes of metalloproteinase (MMP)-9 and tissue inhibitor of matrix metalloproteinase (TIMP)-1 in immunofluorescence (IF) staining. **A,** MMP-9 and TIMP-1 expression using IF staining.**B,** Mean fluorescence intensity (MFI) of MMP-9. IF staining showed that MMP-9 expression was significantly increased in the model group. After treatment with QLQX or empagliflozin, the expression of MMP-9 significantly decreased. **C,** MFI of TIMP-1. IF staining showed that TIMP-1 expression was significantly decreased in the model group. After treatment with QLQX or empagliflozin, the expression of TIMP-1 significantly increased. IntDen = integrated density. * *P*<0.05, ** *P*<0.01. Data were shown as the mean ± SD, n = 3 in each group, and all by analysis of variance.

MMPs are collagen-hydrolyzing enzymes that lead to ECM protein degradation, whereas TIMPs are endogenous MMP inhibitors that bind to MMPs in a 1:1 ratio [18]. Targeted knockdown of MMP-9 reduced the number of macrophages after MI, which attenuated LV enlargement, prevented collagen accumulation, promoted neovascularization, and improved LV remodeling [19, 20]. TIMP-1 is an endogenous regulator of MMP-9 activity. In the present study (Fig 5A–5C), compared with the sham group, the model group demonstrated significantly stronger mean fluorescence intensity (MFI) for MMP-9 and significantly lower TIMP-1 expression (*P* < 0.01), suggesting the development of MF. Compared with the model group, the QLQX-H and empagliflozin groups had significantly lower MMP-9 levels and higher TIMP-1 levels (*P* < 0.01), indicating that QLQX treatment can improve MF.

### Expression of collagen and pro-fibrotic cytokines in WB

**Fig 6:**
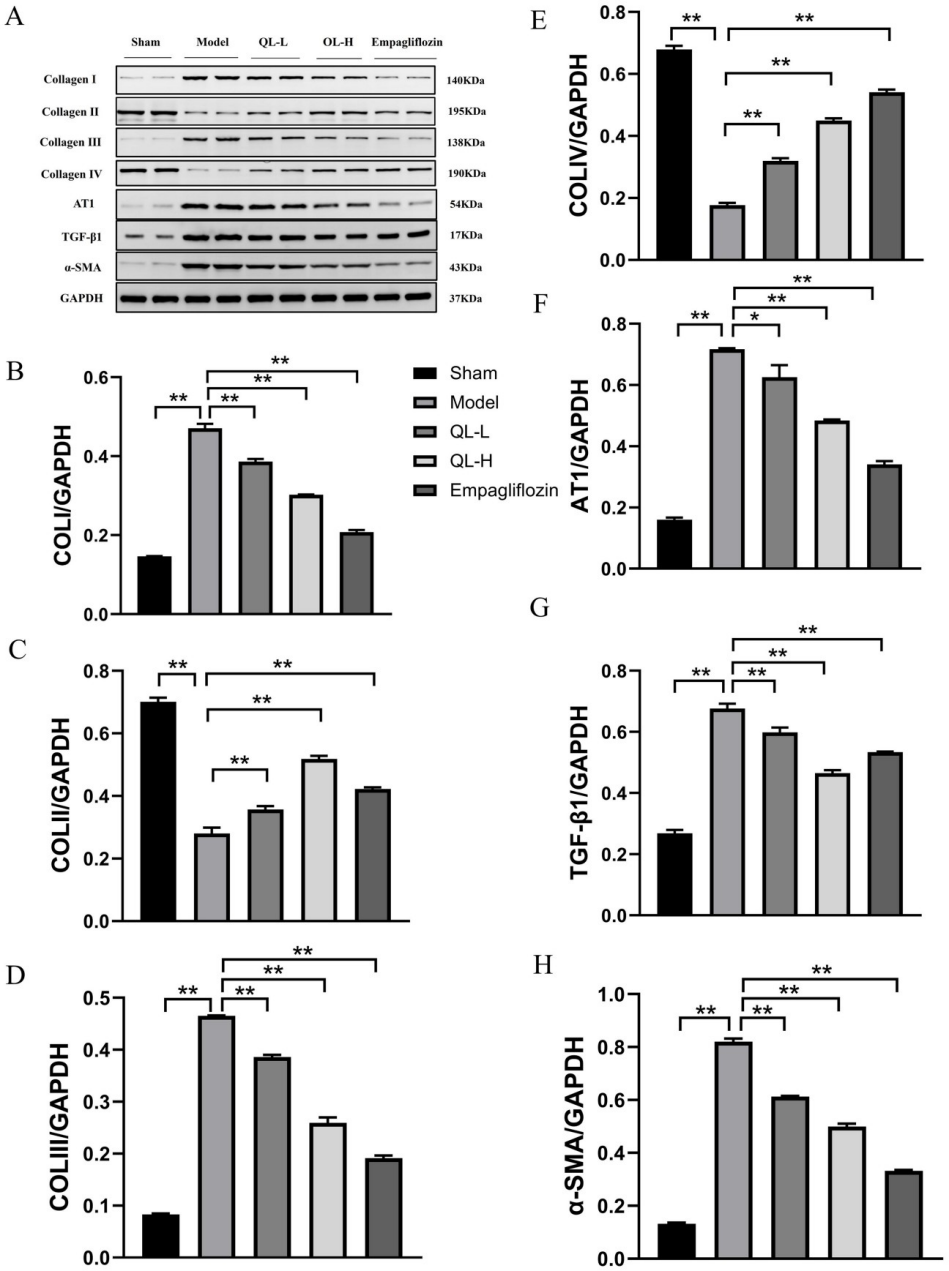
QLQX changed the expression of collagen and pro-fibrotic cytokines in western blotting. **A,** Expression of collagen I–IV, α-smooth muscle actin (α-SMA), angiotensin type 1 receptor (AT1) and transforming growth factor (TGF)-β1 in cardiac tissue. **B-H,** Protein contents of collagen I–IV, α-SMA, AT1 and TGF-β1 in myocardial tissue. * *P*<0.05, ** *P*<0.01. n = 3 per group. Data were shown as the mean ± SD, and all by analysis of variance.

As showed in Fig 6A, WB results suggested that compared with the sham group, the model group had significantly higher expression level of collagen I and III (Fig 6B and 6D, *P* < 0.01) but lower expression levels of collagen II and IV (Fig 6C and 6E). Compared with the model group, the QLQX-L, QLQX-H and empagliflozin groups showed significantly lower expression levels of collagen I and III (Fig 6B and 6D, *P* < 0.01), but significantly higher expression levels of collagen II and IV (Fig 6C and 6E, *P* < 0.01). These results were consistent with those of PSR staining under a polarizing microscope. WB results also revealed that the expression levels of TGF-β1, α-SMA, and AT1 were significantly higher in the model group than in the sham group (Fig 6F and 6G, *P* < 0.01). Meanwhile, the expression levels of these proteins were lower in the QLQX and empagliflozin groups than in the model group (Fig 6F and 6G, *P*< 0.01 or *P* < 0.05). TGF-β1 and α-SMA in the heart are involved in the proliferation and differentiation of fibroblasts to myofibroblasts [21], indicating that the QLQX treatment can suppress the transformation of fibroblasts to myofibroblasts.

## Discussion

MF is characterized by excessive deposition of ECM in myocardial tissues due to various pathological factors. In the early stage of heart injury, MF begins as an intrinsic response to protect the tissue from further damage and activate cardiac myofibroblasts, which secrete ECM proteins to replace damaged heart tissue [22]. Although initially beneficial, the repair process, if not adequately controlled, can lead to the replacement of normal tissue with permanent scar tissue and the disruption of myocardial cytoarchitecture, which can seriously affect cardiac function and result in HF [23]. MF also increases the load on the heart, leading to cardiac hypertrophy and stiffness, impairing the systolic and diastolic functions of the heart. The severity of MF is associated with poor prognosis and long-term mortality in many patients with heart disease, particularly HF [24]. Hence, novel therapeutic targets and effective therapeutic strategies to prevent MF progression after MI are urgently needed.

QLQX is a traditional Chinese medicine formula that has long been used to prevent and treat cardiovascular diseases. A previous study showed that QLQX can suppress MF and improve cardiac function via multiple pathways [25–27]. In the present study, we validated the therapeutic effects of QLQX on MF after MI and found through ECG, echocardiography and ELISA that QLQX can improve cardiac function. The levels of NT-ProBNP and hs-cTnI were markedly reduced in the QLQX groups compared with the model group (Fig 2). Consistently, the staining results indicated QLQX treatment attenuated cardiomyocyte hypertrophy (Fig 4). Furthermore, PSR staining and WB results showed that the model group had significantly higher contents of collagen I and III while lower contents of collagen II and IV than the sham group. MF was effective ameliorated in the QLQX and empagliflozin groups. Thus, myocardial collagen content was tightly regulated by the balance between collagen production and degradation.

Collagen is the most abundant protein in mammals, and since the discovery of type II collagen, 29 subtypes have been identified [28]. Collagen I and III play key roles in maintaining myocardial integrity and influencing cardiac compliance [29]. Collagen I is a rigid fibrillar protein that provides tensile strength, and increased collagen protein levels may cause myocardial stiffness and affect cardiac compliance [30]. In the present study, the decrease in collagen I content after treatment with QLQX improved cardiac compliance and ameliorated fibrosis. Collagen II is the main component of the cartilage matrix, which plays important roles in the induction of cartilage genesis, differentiation, and migration and in the regeneration of articular cartilage [31, 32]. In the present study, treatment with QLQX increased the collagen II content. Although its expression in cardiac tissue was low, we hypothesized that it was associated to some extent with ECM synthesis and remodeling. Collagen III is an elastic fiber that provides elasticity to the systolic-diastolic activity of the heart. However, collagen III is less stable than the other collagen types owing to its thinness. Excessive levels of collagen III promote localized vessel wall changes that lead to the formation of unstable atherosclerotic plaques and inflammation [33–35]. In the present study, treatment with QLQX decreased the level of collagen III, reducing the risk of vessel wall rupture and thus improving cardiac function. Collagen IV is a major component of the basement membranes in various tissues, including the arteries [36]. It acts as an ECM protein that provides support for cell adhesion, a key process in angiogenesis. The elevation in collagen IV level after treatment with QLQX may be related to the fact that it promotes the proliferation and differentiation of vascular endothelial cells, thereby promoting angiogenesis, which can improve blood supply to areas of MF and prevent its progression.

AT1 is a G protein-coupled receptor that regulates blood vessel wall pressure. It is mainly found in tissues and cells, such as vascular smooth muscle cells, in the cardiovascular system. When myocardial pressure is overloaded, AT1 receptors are activated, which can cause vasoconstriction and increase the cardiac load. Angiotensin II (Ang II), the main effector molecule of RAAS, exerts pro-fibrotic effects mainly through AT1 [37]. The binding of AT1 to AngII leads to myocardial hypertrophy and dysfunction, thereby accelerating the process of cardiac fibrosis [38]. Reducing the AT1 levels can significantly improve cardiac function and prevent HF progression [39]. Together with previous observations that pressure overload could activate and mediate AT1 [40], the present findings on the relationship between AT1 and collagen imbalance provide potential therapeutic targets for QLQX in treating MF.

This study utilized LAD to establish an MI model in rats and found that cardiac function was significantly reduced in the model group compared with the sham group. Echocardiography, ECG, gross cardiac structure, cardiac biochemical parameters (NT-proBNP and hs-cTnI), histopathology, and IF results (MMP-9 and TIMP-1) results indicated that QLQX treatment enhanced the cardiac function and alleviated myocardial tissues damage. Together with previous literature, we found that RAAS is important in MF treatment [37]. Thus, we hypothesized that collagen imbalance is also related to RAAS suffered by cardiomyocytes after infarction. We detected the expression of AT1 via WB. We also quantified collagen I–IV through PSR staining and validated the results using WB. Result revealed that QLQX treatment significantly decreased the levels of in collagen I and III, and increased the levels of collagen II and IV. Thus, QLQX treatment attenuated MF by modulating collagen homeostasis and alleviating the impairment caused by stress between cardiomyocytes.

In conclusion, This study investigated the effect of QLQX in the MI model. It was the first attempt to explore the effects of QLQX on collagen type and quantity in MF. Our results showed that QLQX ameliorated cardiac function and MF and prevented adverse LV remodeling in MI rats by downregulating AT1 expression, modulating collagen homeostasis between collagen I-IV. This study suggests that QLQX serve as a reliable agent for MF treatment and provides novel insights into its mechanism of action. However, further studies are warranted to completely elucidate the underlying mechanisms and explore the pharmacodynamics and pharmacokinetics of QLQX in *vitro*.

## Supporting information

S1 Fig Original images of rat heart appearance before combination for each group. (ZIP)

S2 Fig (A) Original electrocardiogram images of rats before combination for each group. (B) Original electrocardiogram images of rats in lead II.
(ZIP)

S3 Fig Original echocardiography images of the heart before combination for each group. (ZIP)

S4 Fig Original pathological staining images in HE staining. (ZIP)

S5 Fig Original pathological staining images in Masson staining. (ZIP)

S6 Fig Original pathological staining images under optical microscope in PSR staining. (ZIP)

S7 Fig Original pathological staining images of each group at various magnifications under polarized light microscope in PSR staining. (ZIP)

S8 Fig Original images of MMP9 and TIMP1 before combination for each group in IF staining. (ZIP)

S9 Fig The original, uncut full membrane data of the Western blot. (A) Duplicate expression images for each target protein band. (B)Single expression image for each target protein band.
(ZIP)

S10 Fig The underlying data for the AT1 gene expression results verified by qPCR. (ZIP)

S1 Data Original data underlying Figs 1B, 1C, 2B, 2C, 3B–3E, 4E–4I, 5B, 5C, 6B–6H and S10. (ZIP)

S1 Table The components of Qili Qiangxin capsule. (DOCX)

## Abbreviations

AT1: angiotensin type 1 receptor
BCA: bicinchoninic acid
BM: body mass
CVF: collagen volume fraction
ECG: electrocardiogram
ECM: extracellular matrix
ELISA: enzyme-linked Immunosorbent Assay
H&E: hematoxylin and eosin
HF: heart failure
HM: heart mass
HMI: heart mass index
HRP: horseradish peroxidase
hs-cTnI: high-sensitivity cardiac troponin I
IF: immunofluorescence
LAD: left anterior descending
LVEF: left ventricular ejection fraction
LVFS: left ventricular fraction shortening
LVIDd: left ventricular end-diastolic diameter
LVIDs: left ventricular end-systolic diameter
MF: myocardial fibrosis
MFI: mean fluorescence intensity
MI: Myocardial infarction
MMP-9: metalloproteinase-9
NT-proBNP: N-terminal B-type natriuretic peptide
PSR: Picrosirius red
PVDF: polyvinylidene fluoride
QL: Qili Qiangxin capsule
RAAS: renin–angiotensin–aldosterone system
TGF-β3: transforming growth factor-β3
TIMP-1: tissue inhibitor of matrix metalloproteinase-1
α-SMA: α-smooth muscle actin
