# Supplementary figures and images for: Qili Qiangxin capsule attenuates myocardial fibrosis by modulating collagen homeostasis post-infarction in rats
Source: PLoS One. 2024 Sep 27;19(9):e0310897. doi: 10.1371/journal.pone.0310897 (PMC11432860; doi:10.1371/journal.pone.0310897)

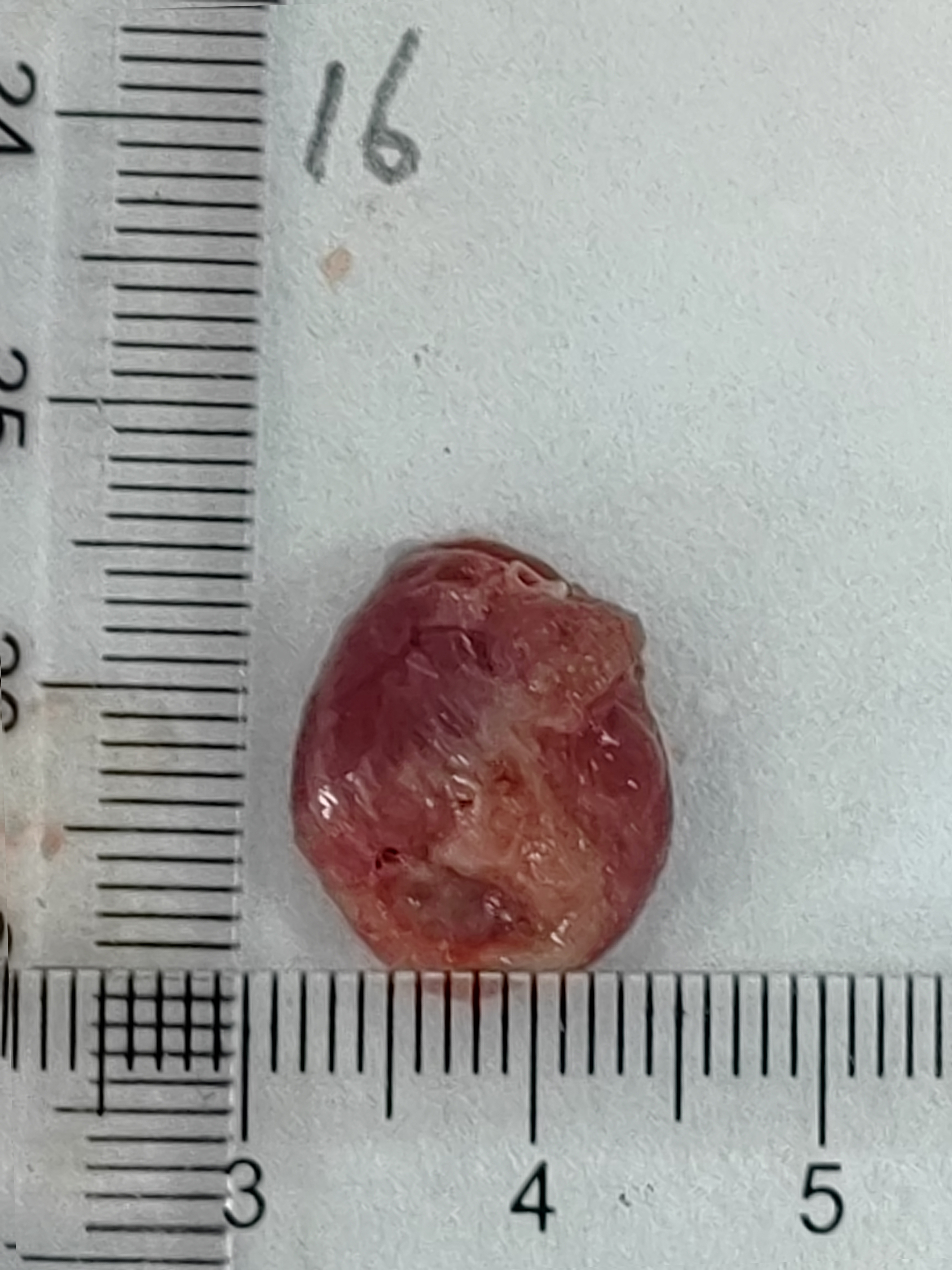

Supplement: S1 Fig — (ZIP) [file pone.0310897.s001.zip › S1 Fig/Fig1A Model.tiff]

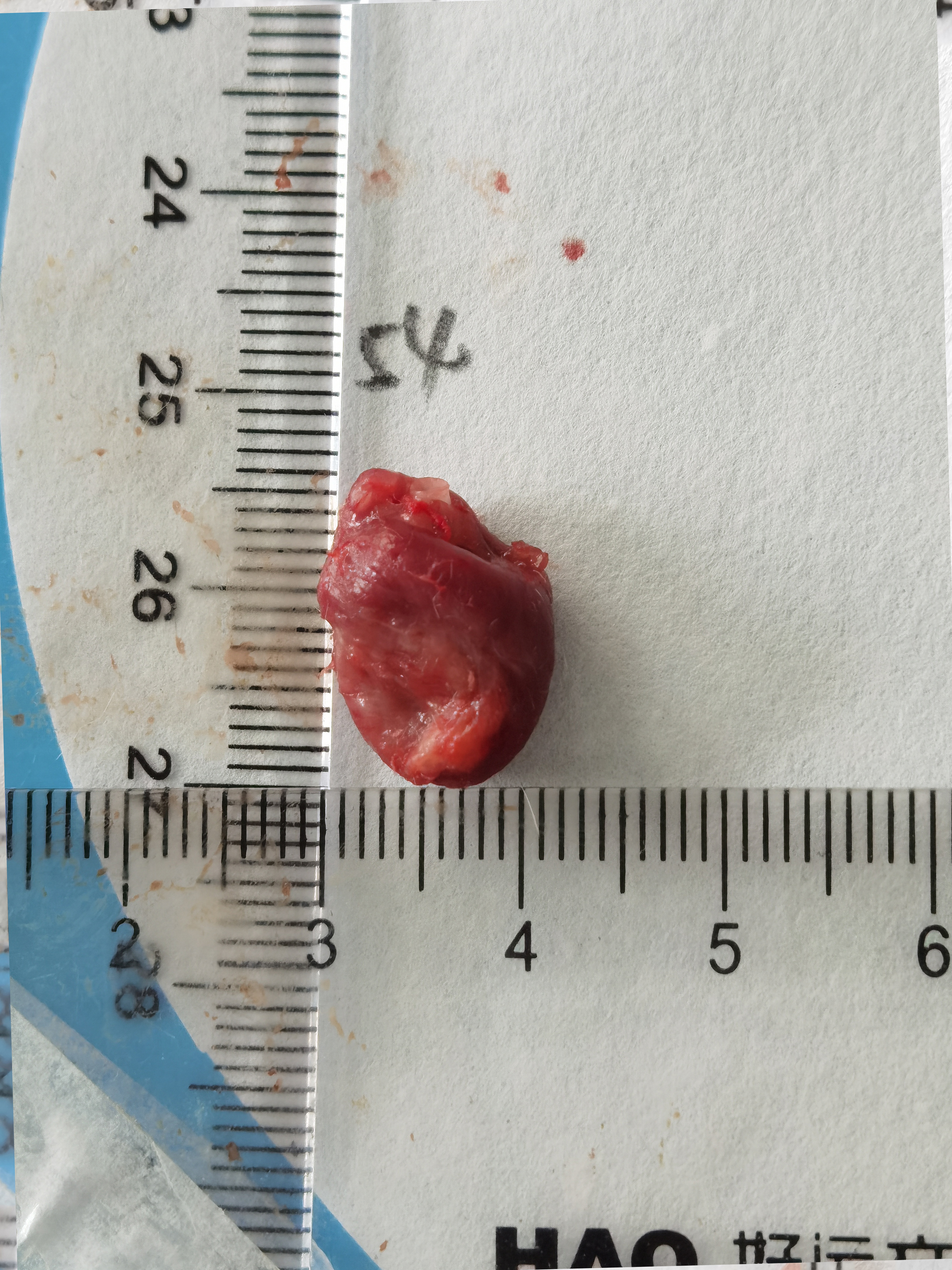

Supplement: S1 Fig — (ZIP) [file pone.0310897.s001.zip › S1 Fig/Fig1A Positive.tif]

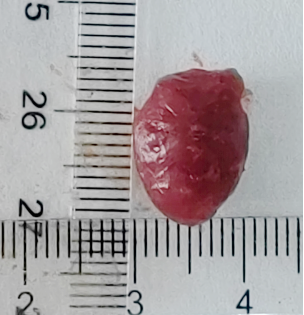

Supplement: S1 Fig — (ZIP) [file pone.0310897.s001.zip › S1 Fig/Fig1A QL-H.tif]

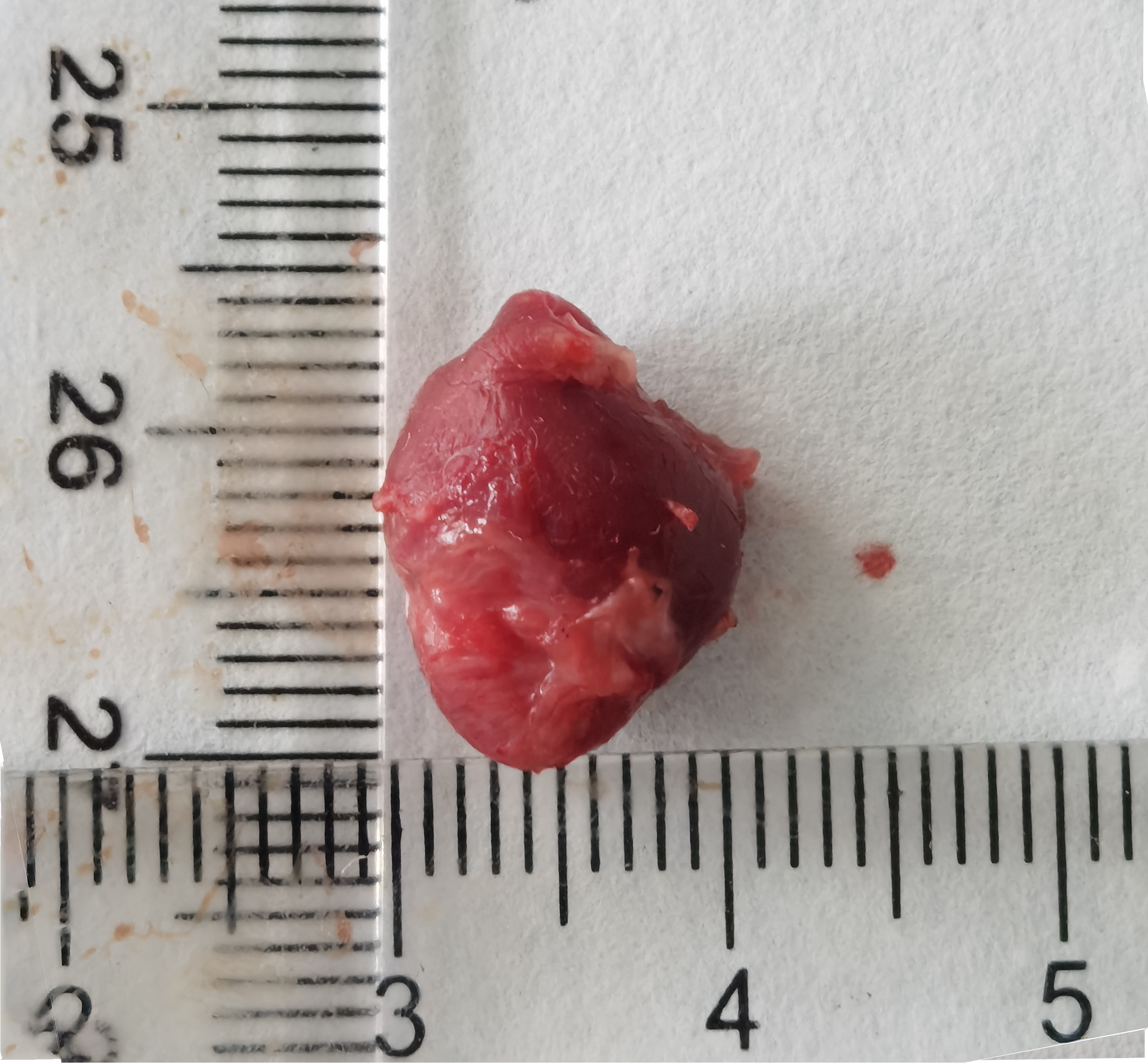

Supplement: S1 Fig — (ZIP) [file pone.0310897.s001.zip › S1 Fig/Fig1A QL-L .tif]

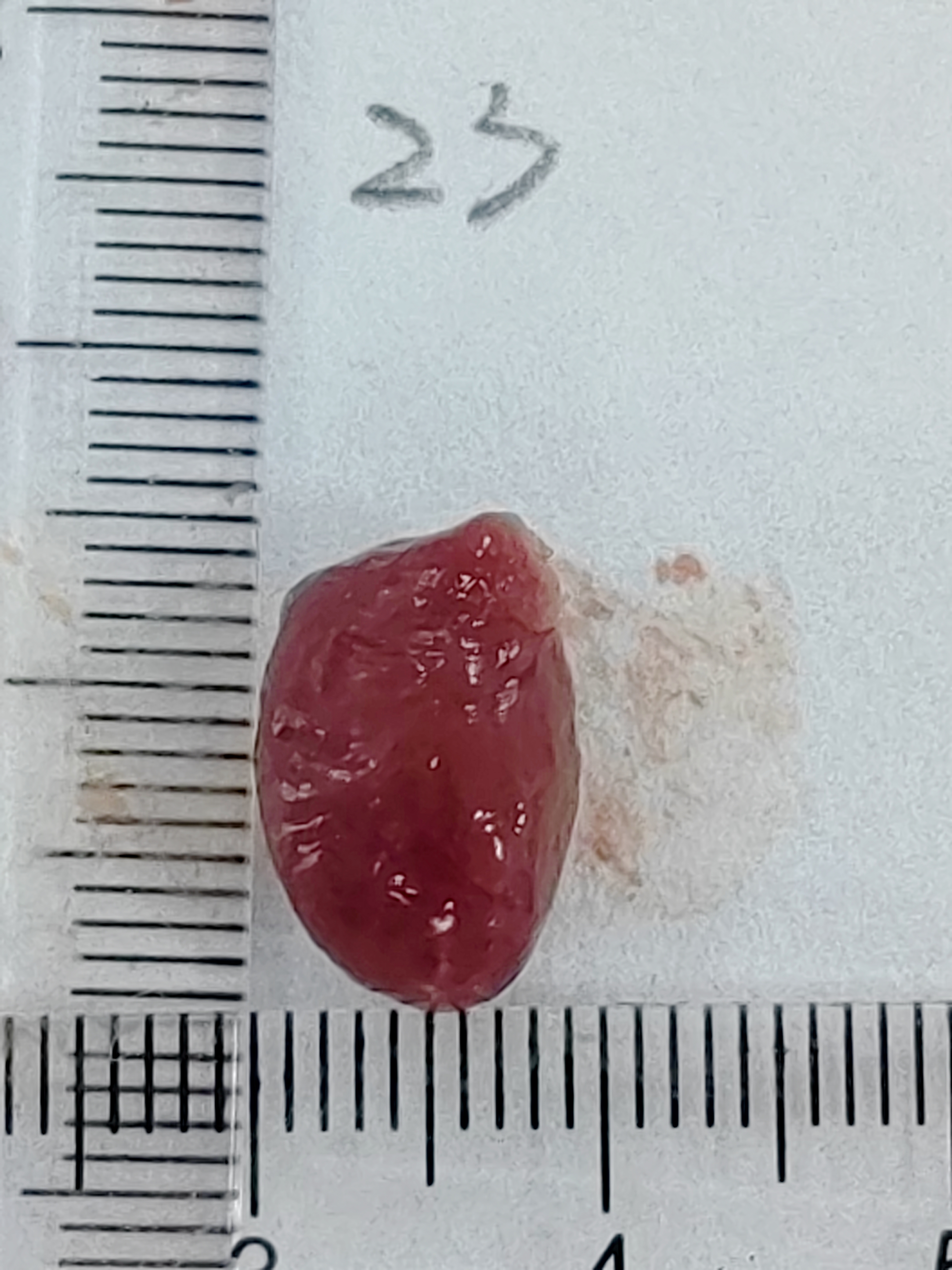

Supplement: S1 Fig — (ZIP) [file pone.0310897.s001.zip › S1 Fig/Fig1A Sham.tif]

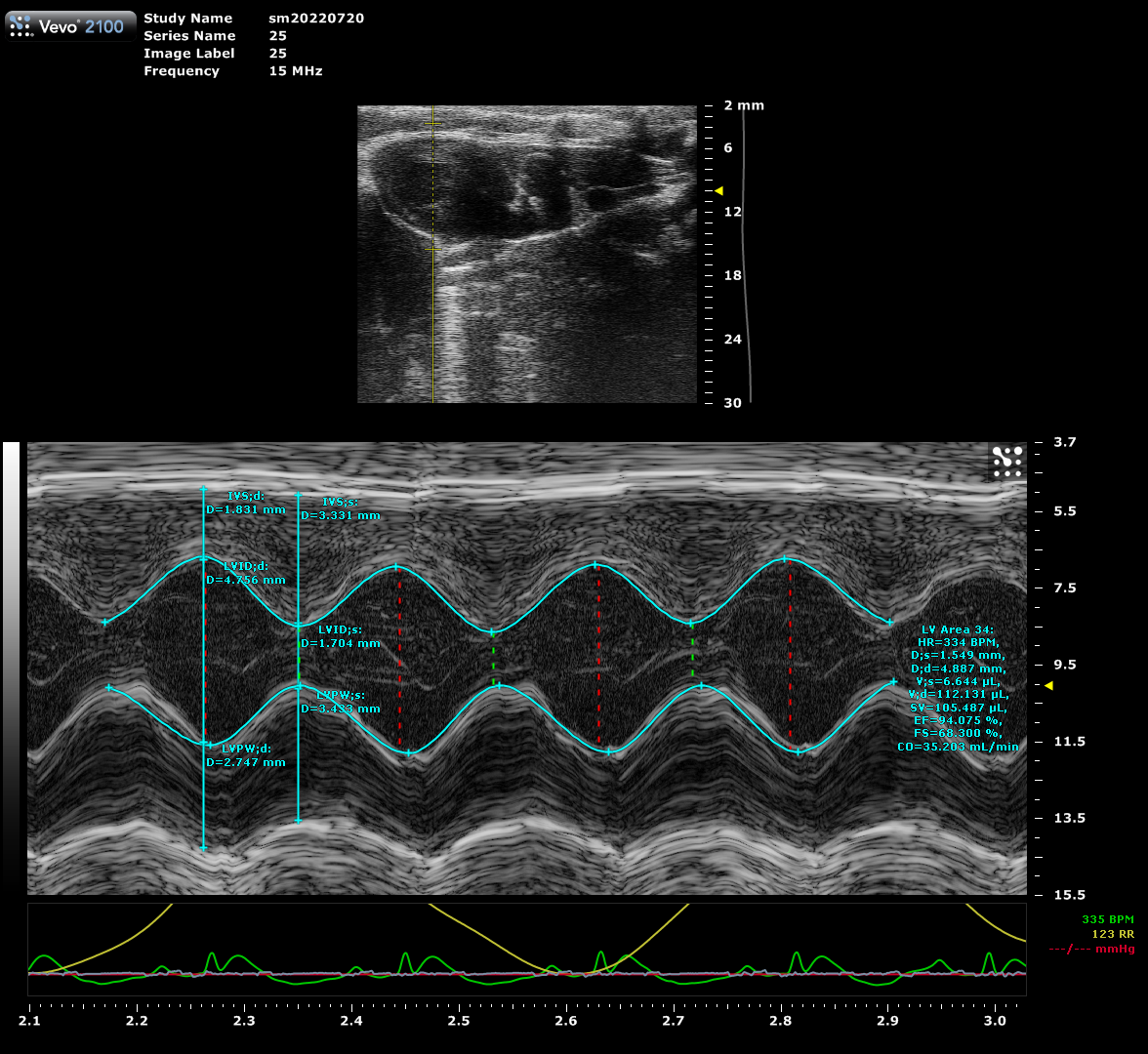

Supplement: S3 Fig — (ZIP) [file pone.0310897.s003.zip › S3 Fig/Fig3A1 Sham.tif]

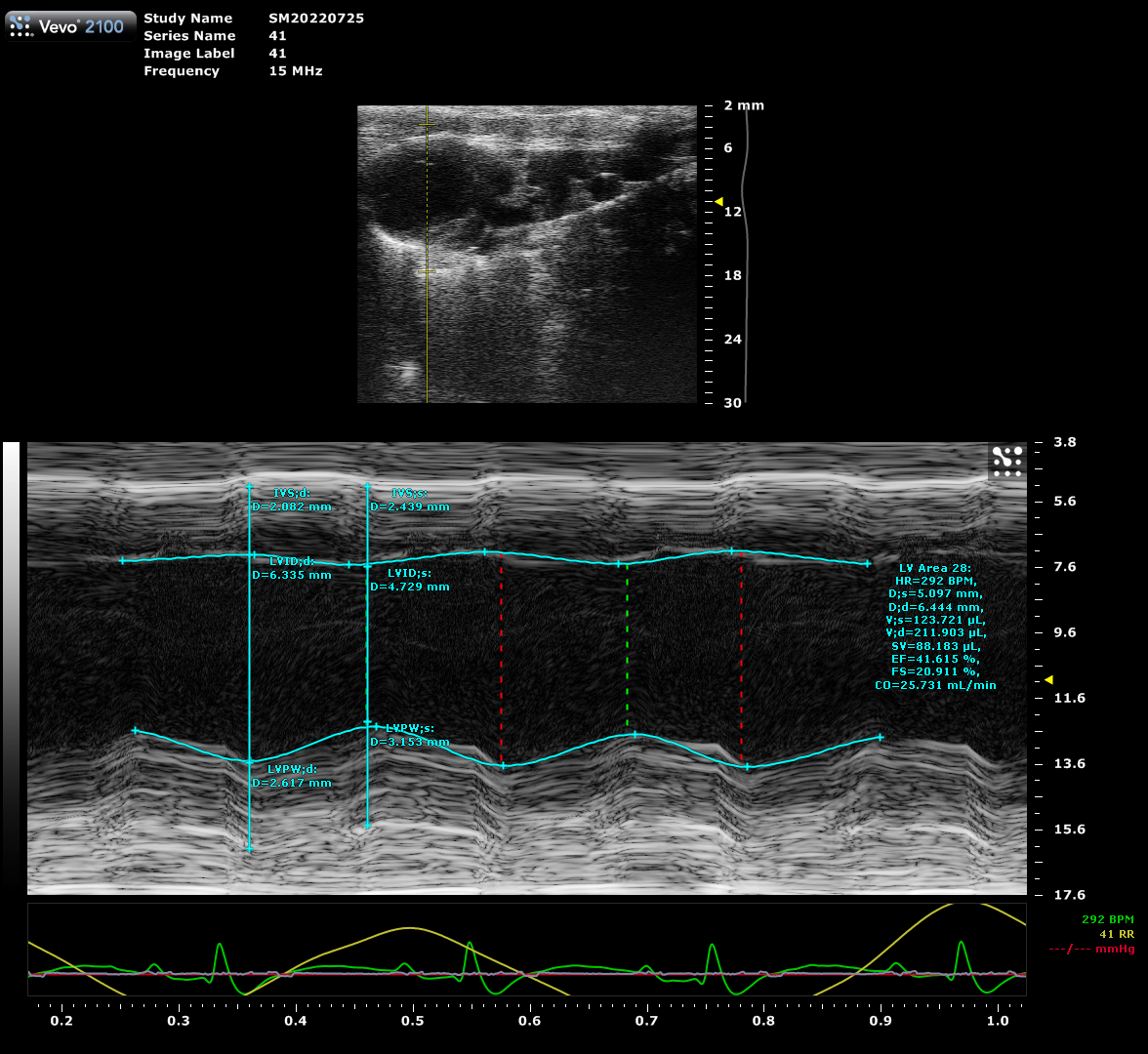

Supplement: S3 Fig — (ZIP) [file pone.0310897.s003.zip › S3 Fig/Fig3A2 Model.tif]

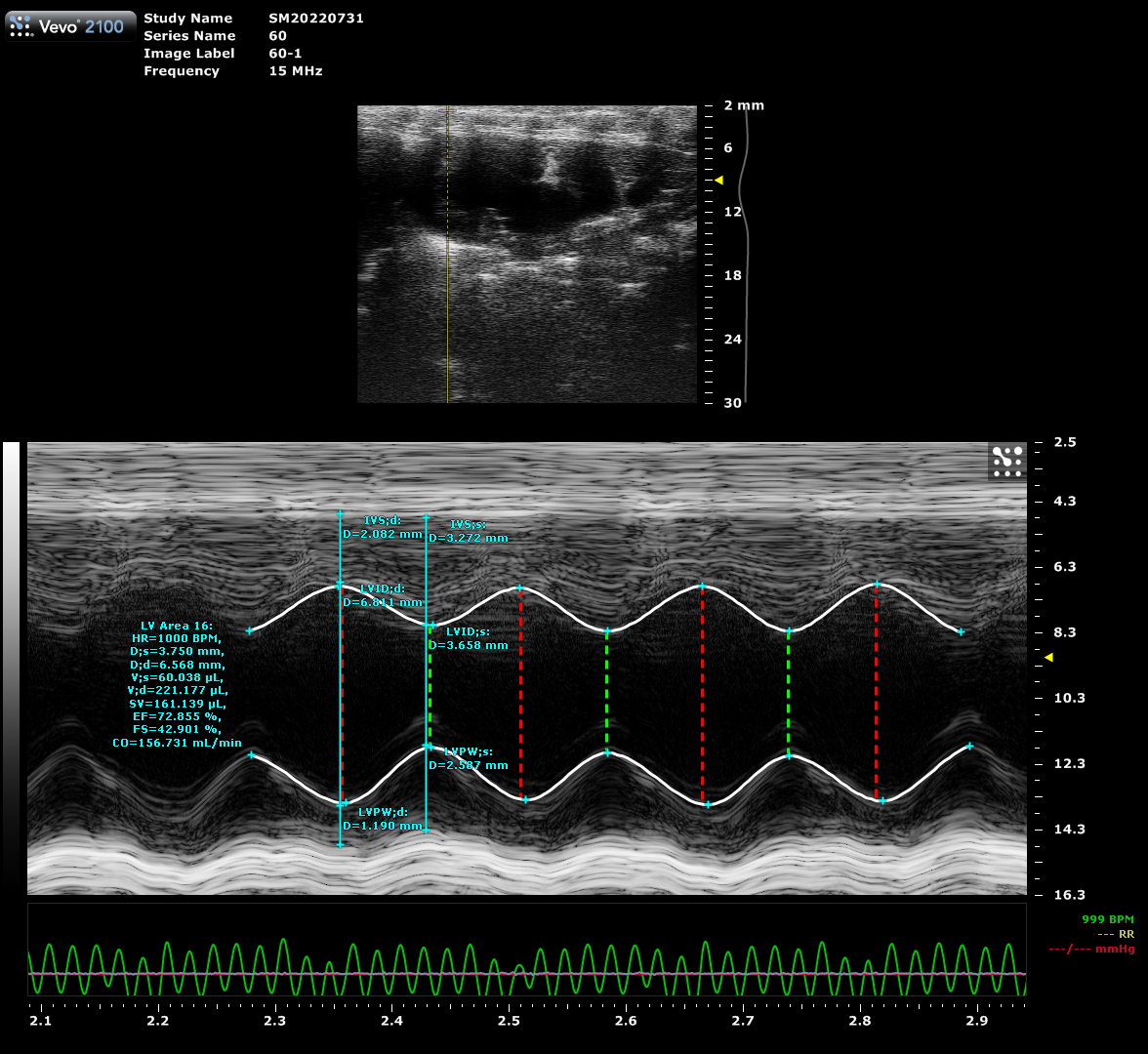

Supplement: S3 Fig — (ZIP) [file pone.0310897.s003.zip › S3 Fig/Fig3A3 QL-L.tif]

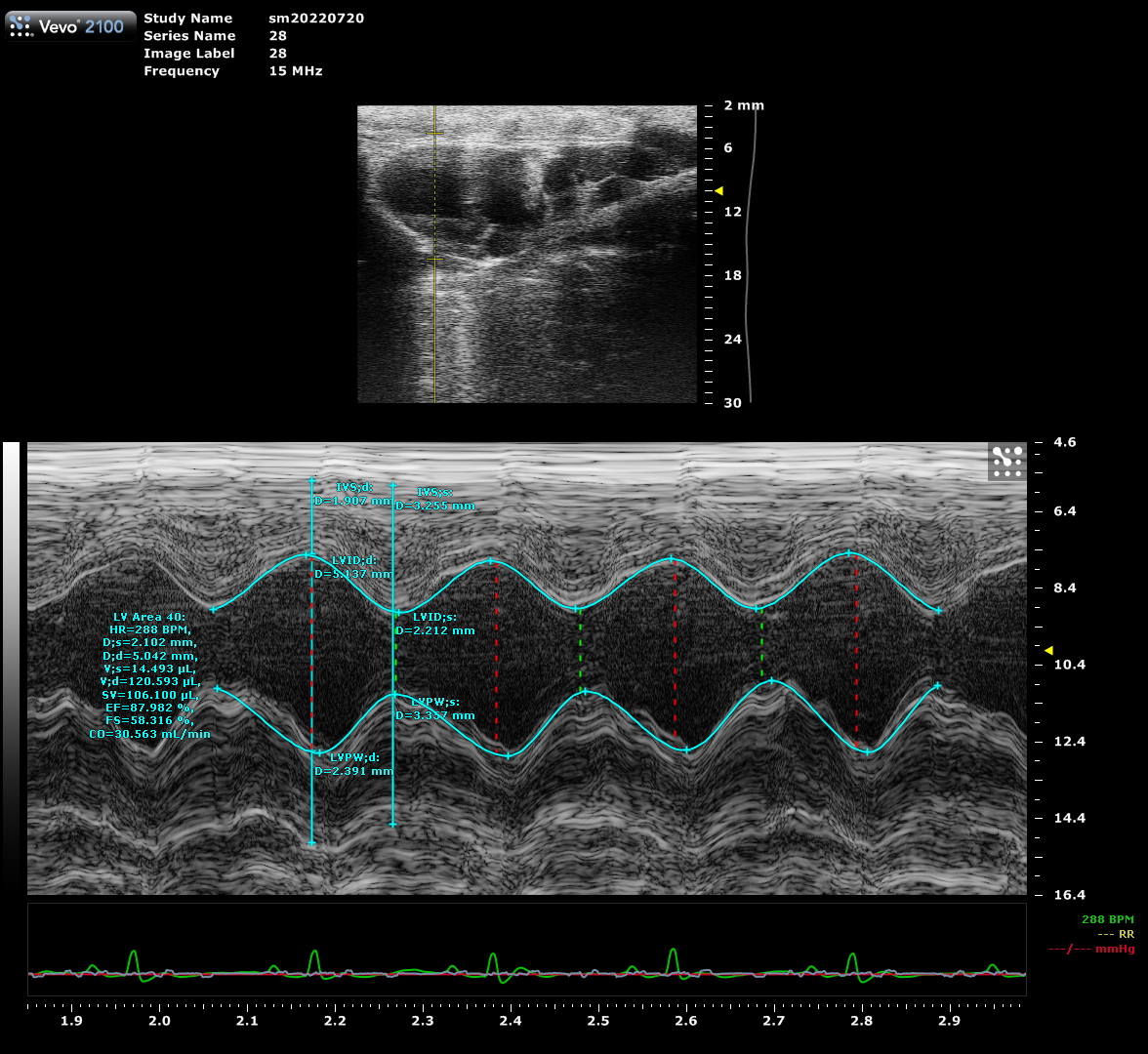

Supplement: S3 Fig — (ZIP) [file pone.0310897.s003.zip › S3 Fig/Fig3A4 QL-H.tif]

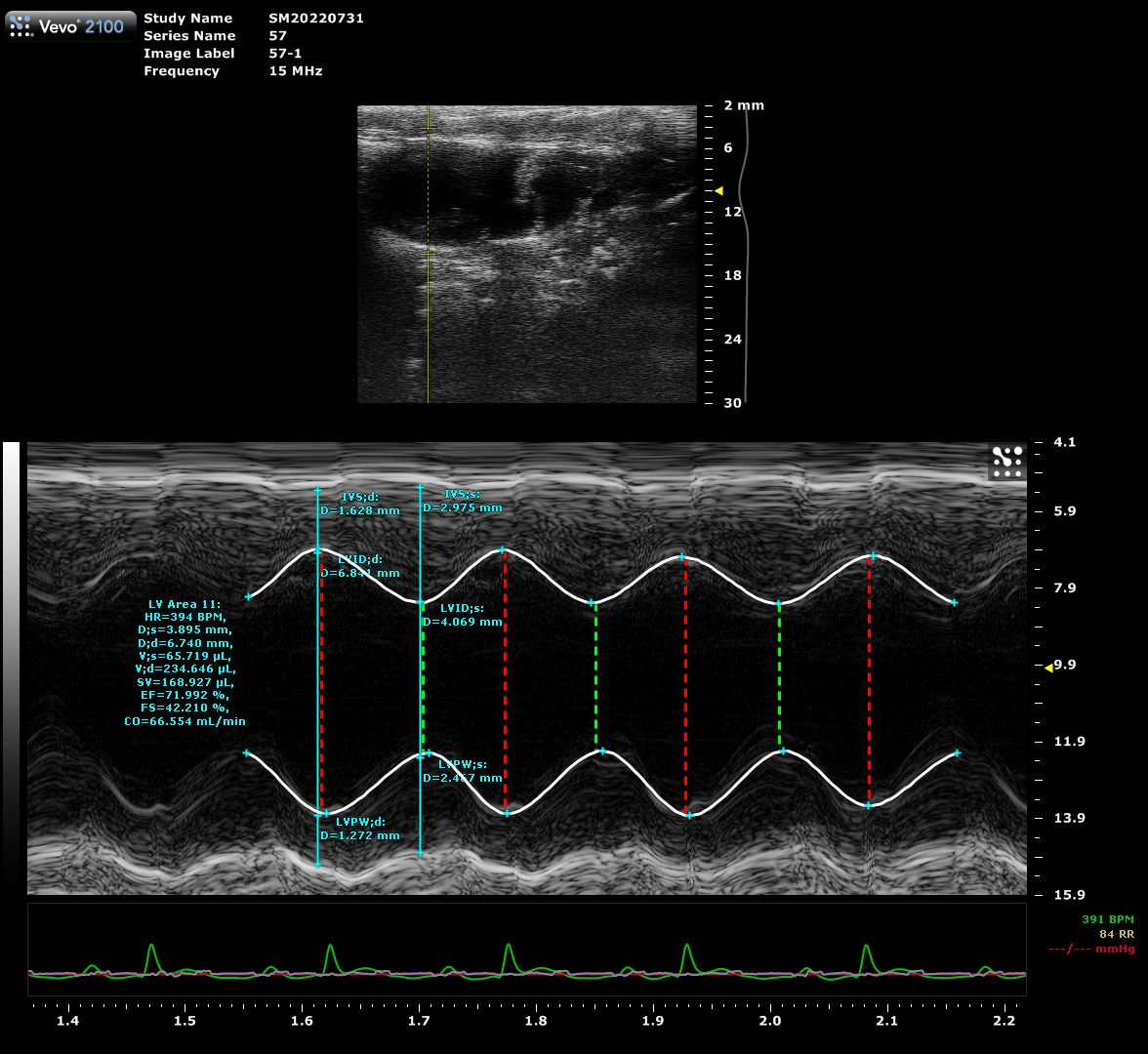

Supplement: S3 Fig — (ZIP) [file pone.0310897.s003.zip › S3 Fig/Fig3A5 Empagliflozin.tif]

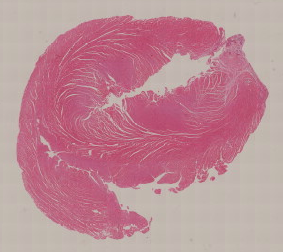

Supplement: S4 Fig — (ZIP) [file pone.0310897.s004.zip › S4 Fig/HE/Empagliflozin.tif]

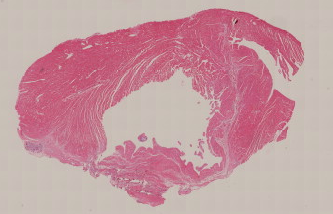

Supplement: S4 Fig — (ZIP) [file pone.0310897.s004.zip › S4 Fig/HE/Model.tif]

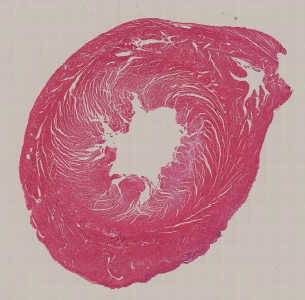

Supplement: S4 Fig — (ZIP) [file pone.0310897.s004.zip › S4 Fig/HE/QL-H.tif]

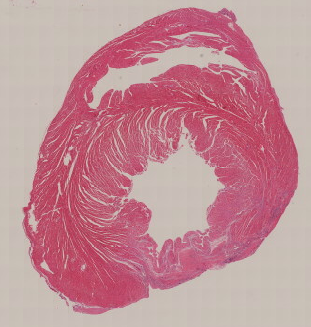

Supplement: S4 Fig — (ZIP) [file pone.0310897.s004.zip › S4 Fig/HE/QL-L.tif]

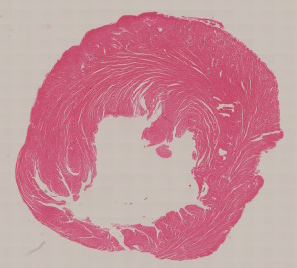

Supplement: S4 Fig — (ZIP) [file pone.0310897.s004.zip › S4 Fig/HE/Sham.tif]

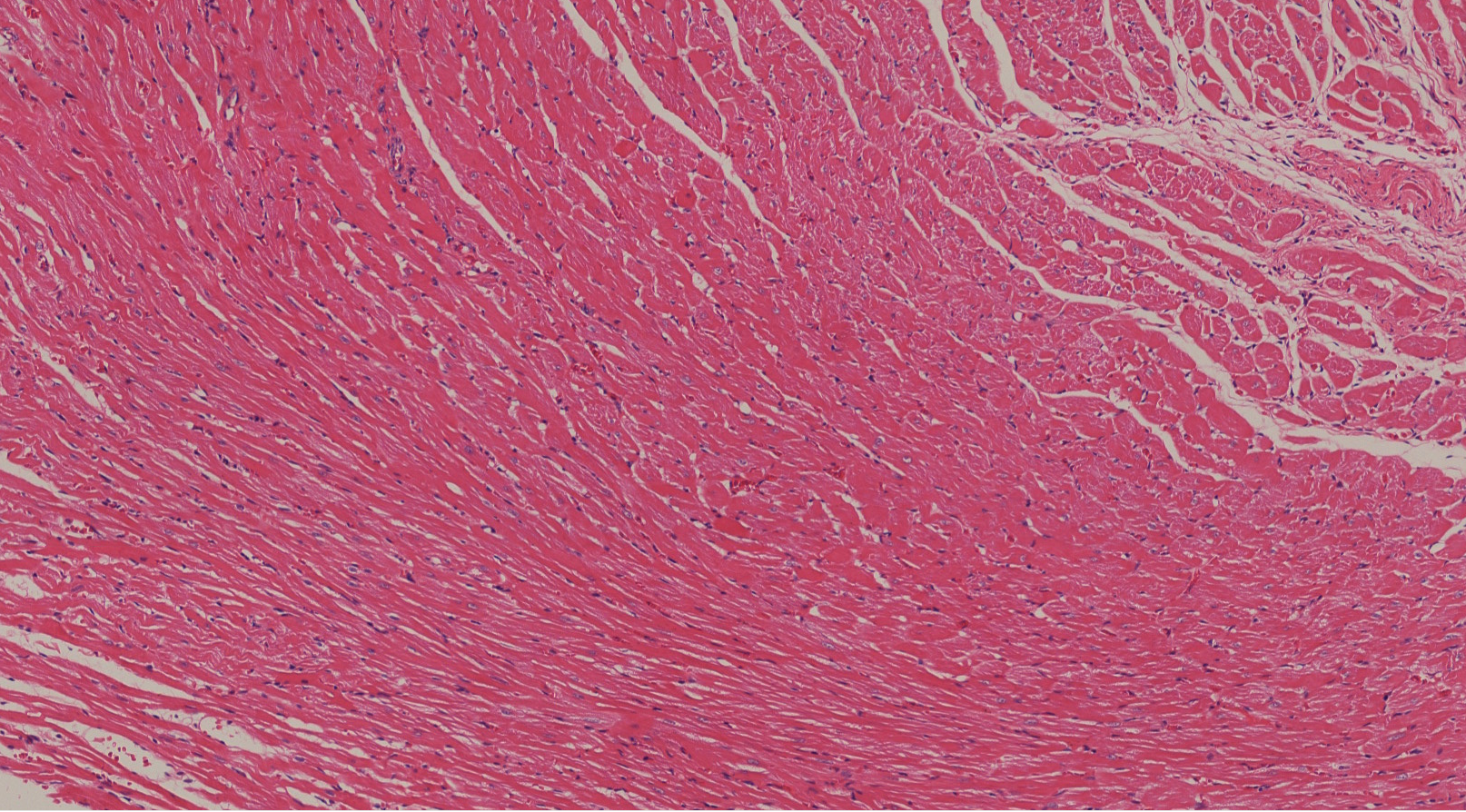

Supplement: S4 Fig — (ZIP) [file pone.0310897.s004.zip › S4 Fig/HE 10x/Empagliflozin.tif]

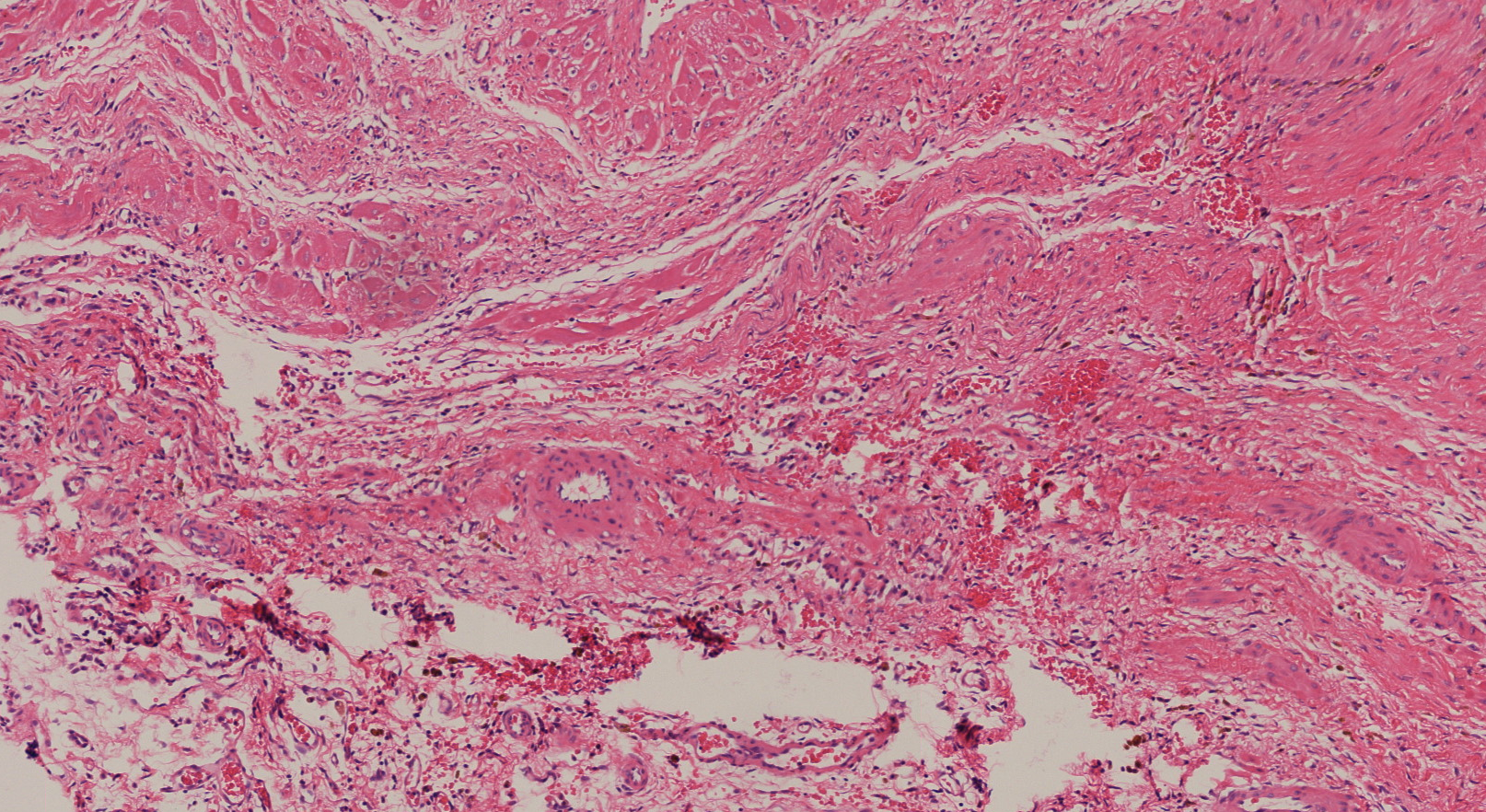

Supplement: S4 Fig — (ZIP) [file pone.0310897.s004.zip › S4 Fig/HE 10x/Model.tif]

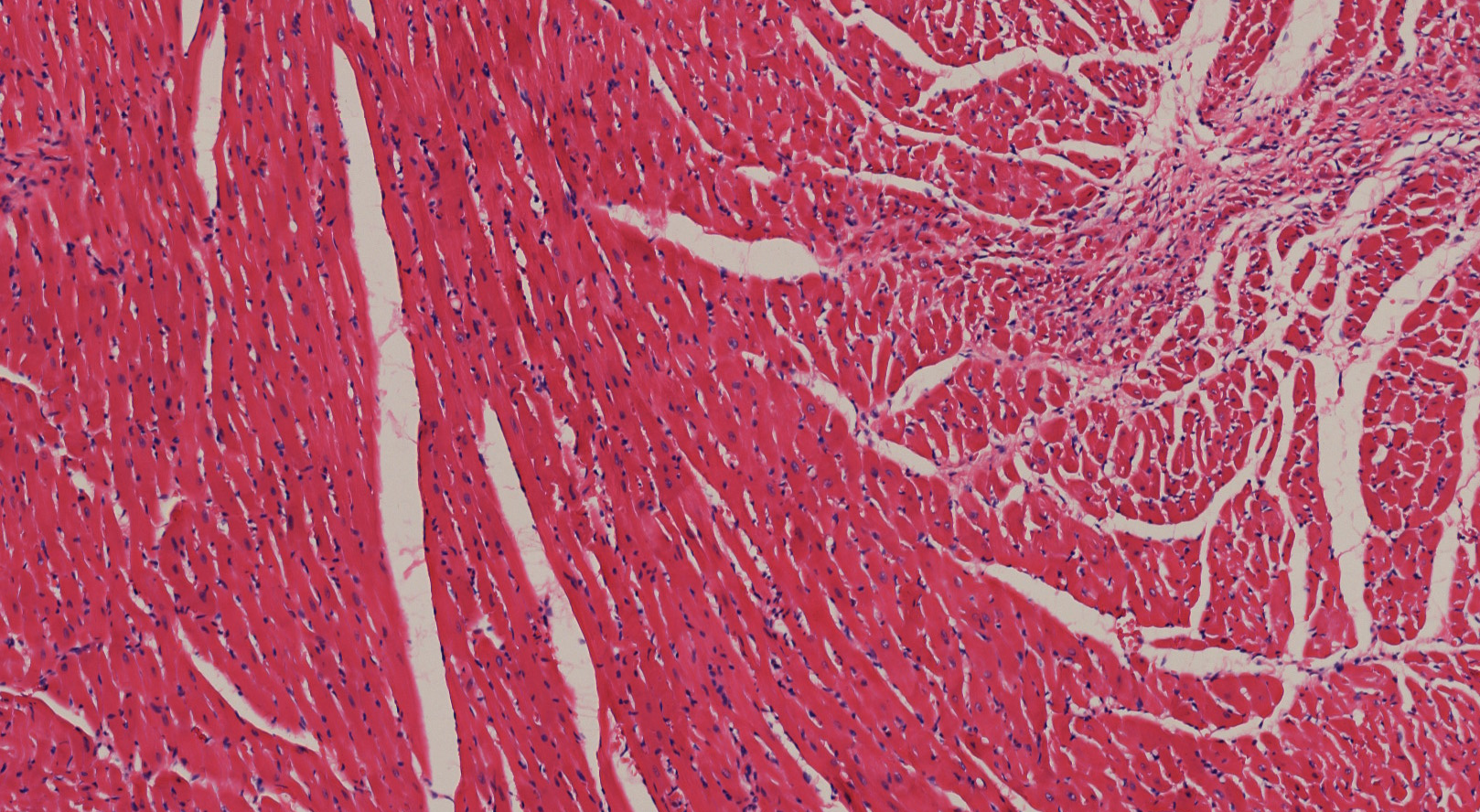

Supplement: S4 Fig — (ZIP) [file pone.0310897.s004.zip › S4 Fig/HE 10x/QL-H.tif]

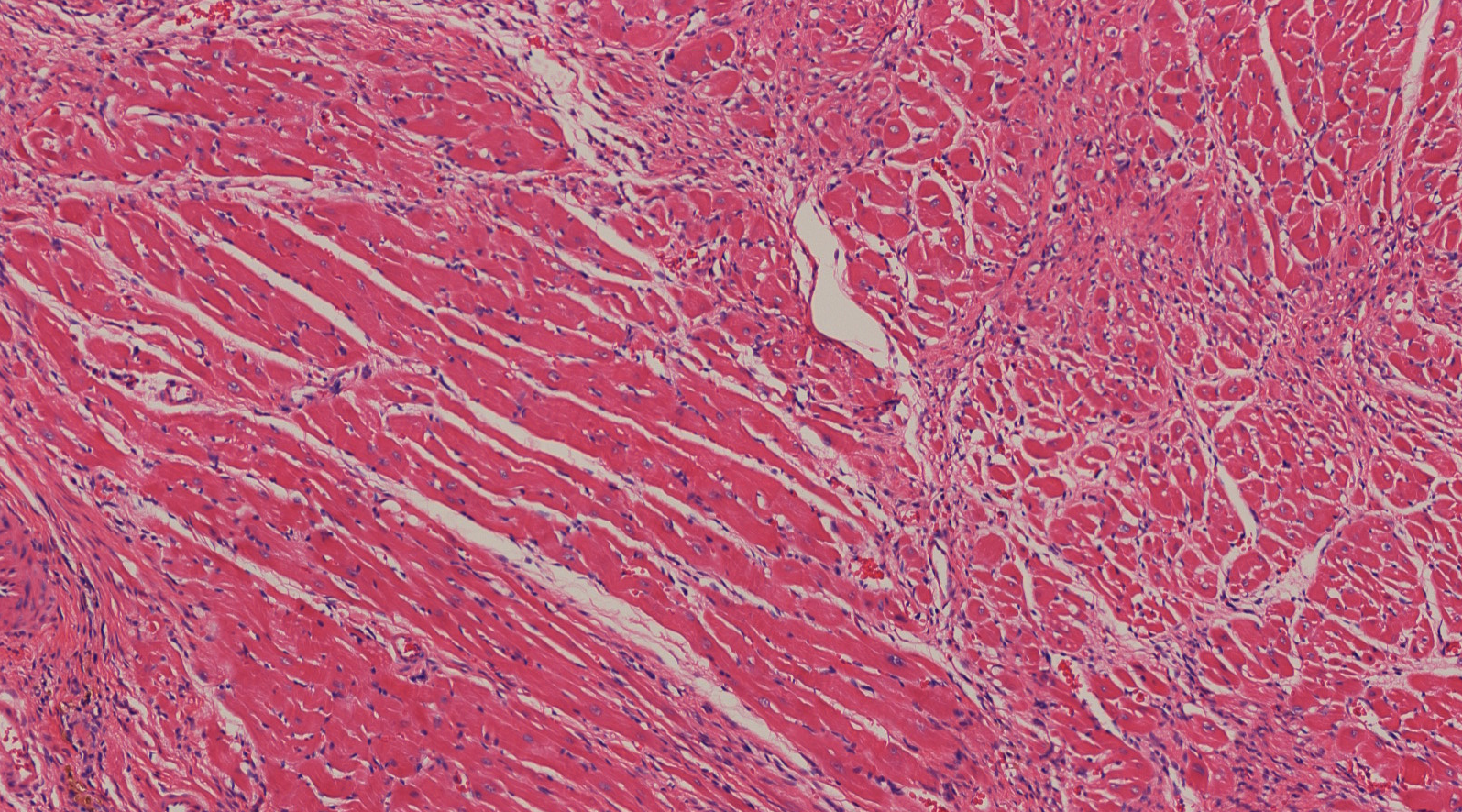

Supplement: S4 Fig — (ZIP) [file pone.0310897.s004.zip › S4 Fig/HE 10x/QL-L.tif]

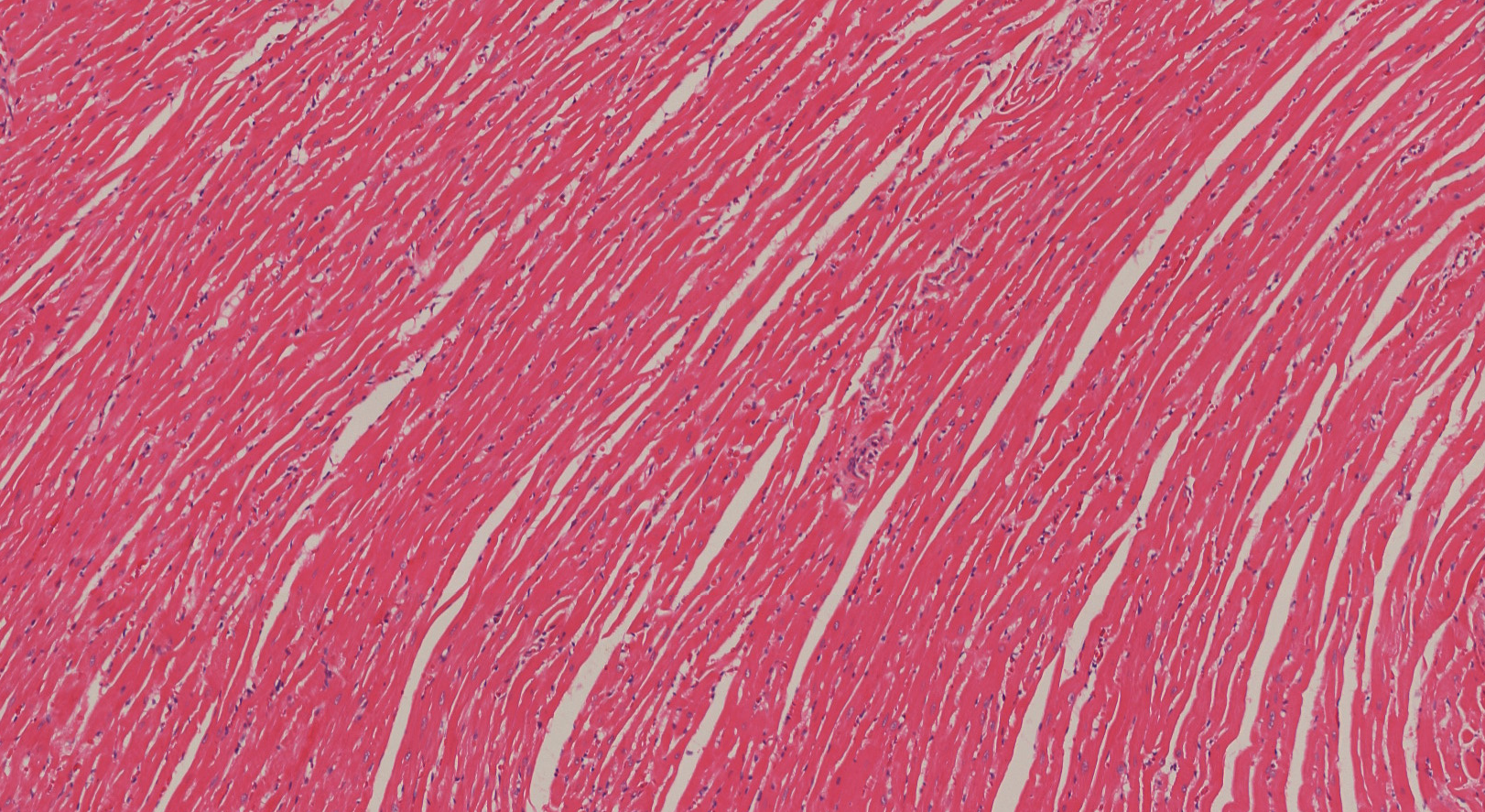

Supplement: S4 Fig — (ZIP) [file pone.0310897.s004.zip › S4 Fig/HE 10x/Sham.tif]

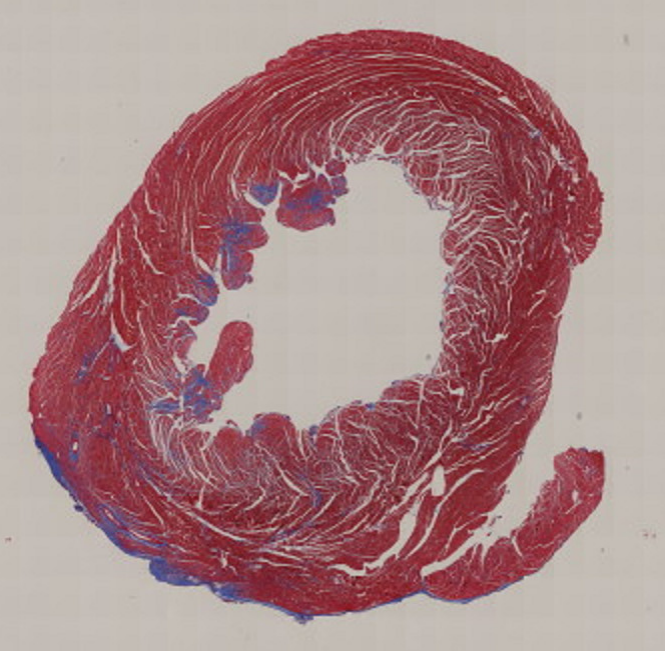

Supplement: S5 Fig — (ZIP) [file pone.0310897.s005.zip › S5 Fig/Masson/Empagliflozin.tif]

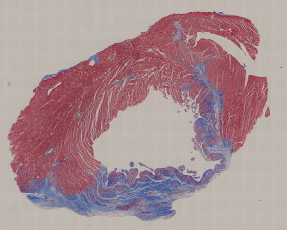

Supplement: S5 Fig — (ZIP) [file pone.0310897.s005.zip › S5 Fig/Masson/Model.tif]

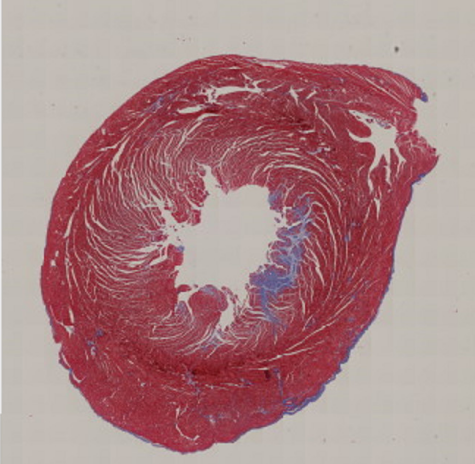

Supplement: S5 Fig — (ZIP) [file pone.0310897.s005.zip › S5 Fig/Masson/QL-H.tif]

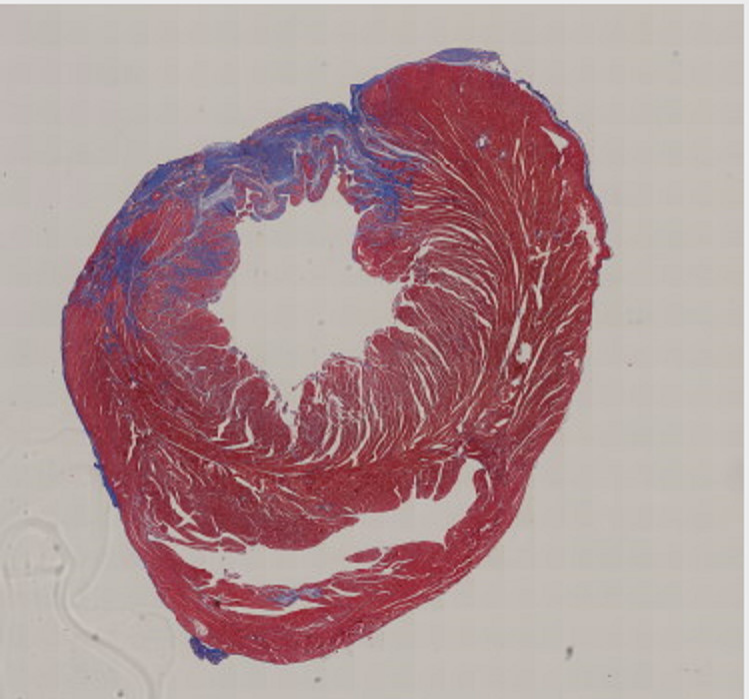

Supplement: S5 Fig — (ZIP) [file pone.0310897.s005.zip › S5 Fig/Masson/QL-L.tif]

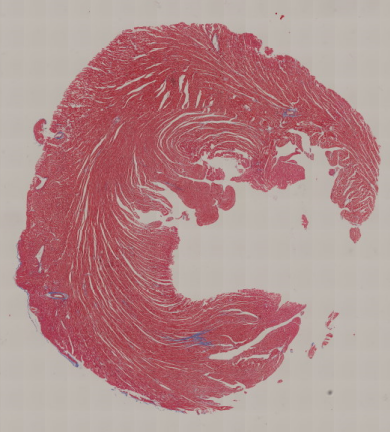

Supplement: S5 Fig — (ZIP) [file pone.0310897.s005.zip › S5 Fig/Masson/Sham.tif]

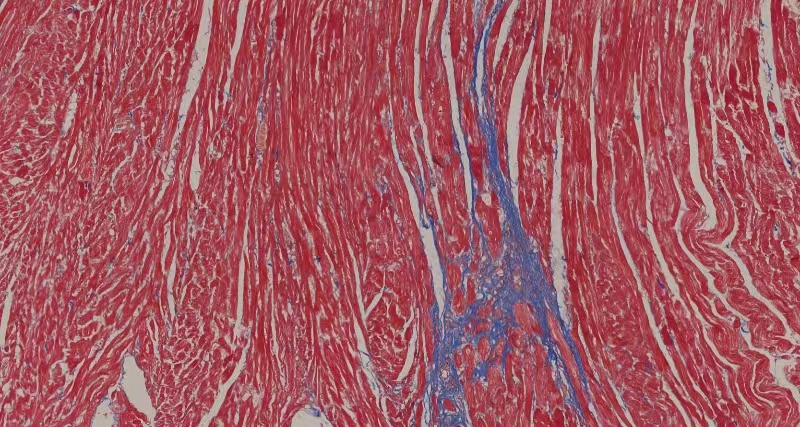

Supplement: S5 Fig — (ZIP) [file pone.0310897.s005.zip › S5 Fig/Masson10x/27假.jpg]

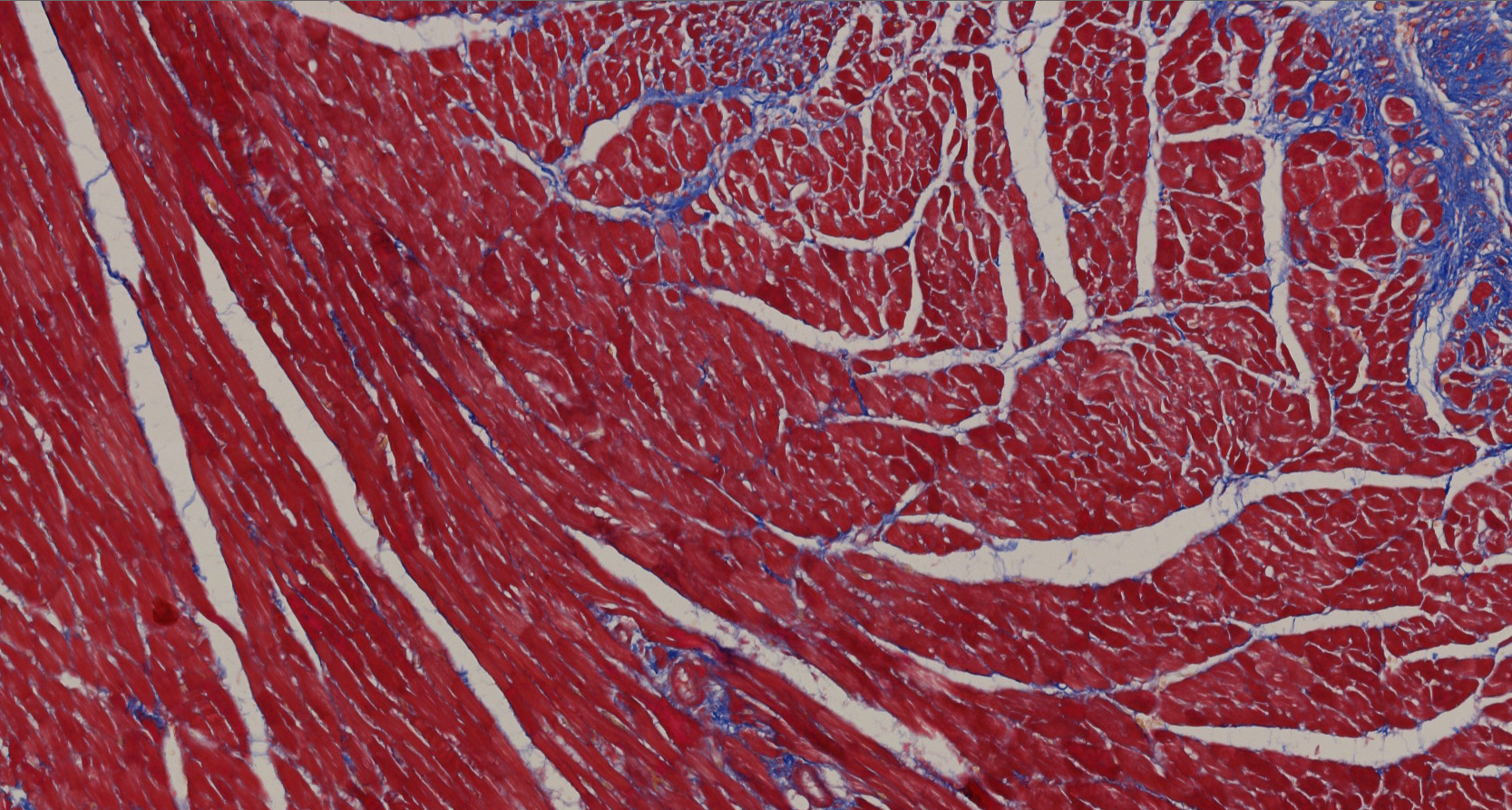

Supplement: S5 Fig — (ZIP) [file pone.0310897.s005.zip › S5 Fig/Masson10x/55高.png]

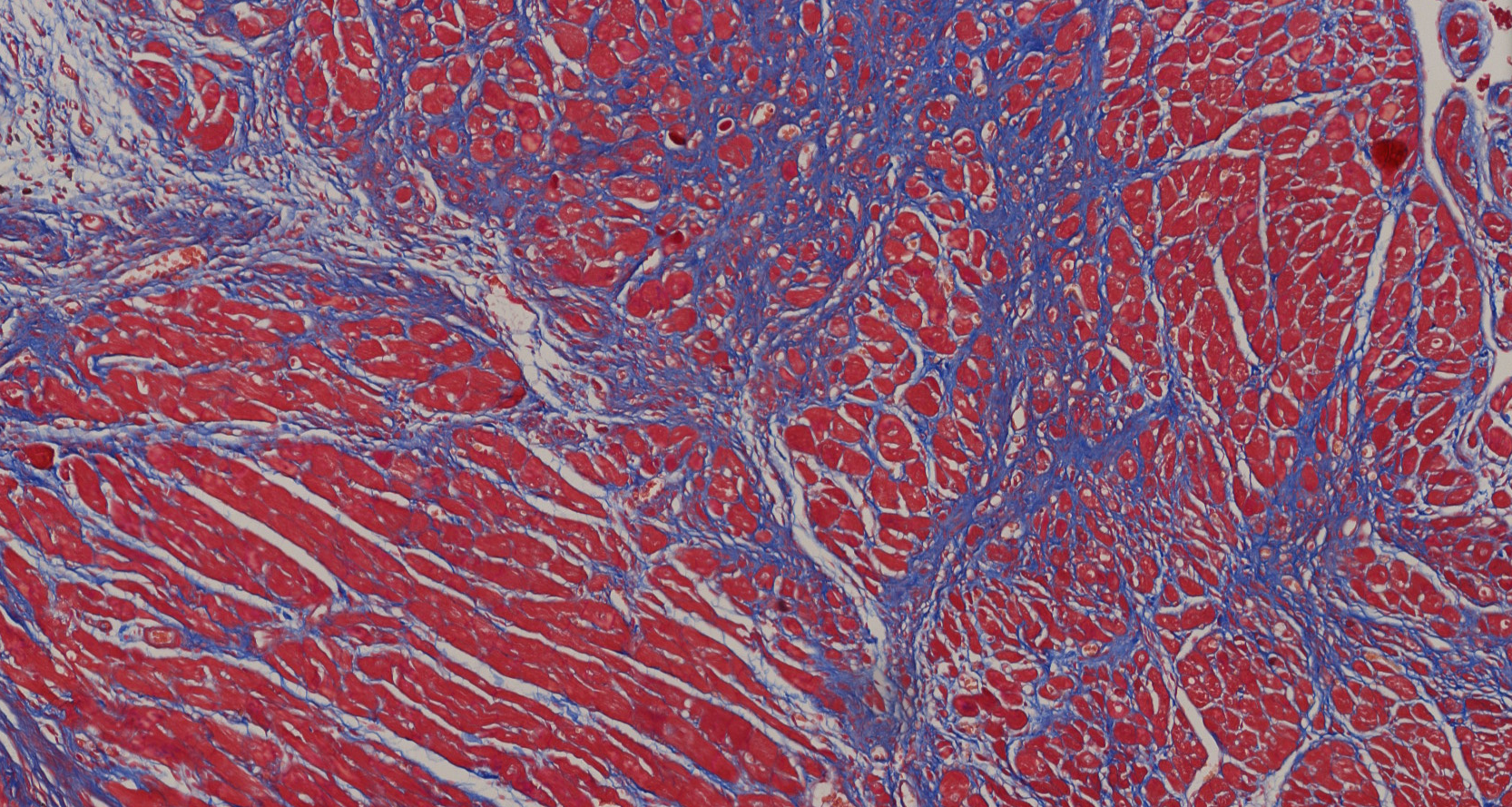

Supplement: S5 Fig — (ZIP) [file pone.0310897.s005.zip › S5 Fig/Masson10x/56低.png]

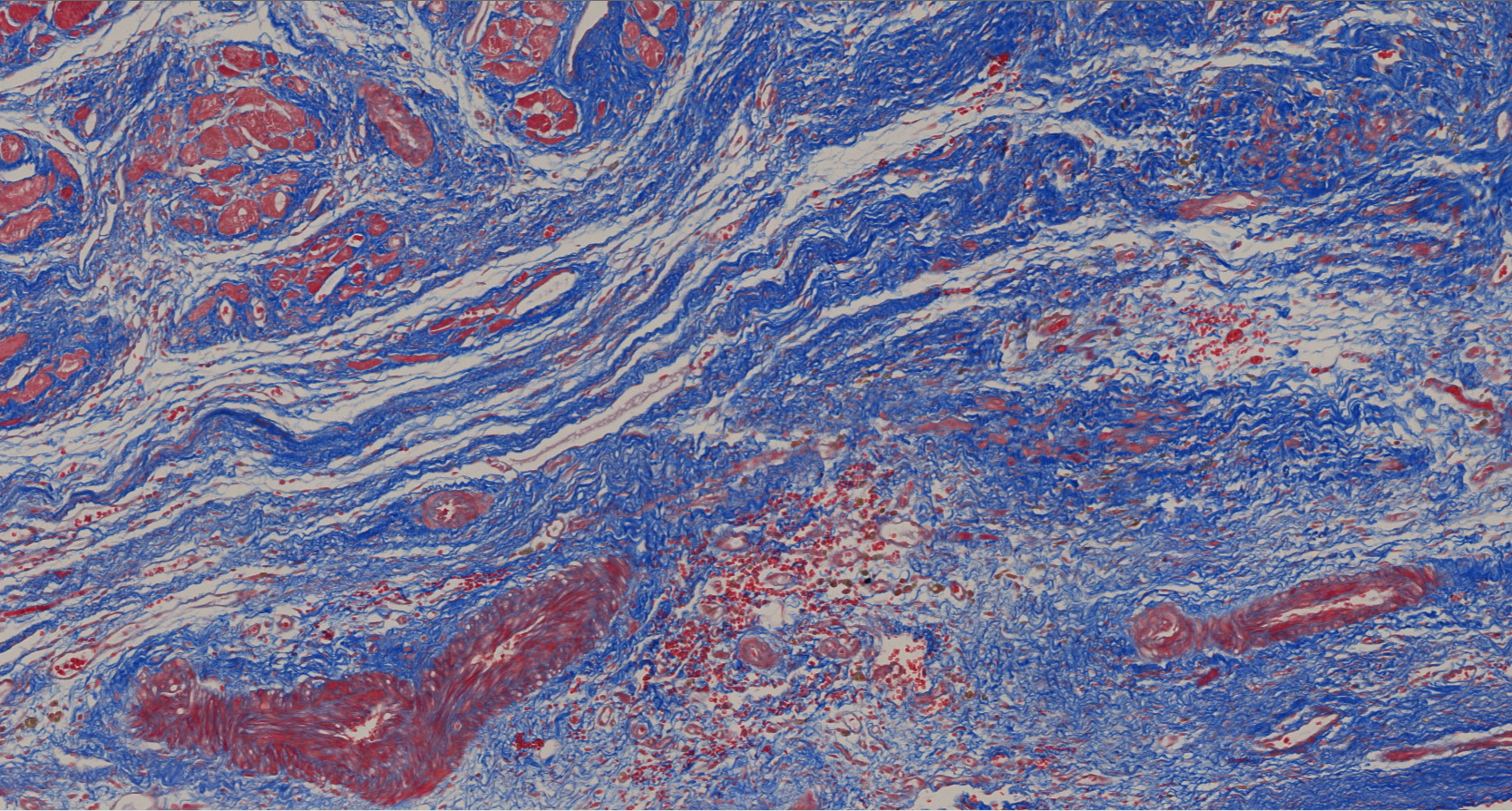

Supplement: S5 Fig — (ZIP) [file pone.0310897.s005.zip › S5 Fig/Masson10x/模11.png]

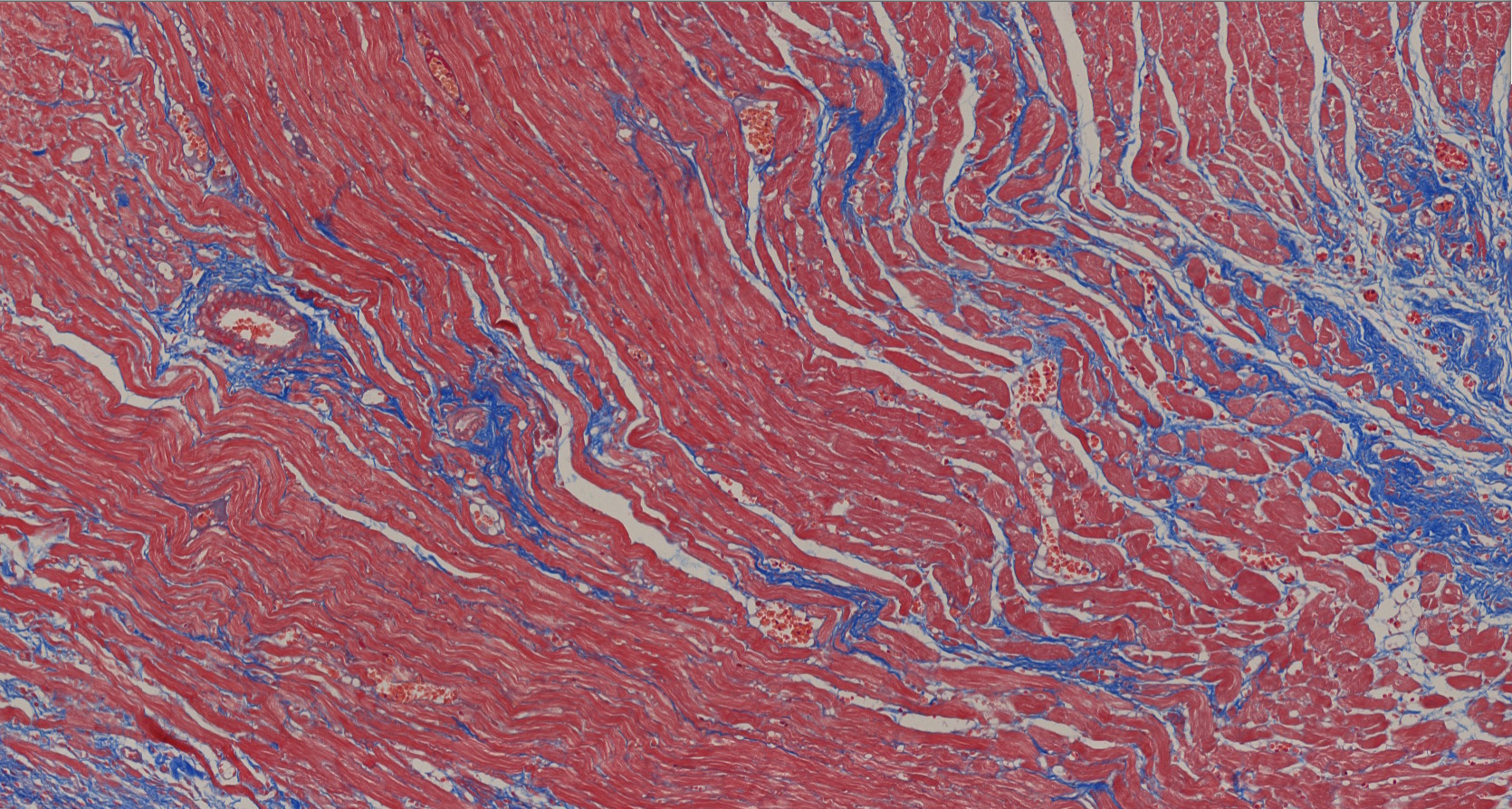

Supplement: S5 Fig — (ZIP) [file pone.0310897.s005.zip › S5 Fig/Masson10x/阳18.png]

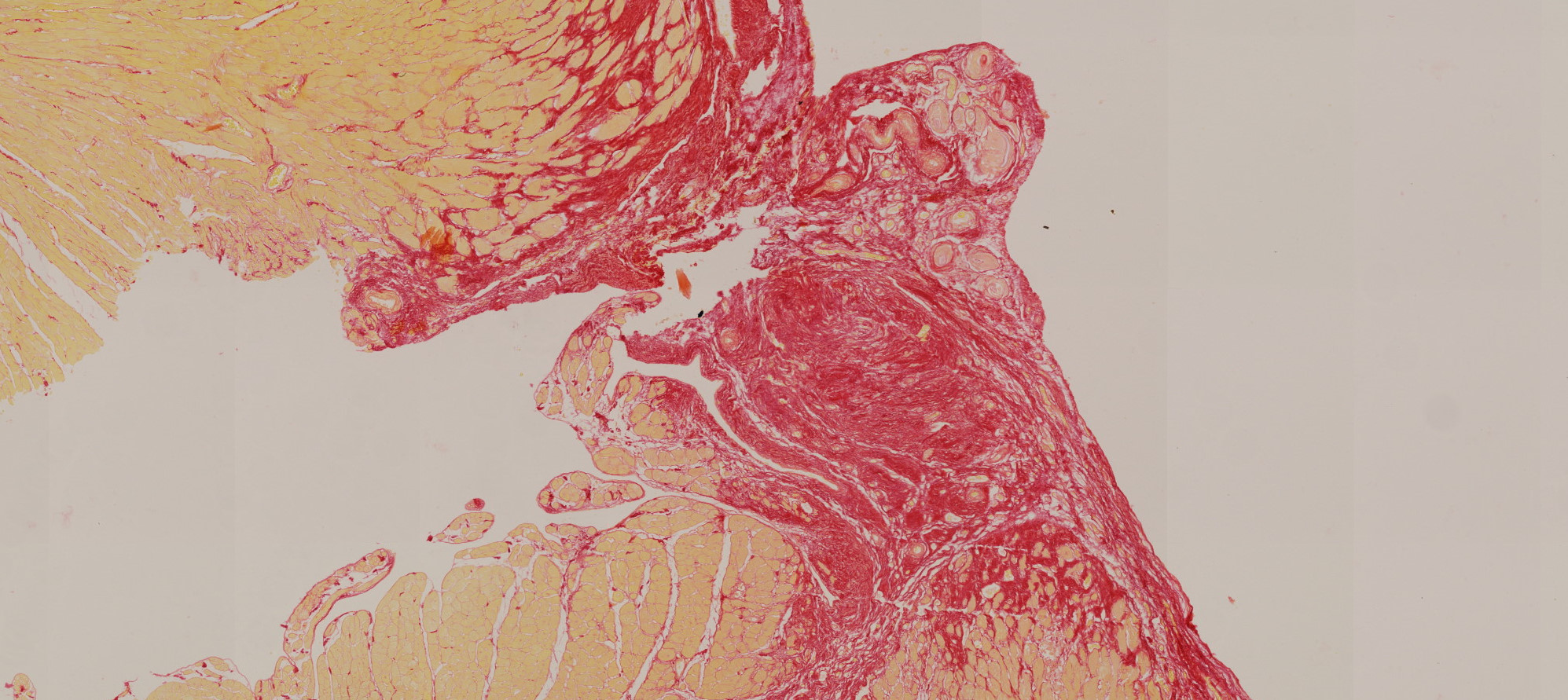

Supplement: S6 Fig — (ZIP) [file pone.0310897.s006.zip › S6 Fig/PSR 10X under optical microscope/Empagliflozin.tif]

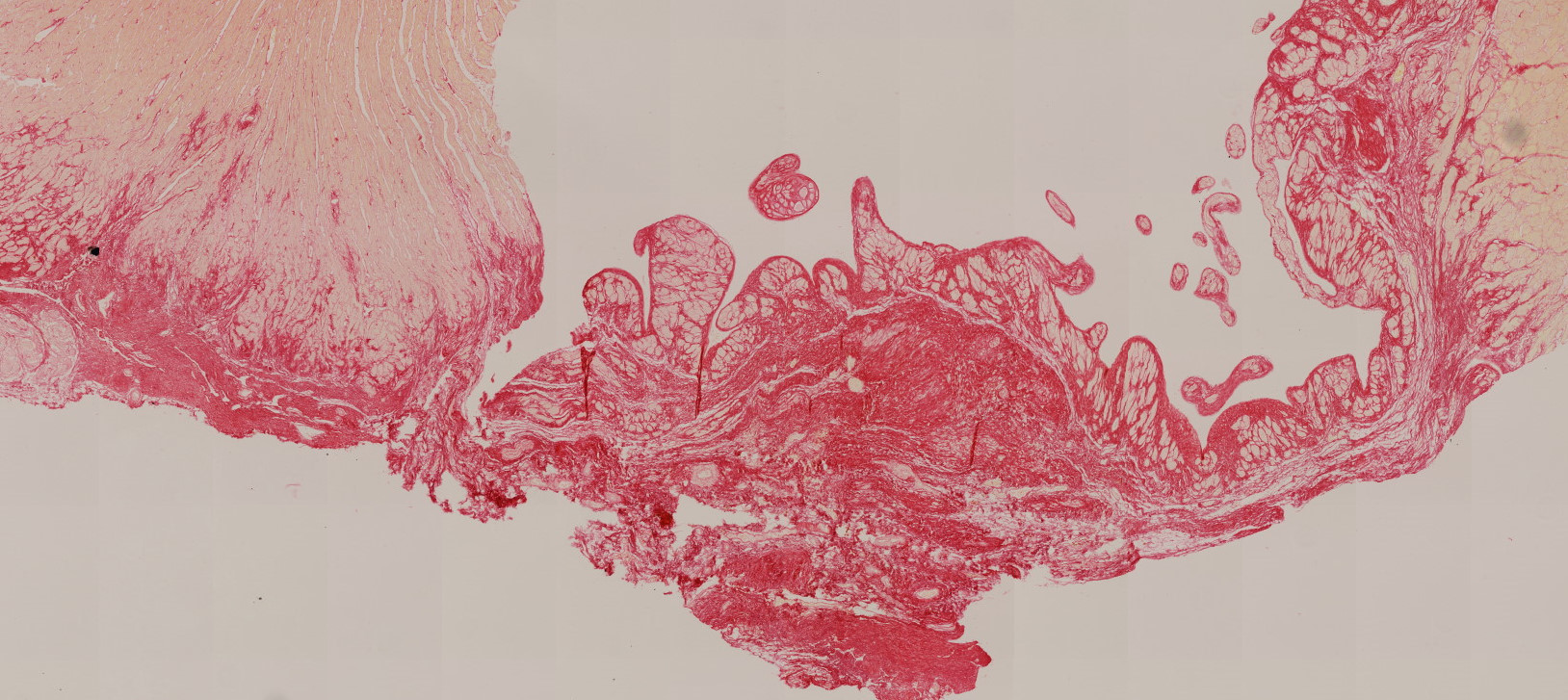

Supplement: S6 Fig — (ZIP) [file pone.0310897.s006.zip › S6 Fig/PSR 10X under optical microscope/Model.tif]

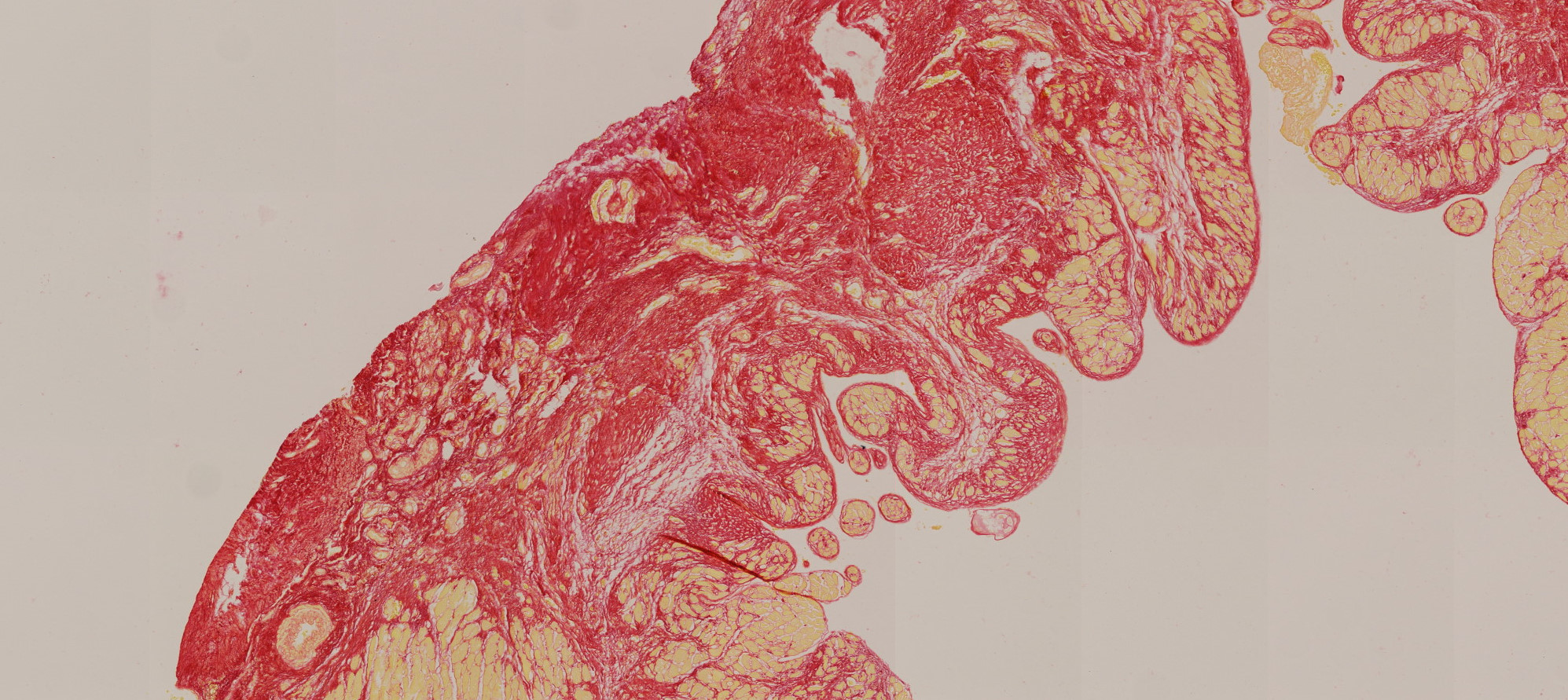

Supplement: S6 Fig — (ZIP) [file pone.0310897.s006.zip › S6 Fig/PSR 10X under optical microscope/QL-L.tif]

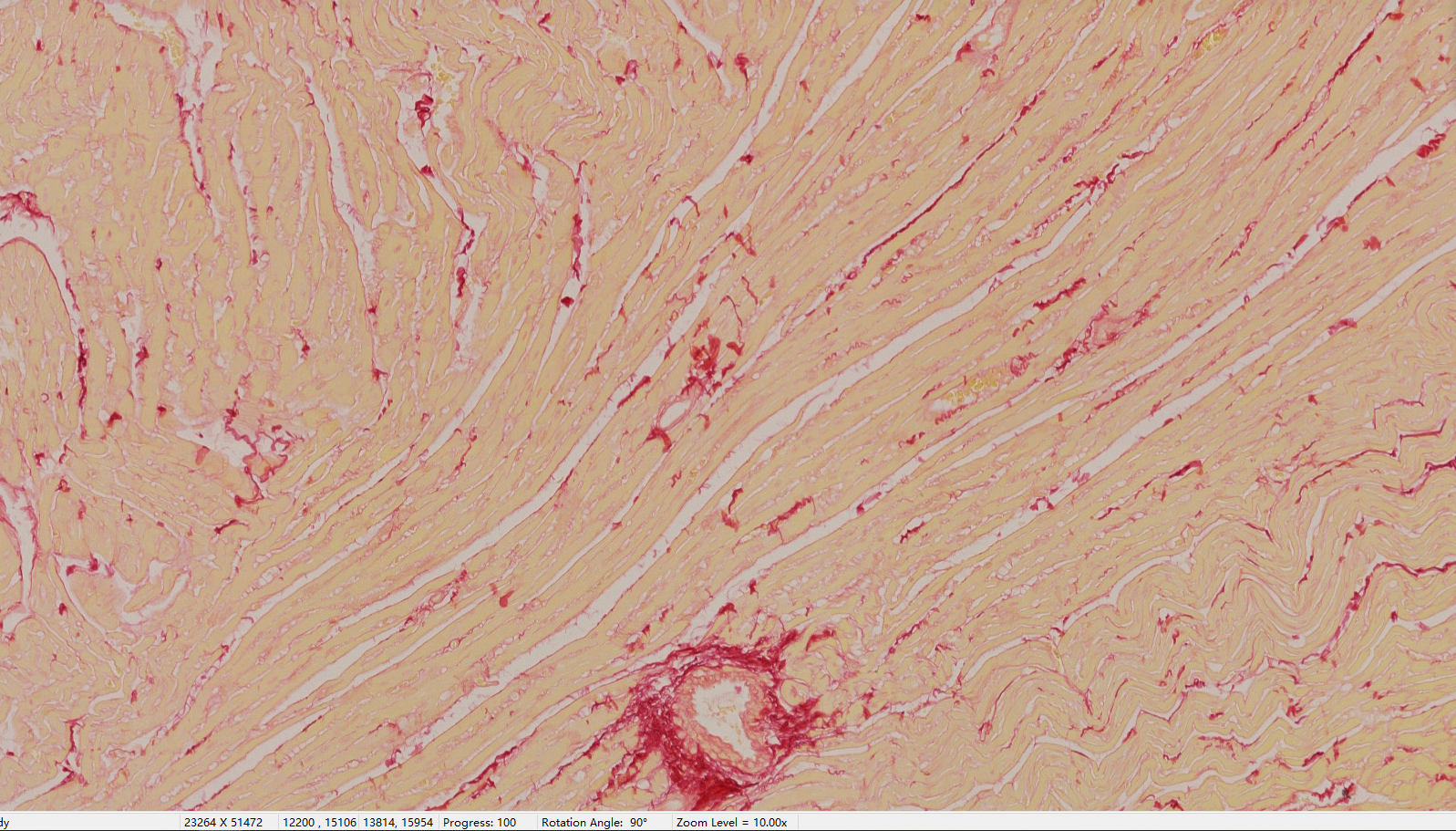

Supplement: S6 Fig — (ZIP) [file pone.0310897.s006.zip › S6 Fig/PSR 10X under optical microscope/Sham.tif]

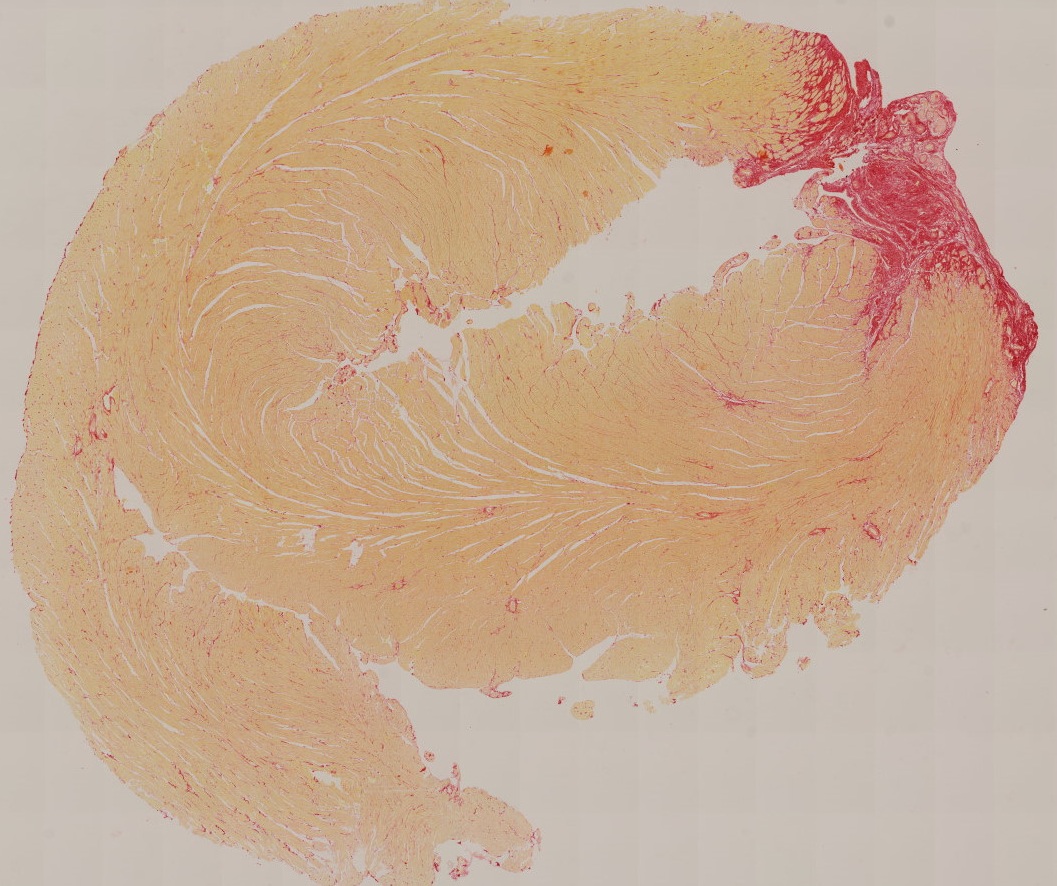

Supplement: S6 Fig — (ZIP) [file pone.0310897.s006.zip › S6 Fig/PSR under optical microscope/Empagliflozin.tif]

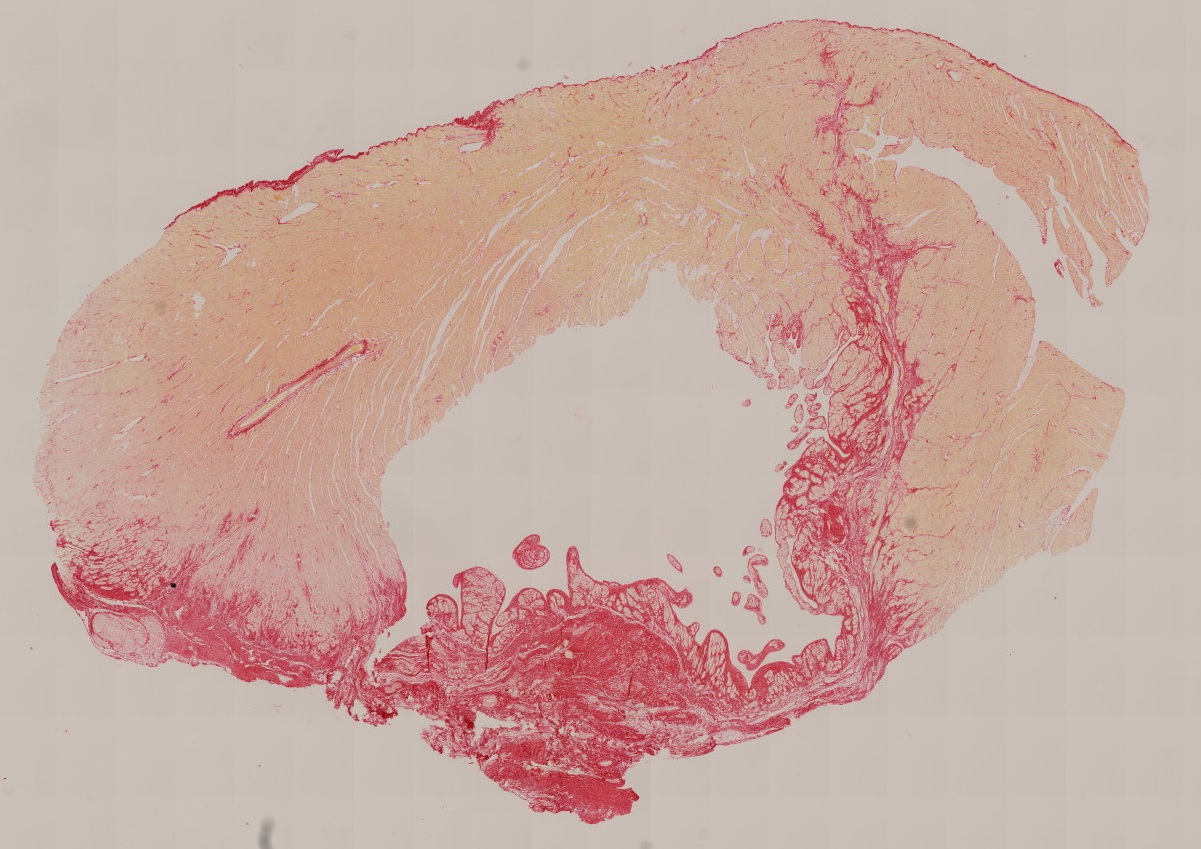

Supplement: S6 Fig — (ZIP) [file pone.0310897.s006.zip › S6 Fig/PSR under optical microscope/Model.tif]

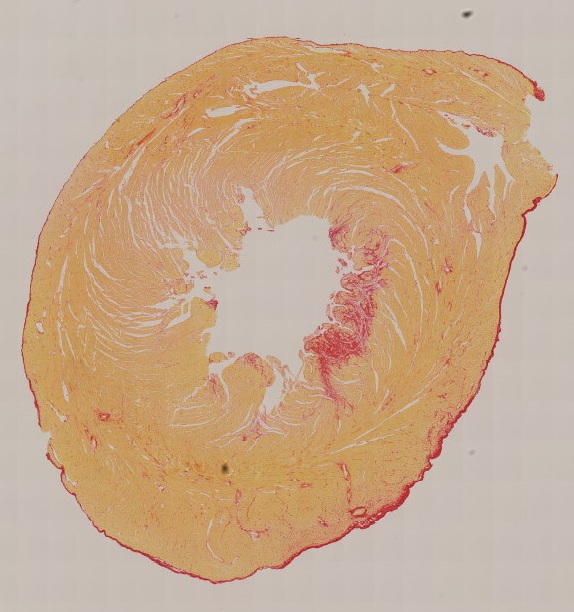

Supplement: S6 Fig — (ZIP) [file pone.0310897.s006.zip › S6 Fig/PSR under optical microscope/QL-H.tif]

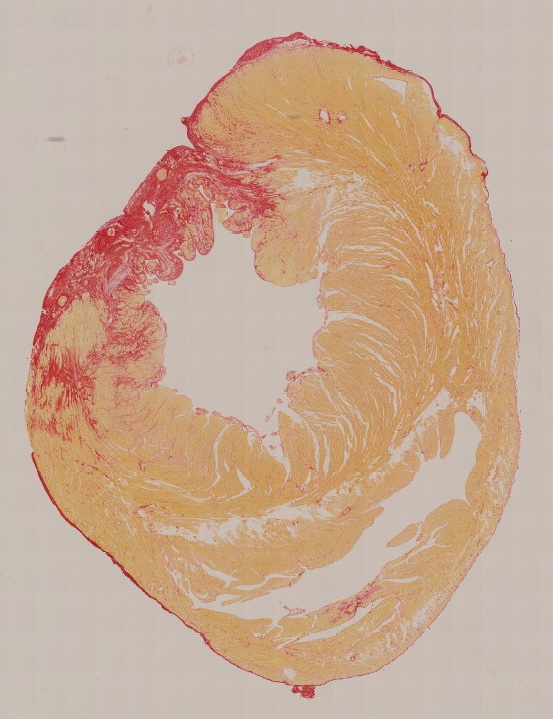

Supplement: S6 Fig — (ZIP) [file pone.0310897.s006.zip › S6 Fig/PSR under optical microscope/QL-L.tif]

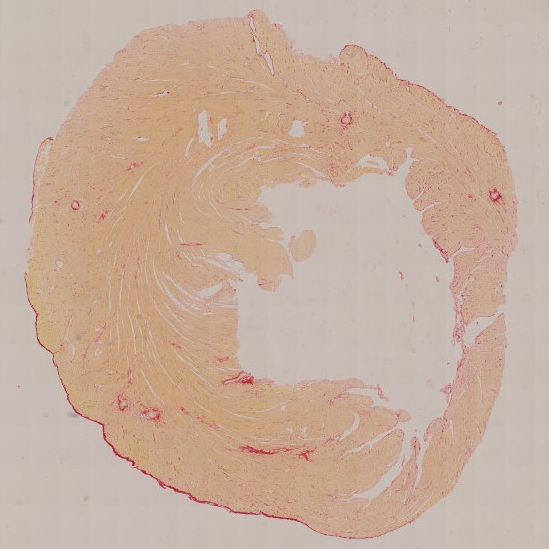

Supplement: S6 Fig — (ZIP) [file pone.0310897.s006.zip › S6 Fig/PSR under optical microscope/Sham.tif]

## PSR 10X

Sham

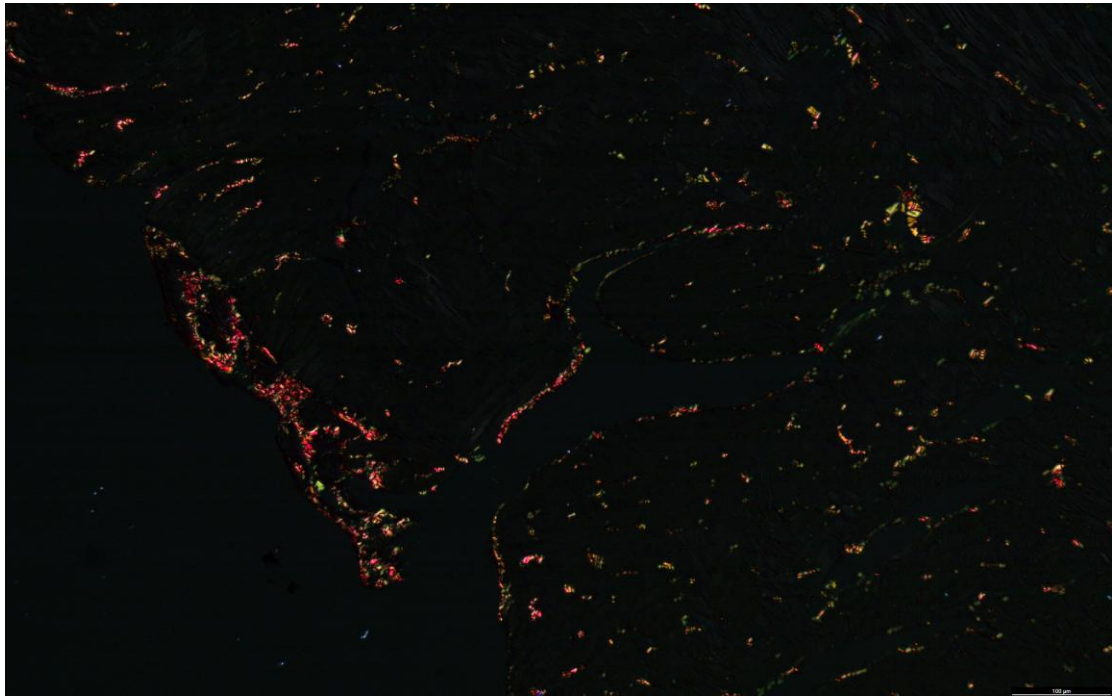

Model

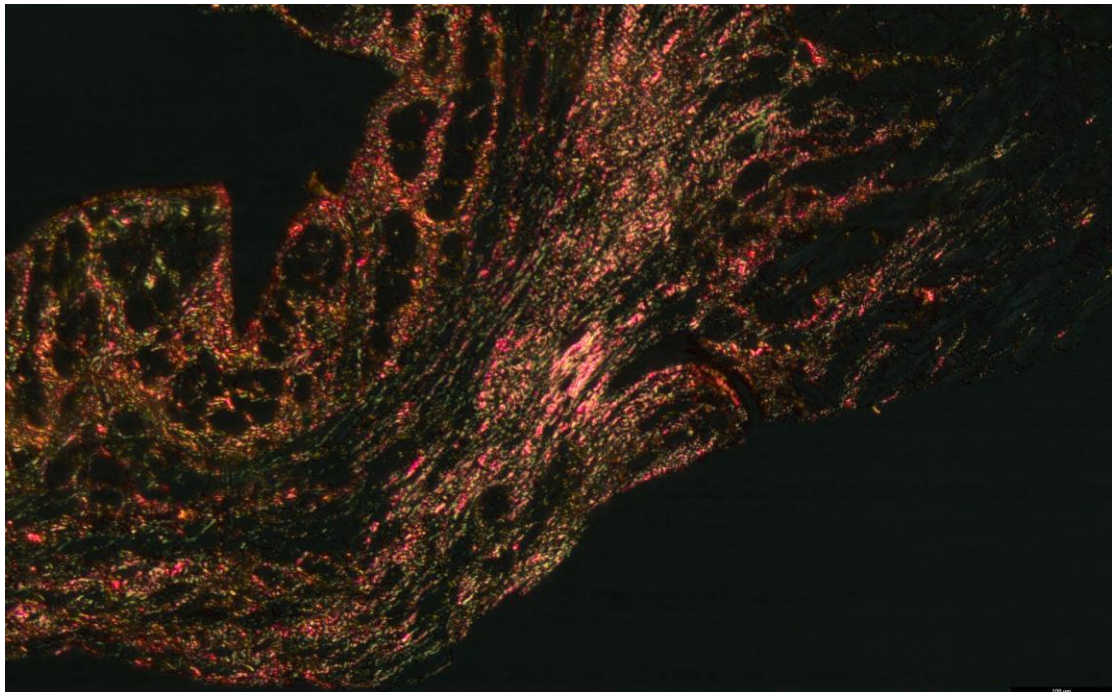

QL-L

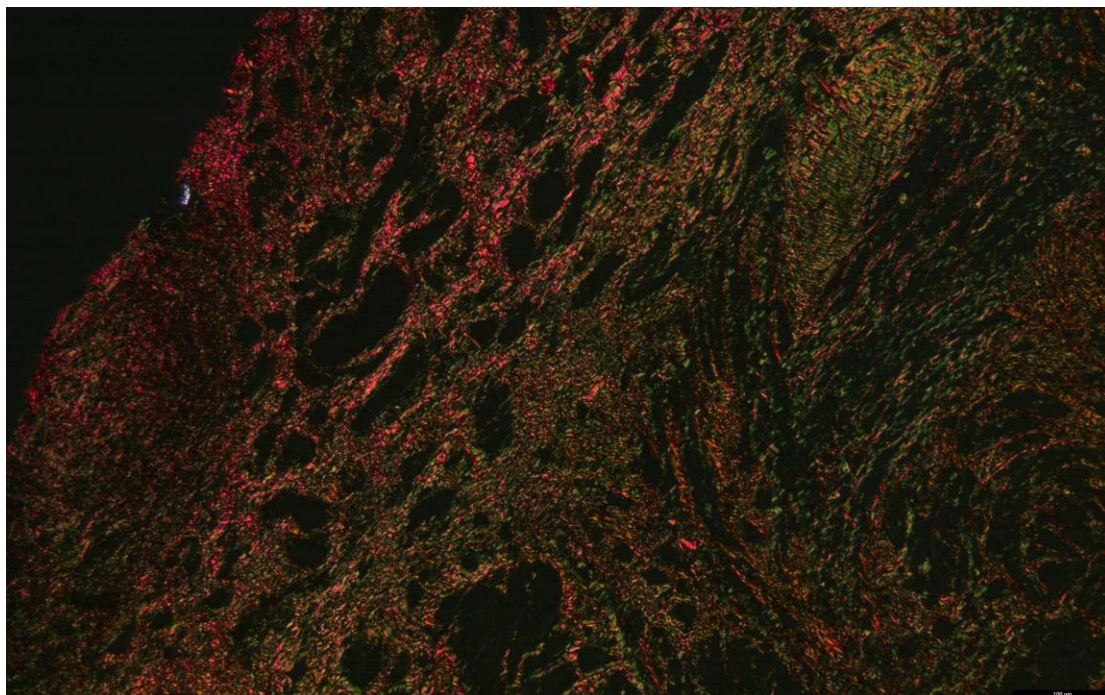

QL-H

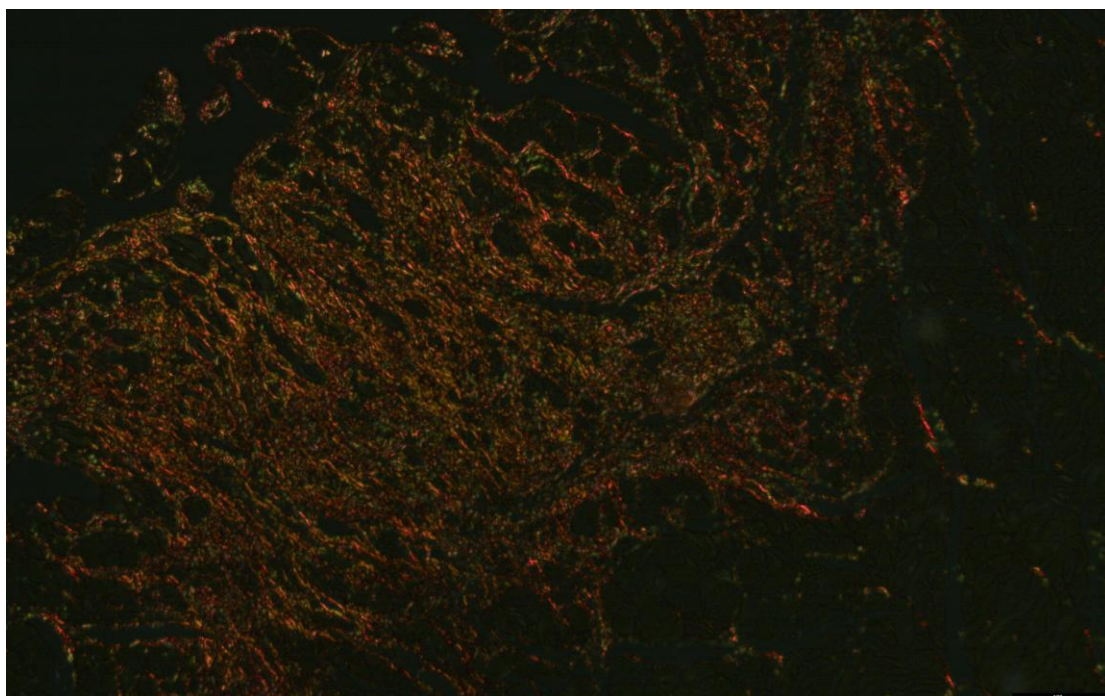

Empagliflozin

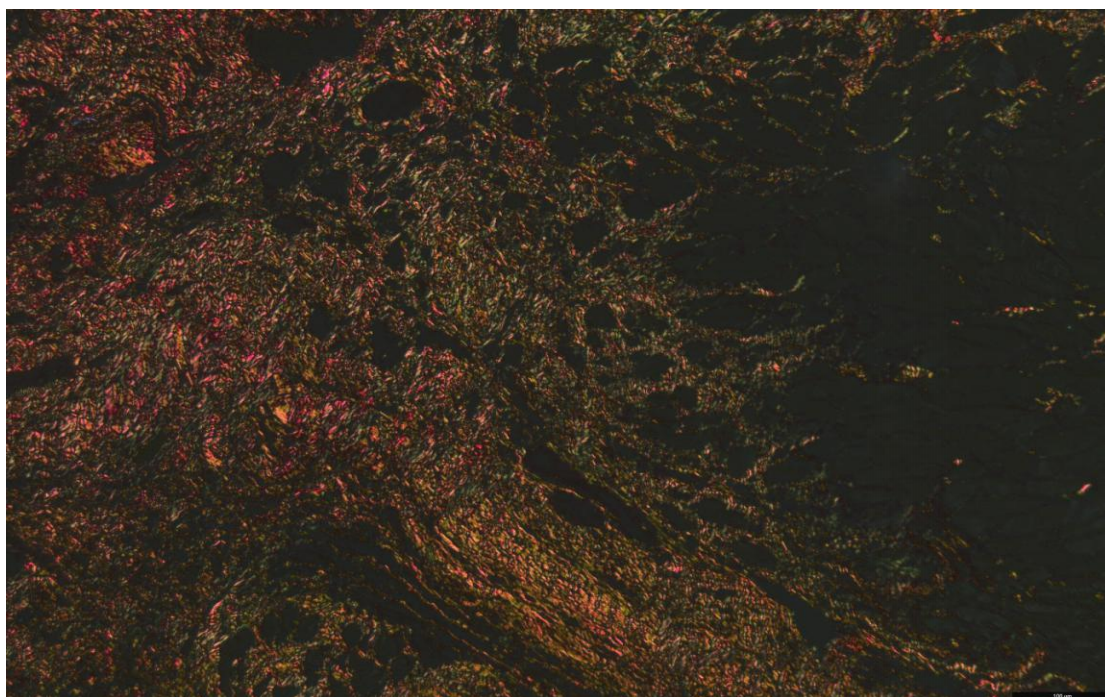

Supplement: S7 Fig — (ZIP) [file pone.0310897.s007.zip › S7 Fig/PSR 10X.pdf]

## PSR 20X

Sham

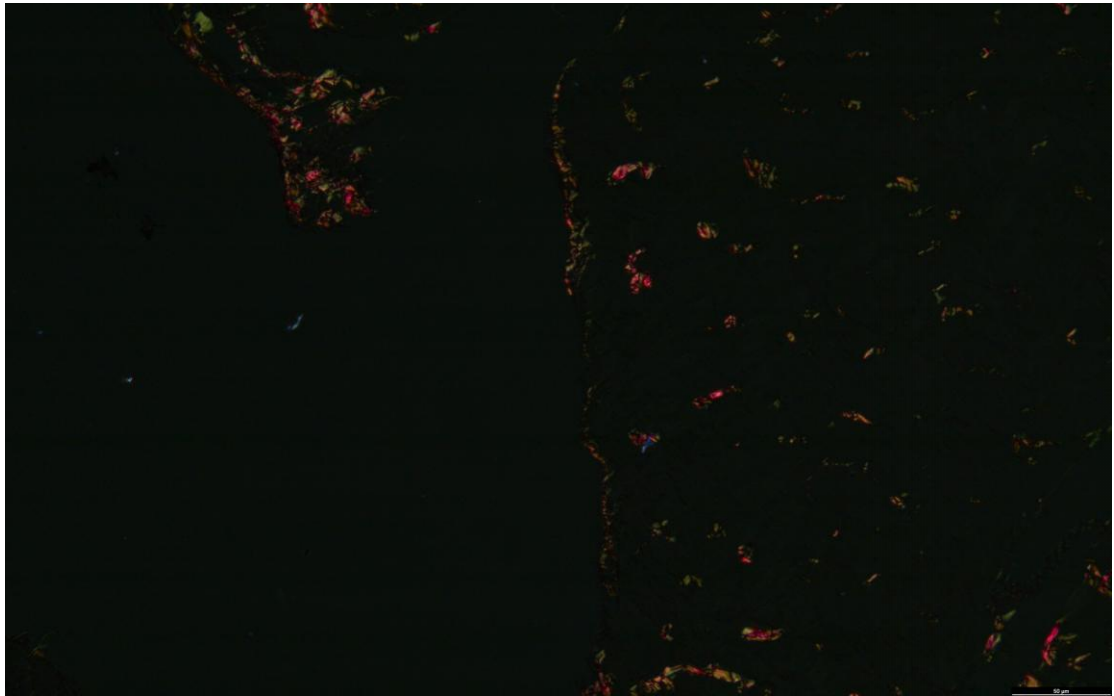

Model

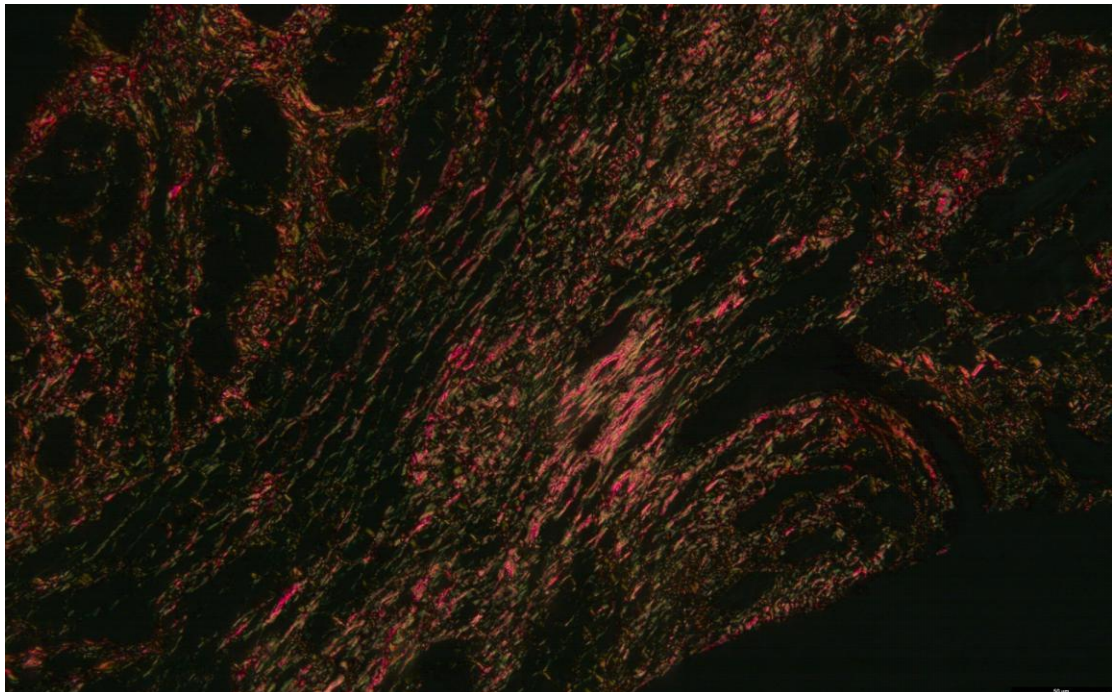

QL-L

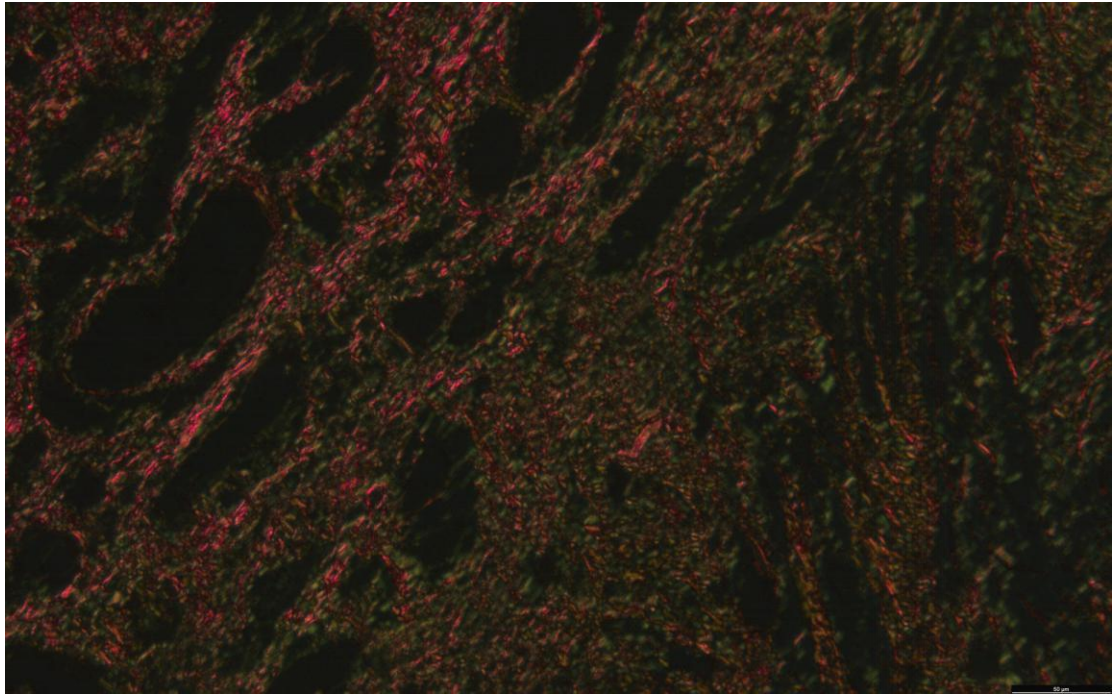

QL-H

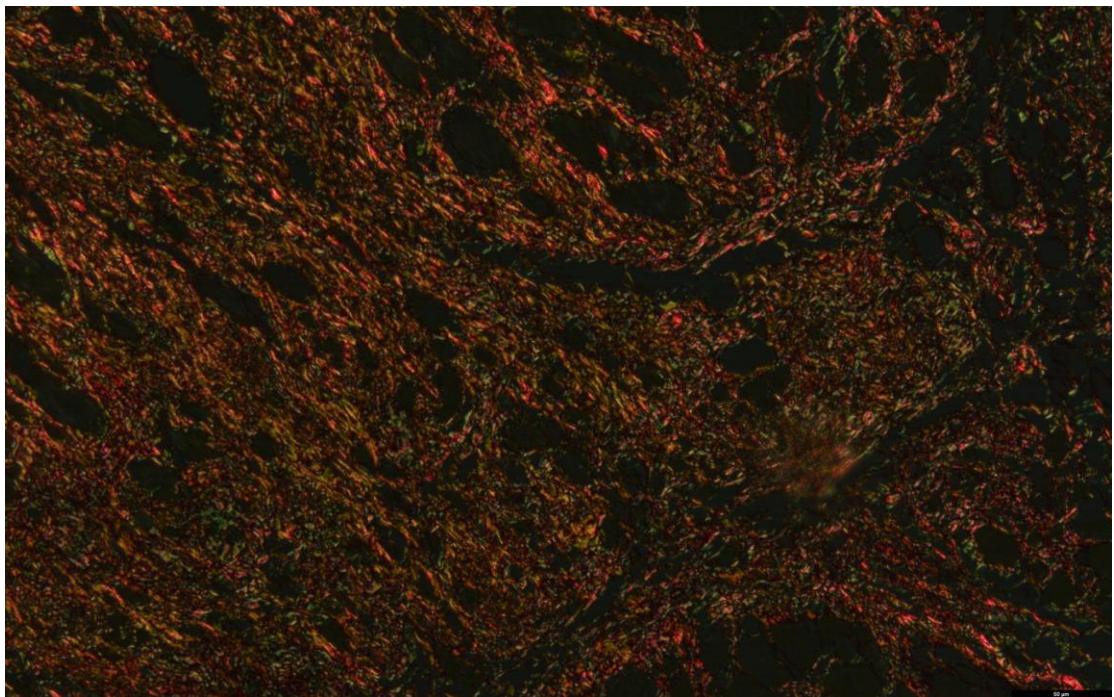

Empagliflozin

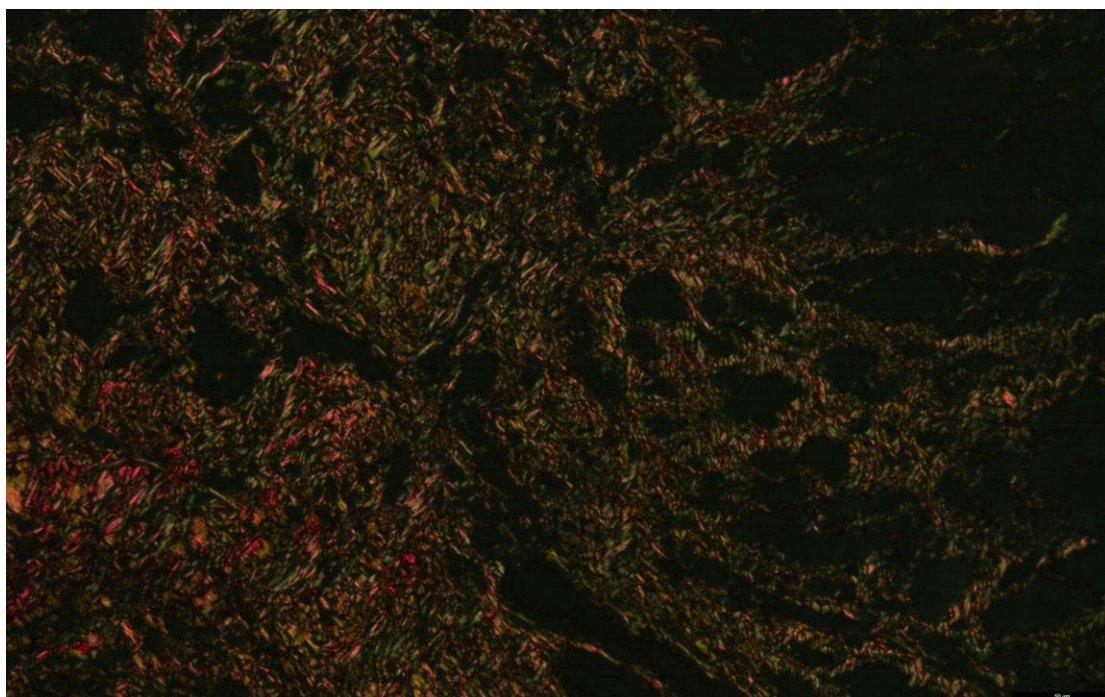

Supplement: S7 Fig — (ZIP) [file pone.0310897.s007.zip › S7 Fig/PSR 20X.pdf]

## PSR 50X

Sham

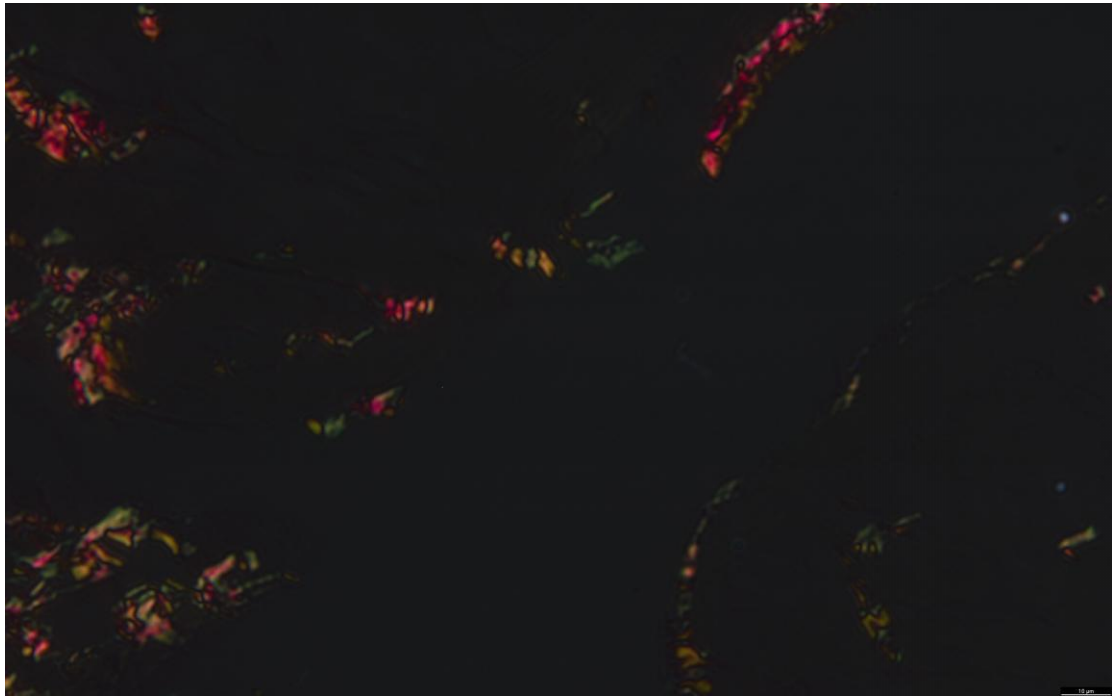

Model

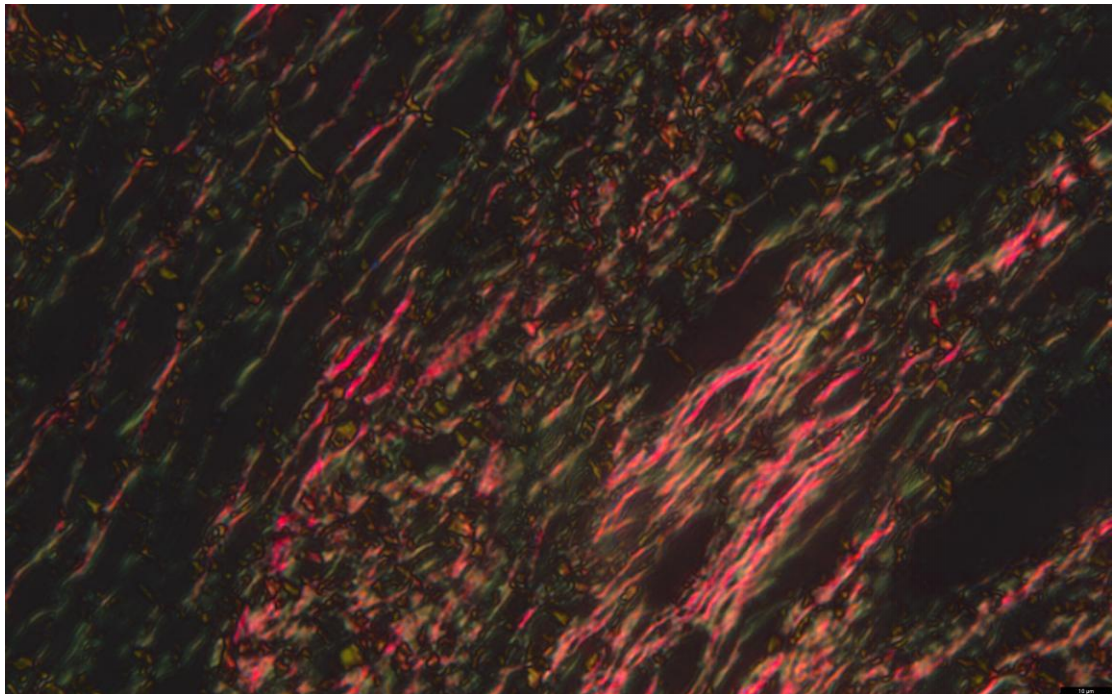

QL-L

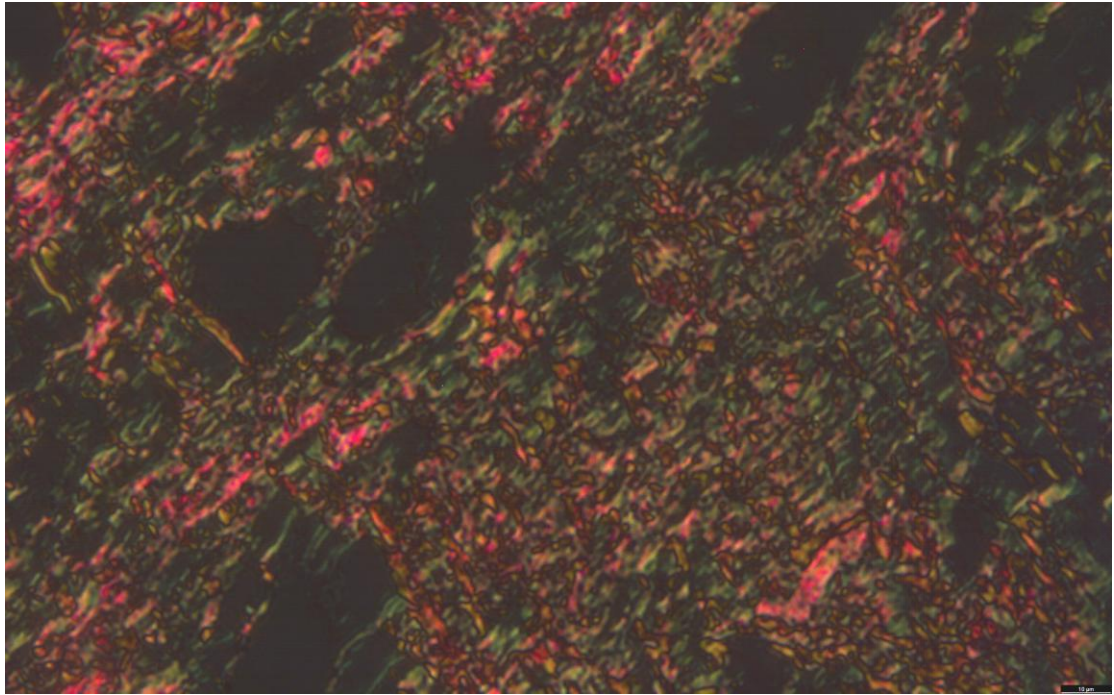

QL-H

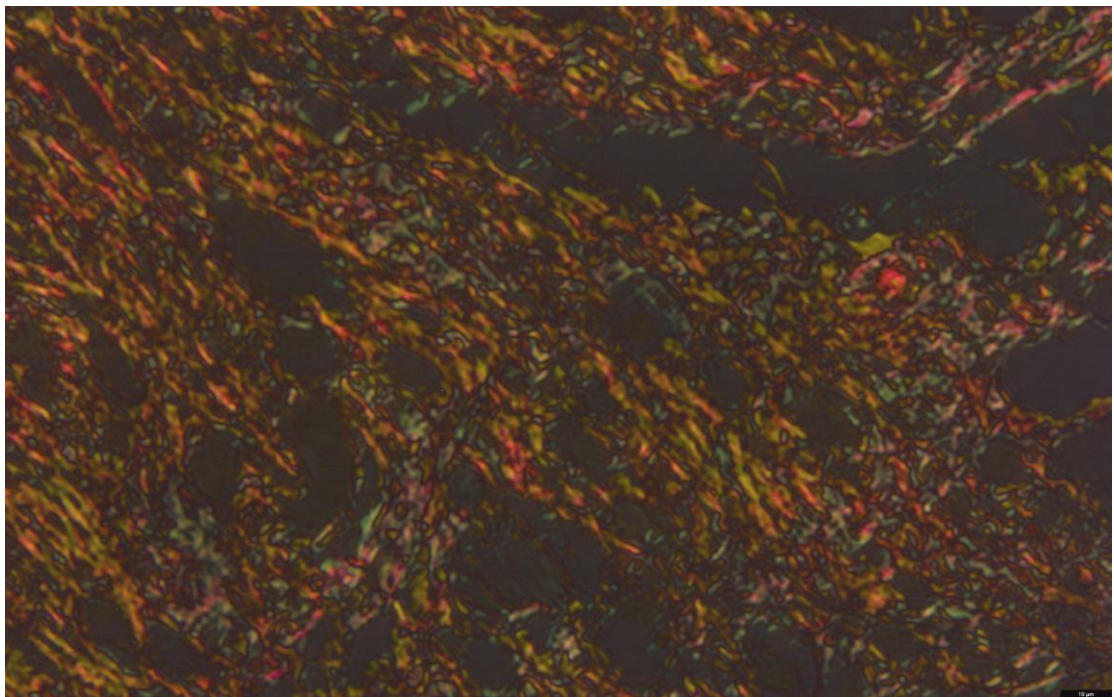

Empagliflozin

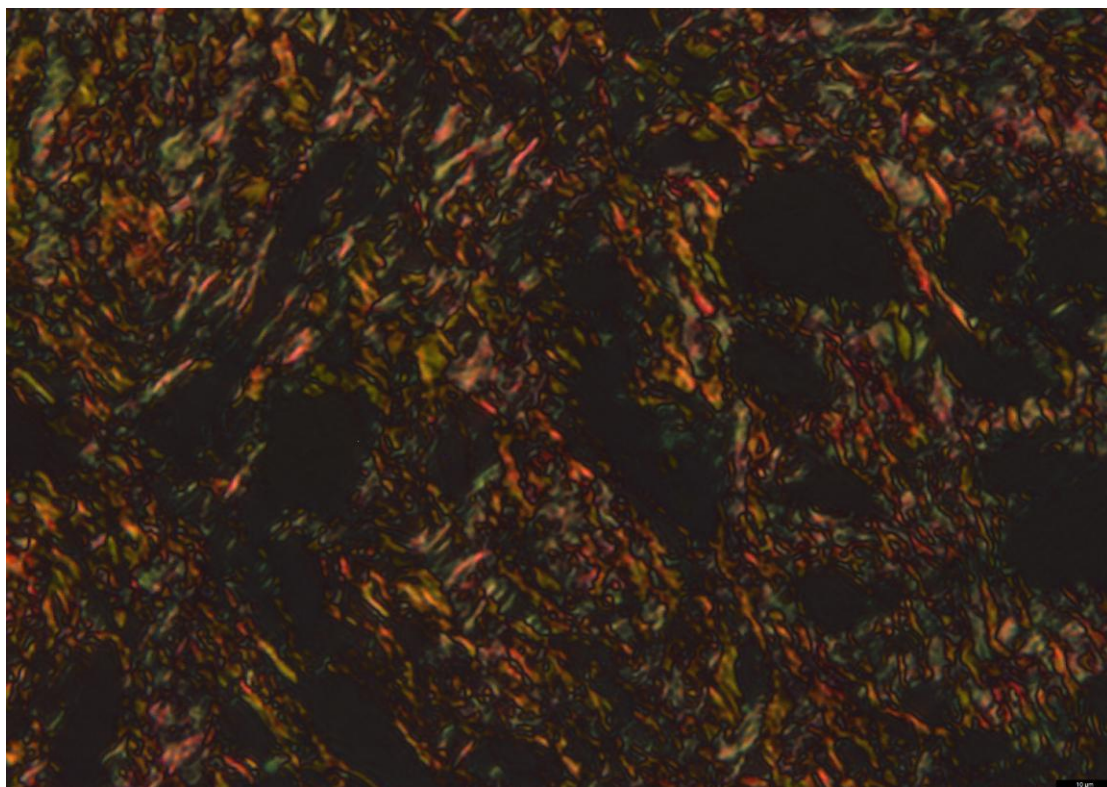

Supplement: S7 Fig — (ZIP) [file pone.0310897.s007.zip › S7 Fig/PSR 50X.pdf]

## PSR 5X

Sham

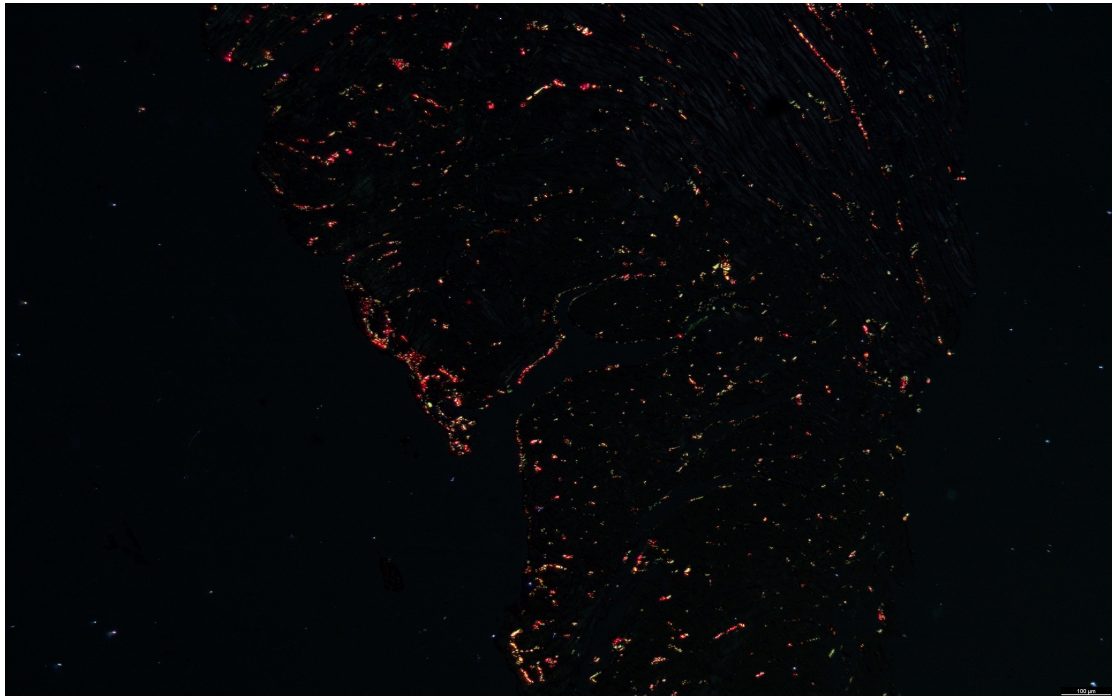

Model

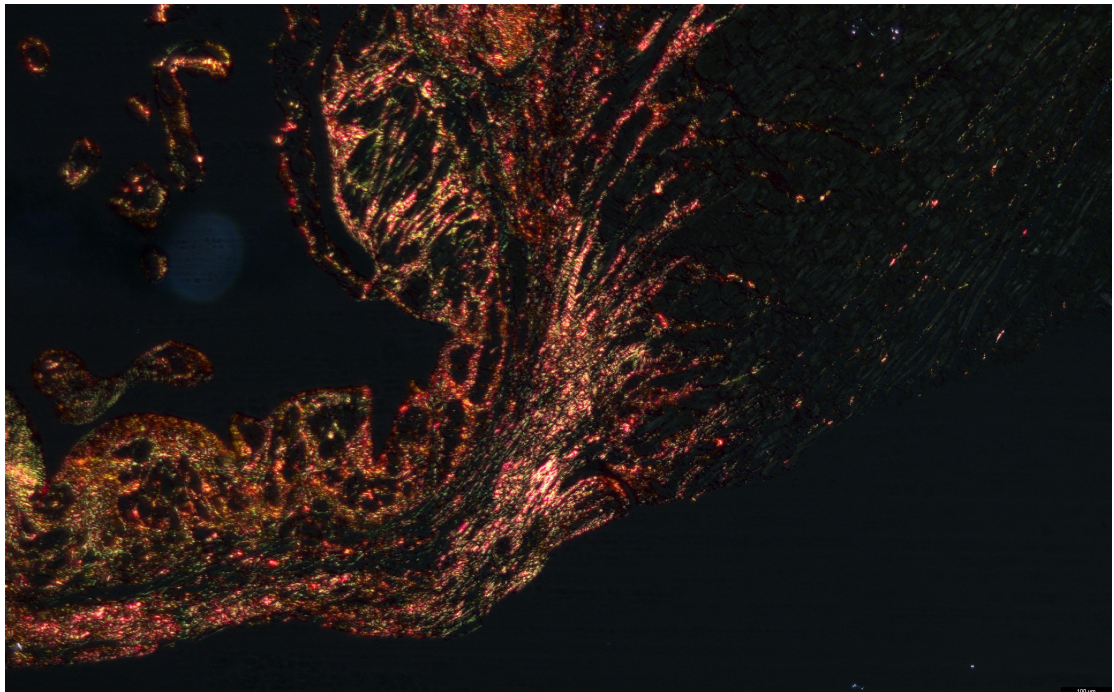

QL-L

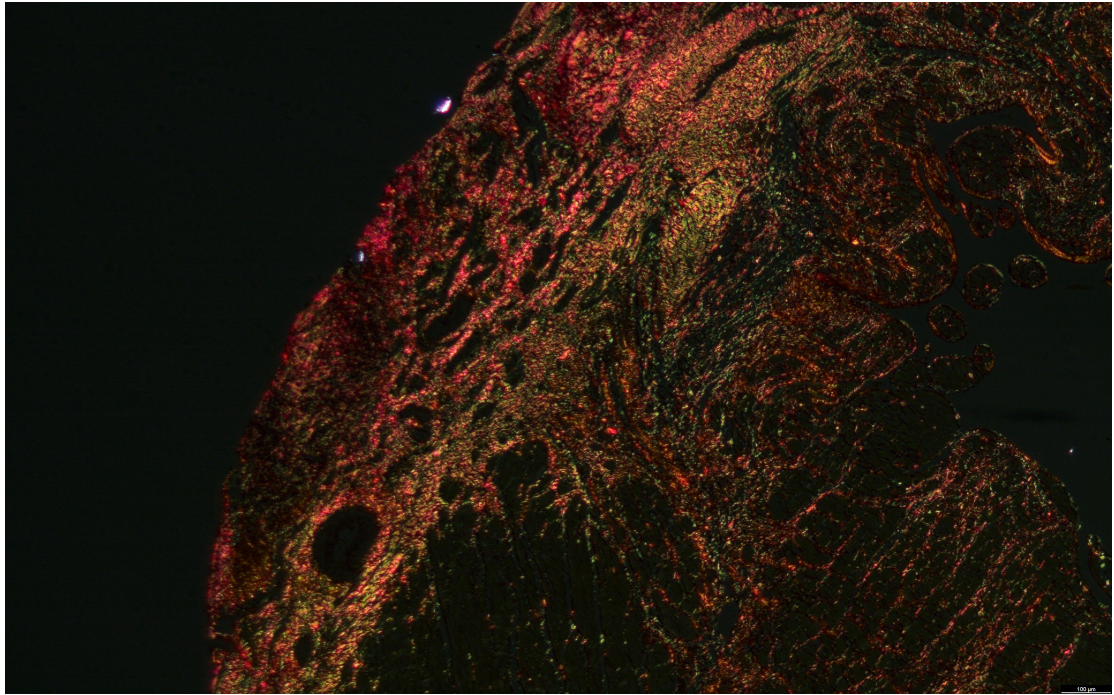

QL-H

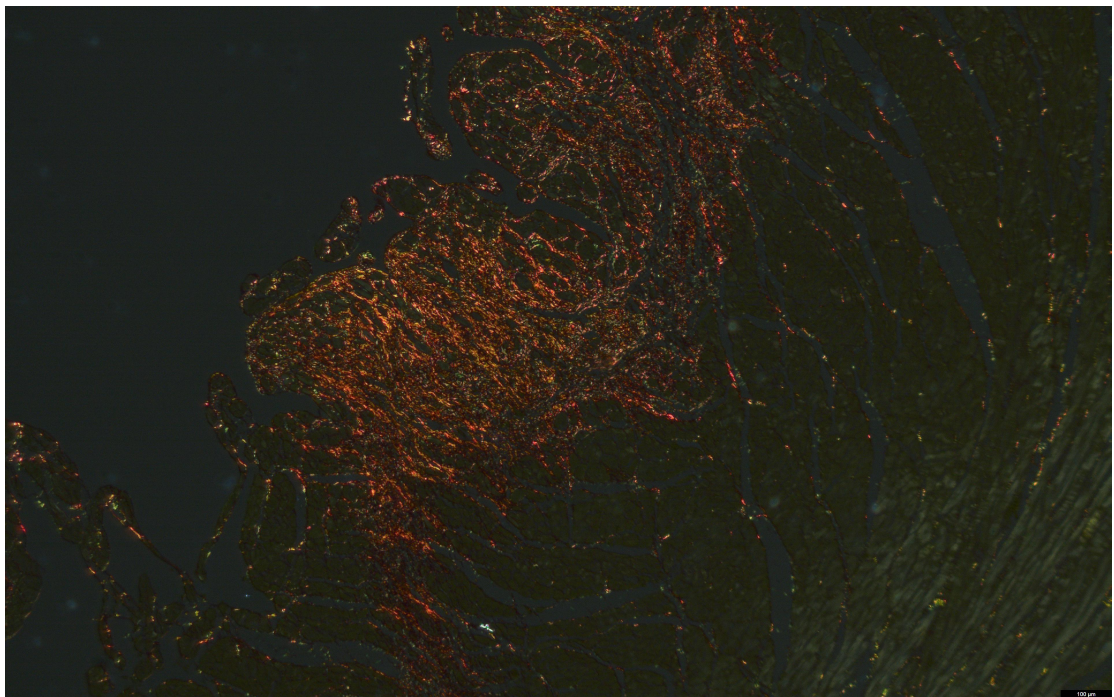

Empagliflozin

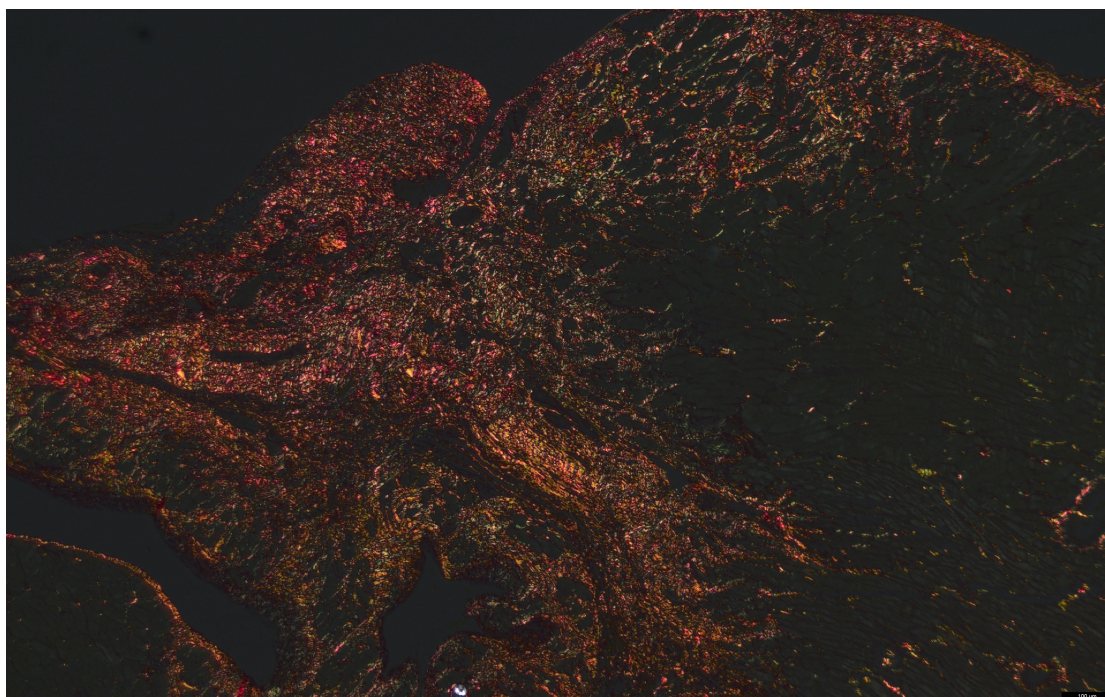

Supplement: S7 Fig — (ZIP) [file pone.0310897.s007.zip › S7 Fig/PSR 5X.pdf]

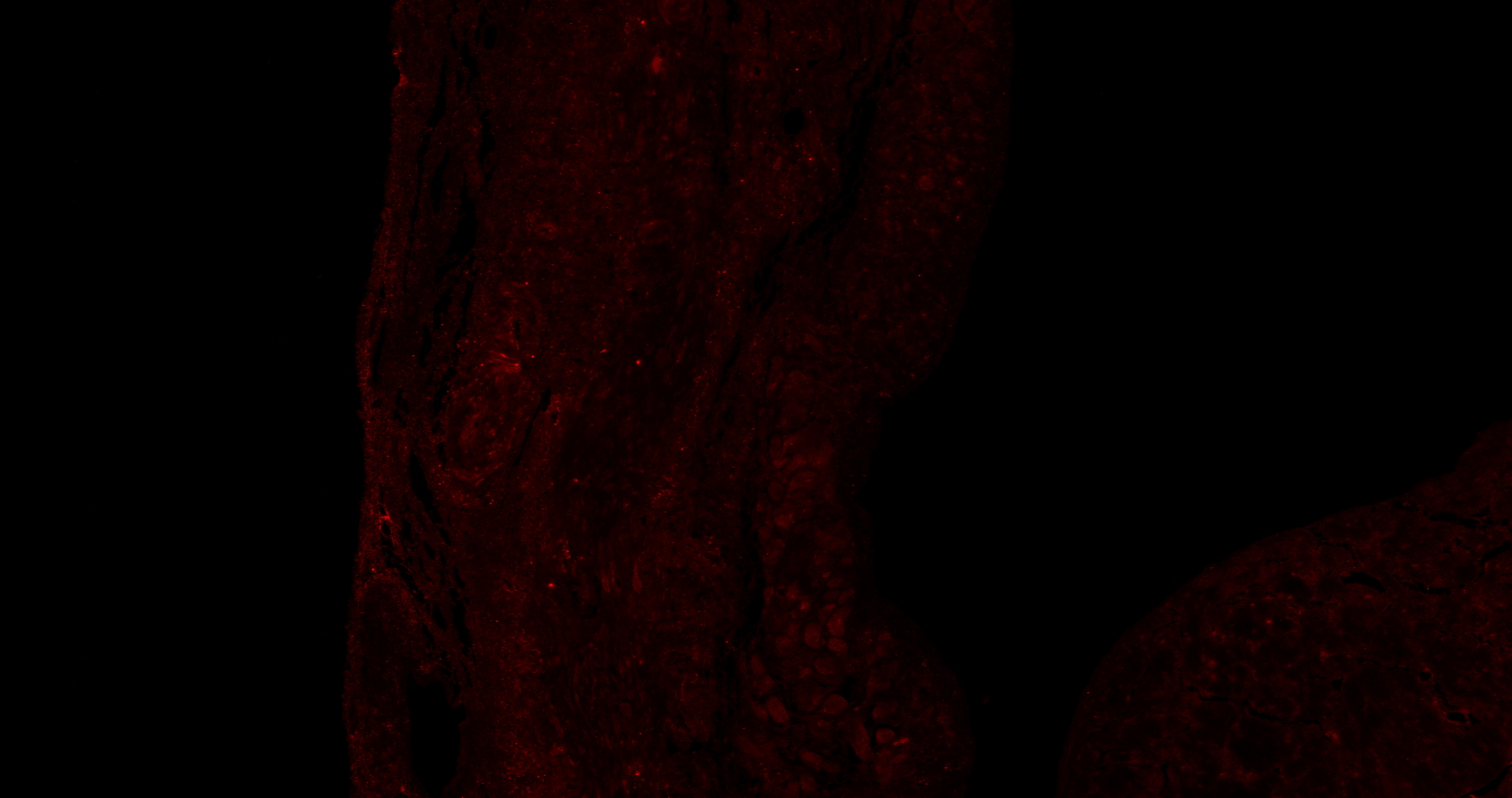

Supplement: S8 Fig — (ZIP) [file pone.0310897.s008.zip › S8 Fig/Fig5A1 MMP9/Empagliflozin.tif]

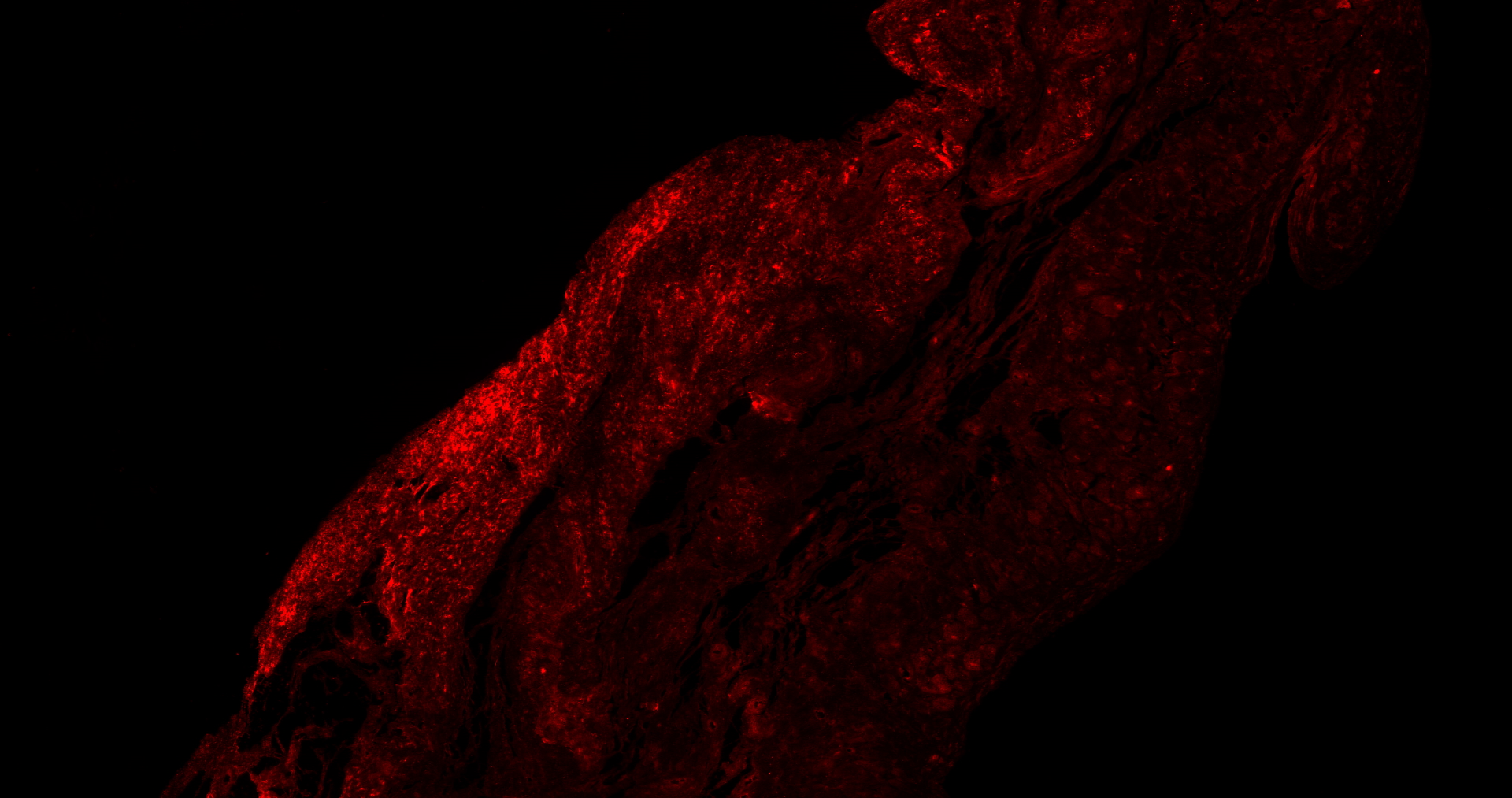

Supplement: S8 Fig — (ZIP) [file pone.0310897.s008.zip › S8 Fig/Fig5A1 MMP9/Model.tif]

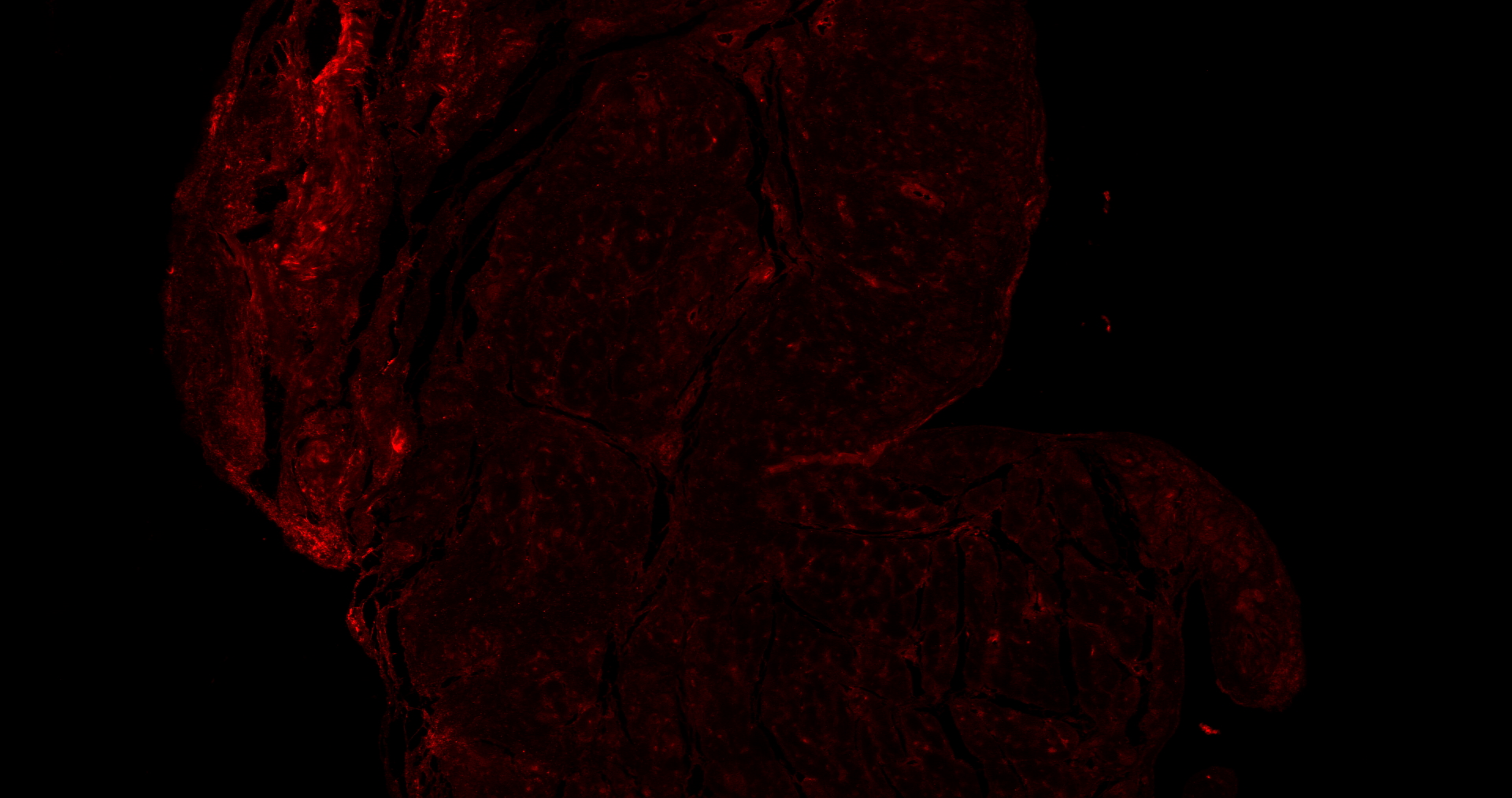

Supplement: S8 Fig — (ZIP) [file pone.0310897.s008.zip › S8 Fig/Fig5A1 MMP9/QL-H.tif]

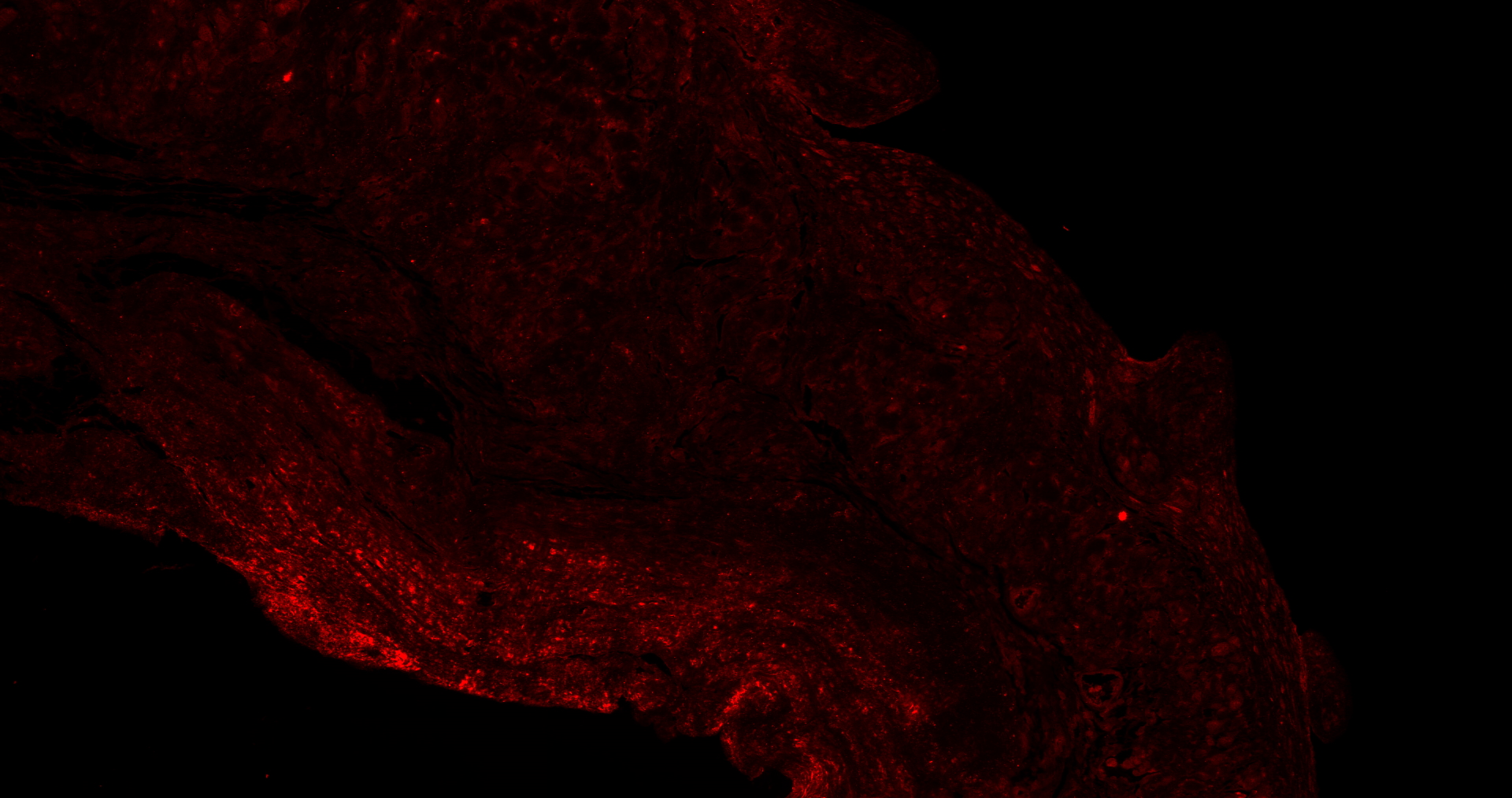

Supplement: S8 Fig — (ZIP) [file pone.0310897.s008.zip › S8 Fig/Fig5A1 MMP9/QL-L.tif]

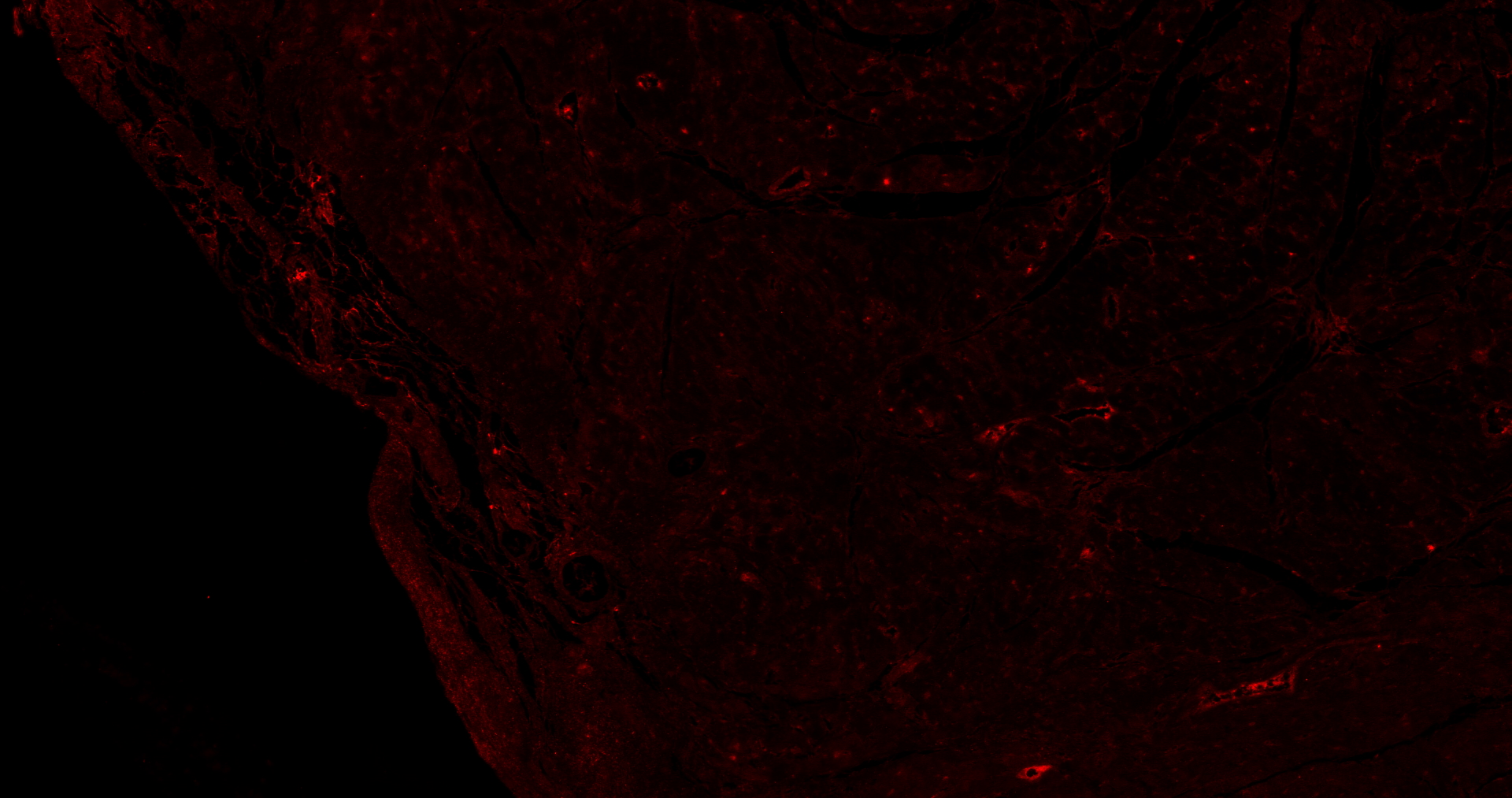

Supplement: S8 Fig — (ZIP) [file pone.0310897.s008.zip › S8 Fig/Fig5A1 MMP9/Sham.tif]

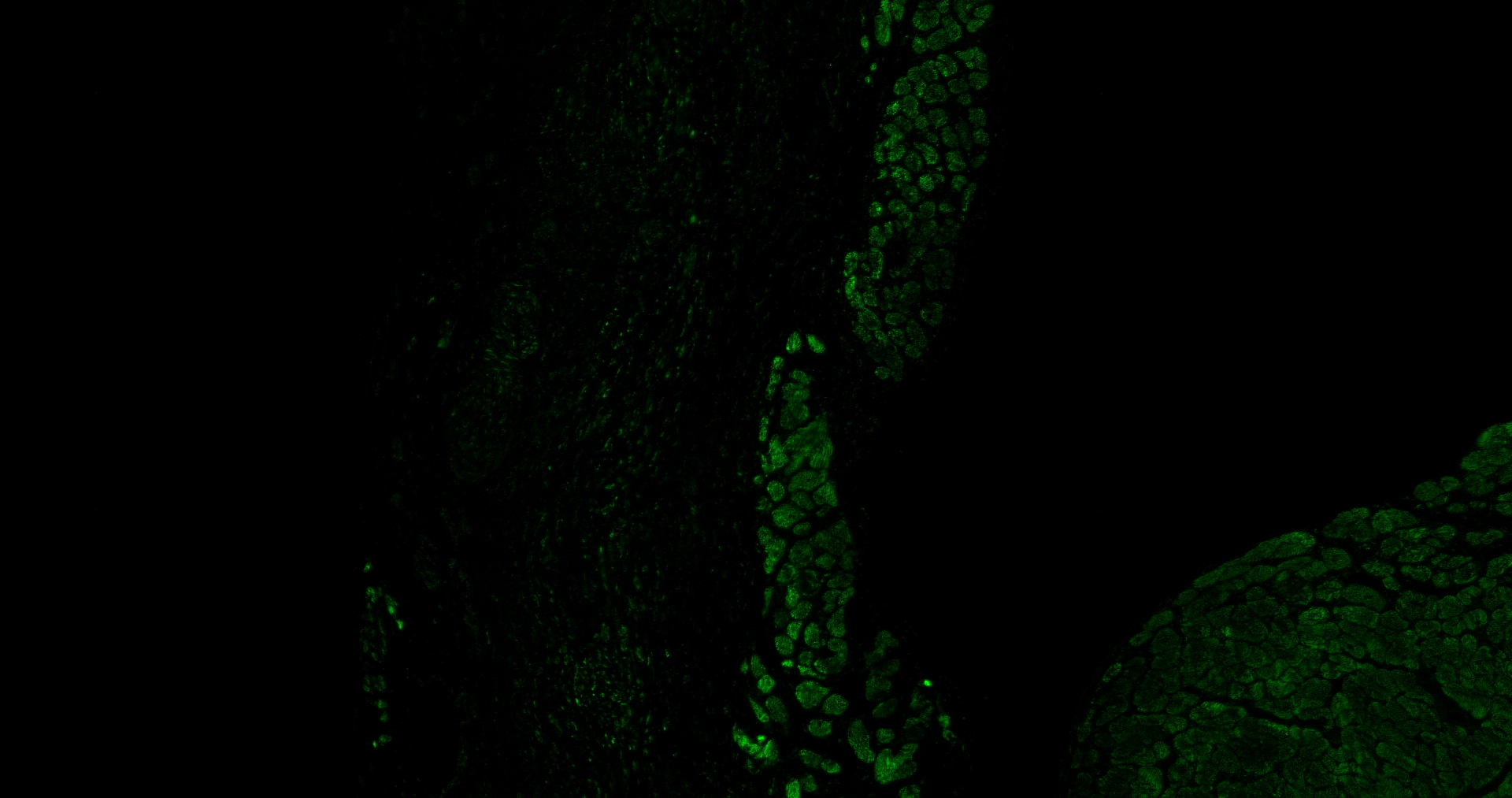

Supplement: S8 Fig — (ZIP) [file pone.0310897.s008.zip › S8 Fig/Fig5A2 TIMP-1/Empagliflozin.tif]

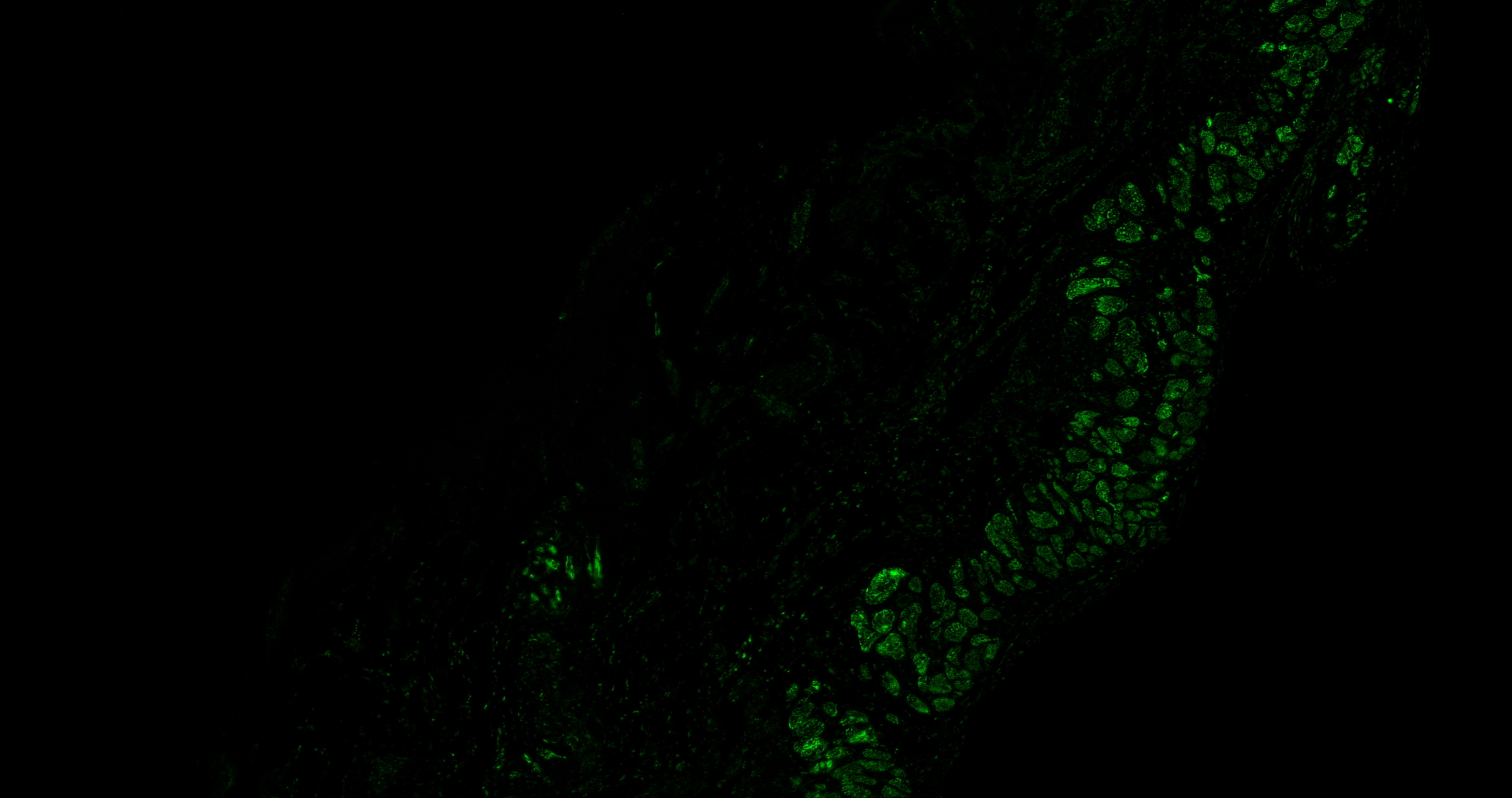

Supplement: S8 Fig — (ZIP) [file pone.0310897.s008.zip › S8 Fig/Fig5A2 TIMP-1/Model.tif]

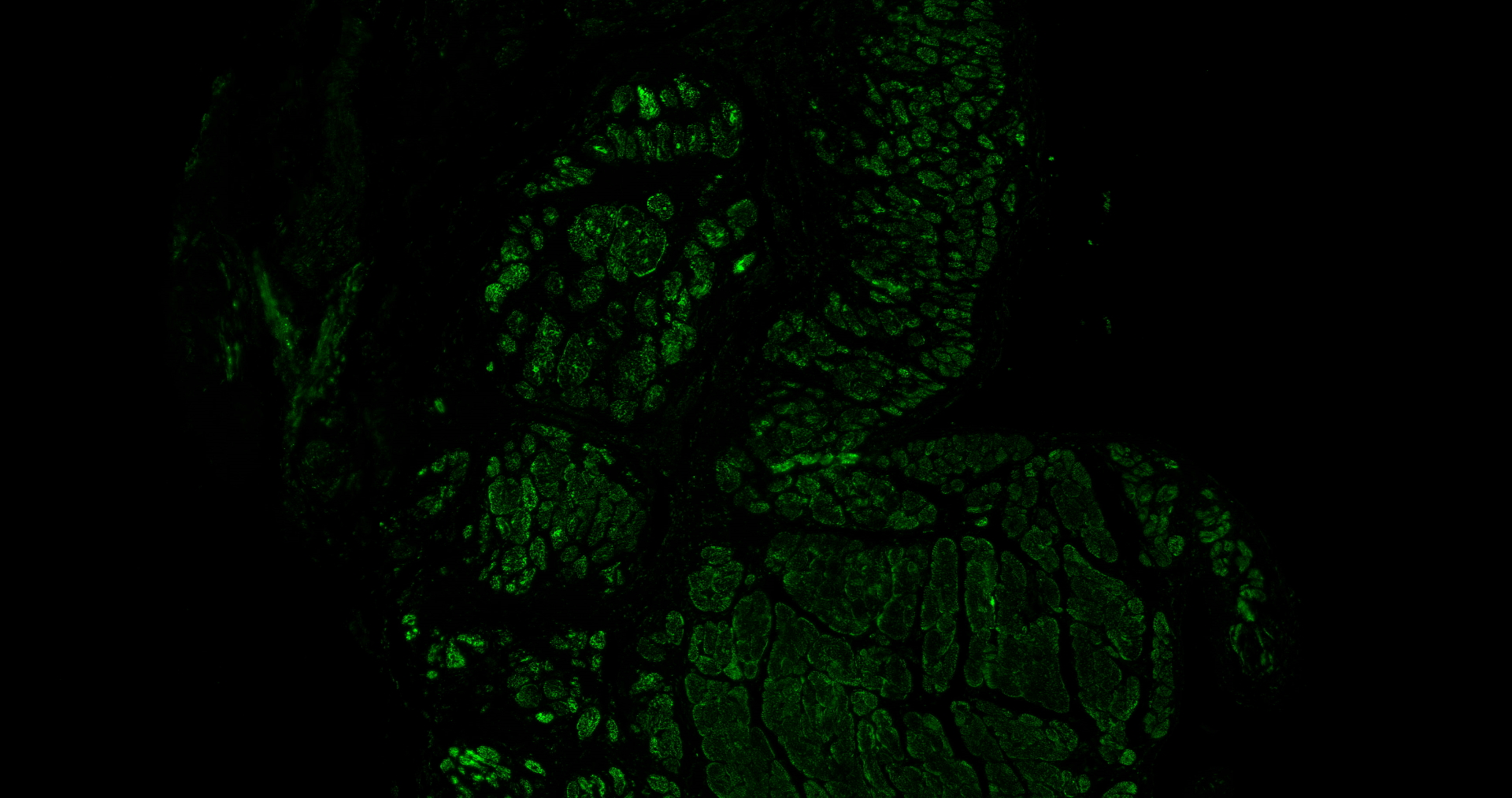

Supplement: S8 Fig — (ZIP) [file pone.0310897.s008.zip › S8 Fig/Fig5A2 TIMP-1/QL-H.tif]

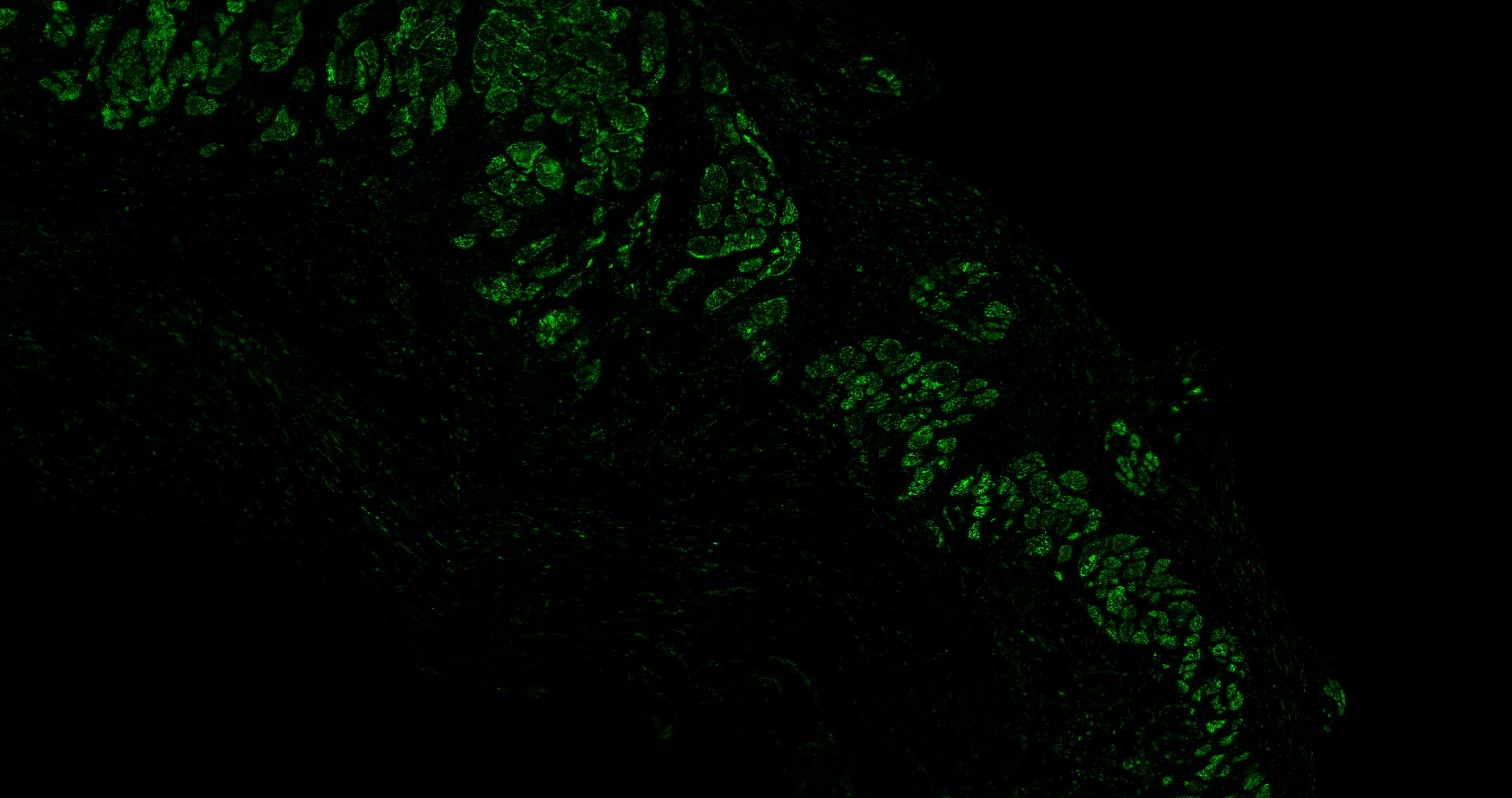

Supplement: S8 Fig — (ZIP) [file pone.0310897.s008.zip › S8 Fig/Fig5A2 TIMP-1/QL-L.tif]

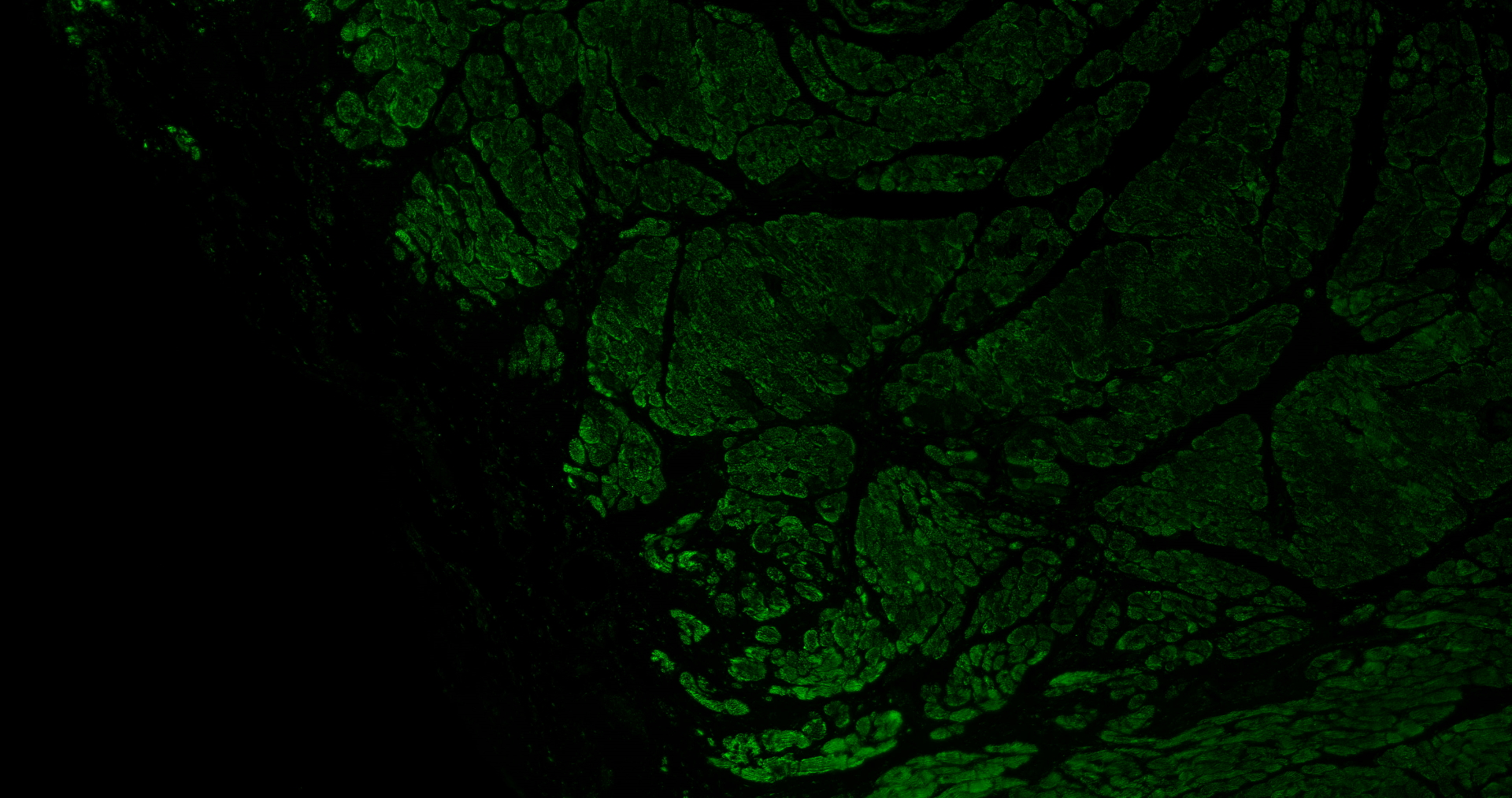

Supplement: S8 Fig — (ZIP) [file pone.0310897.s008.zip › S8 Fig/Fig5A2 TIMP-1/Sham.tif]

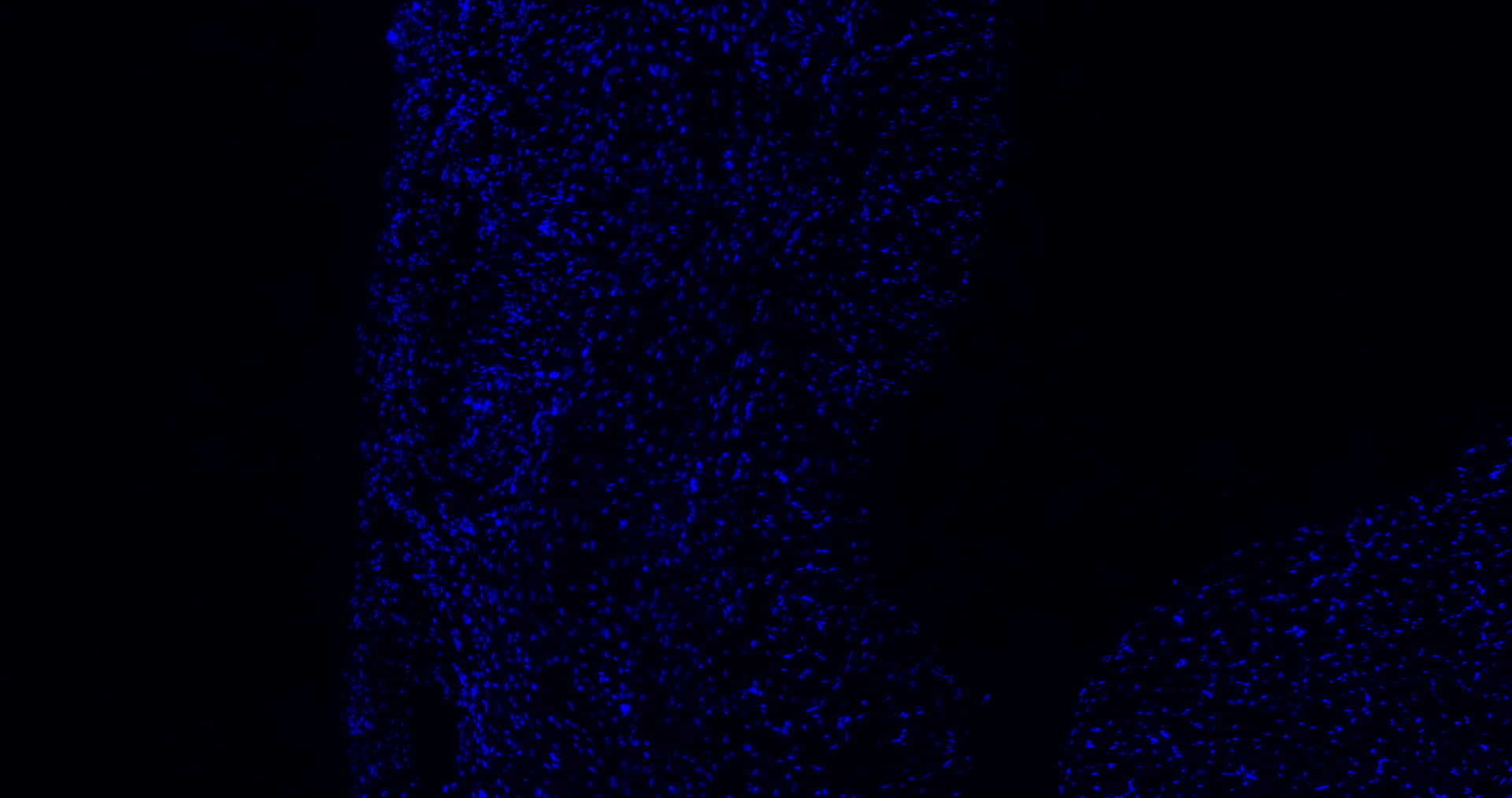

Supplement: S8 Fig — (ZIP) [file pone.0310897.s008.zip › S8 Fig/Fig5A3 DAPI/Empagliflozin.tif]

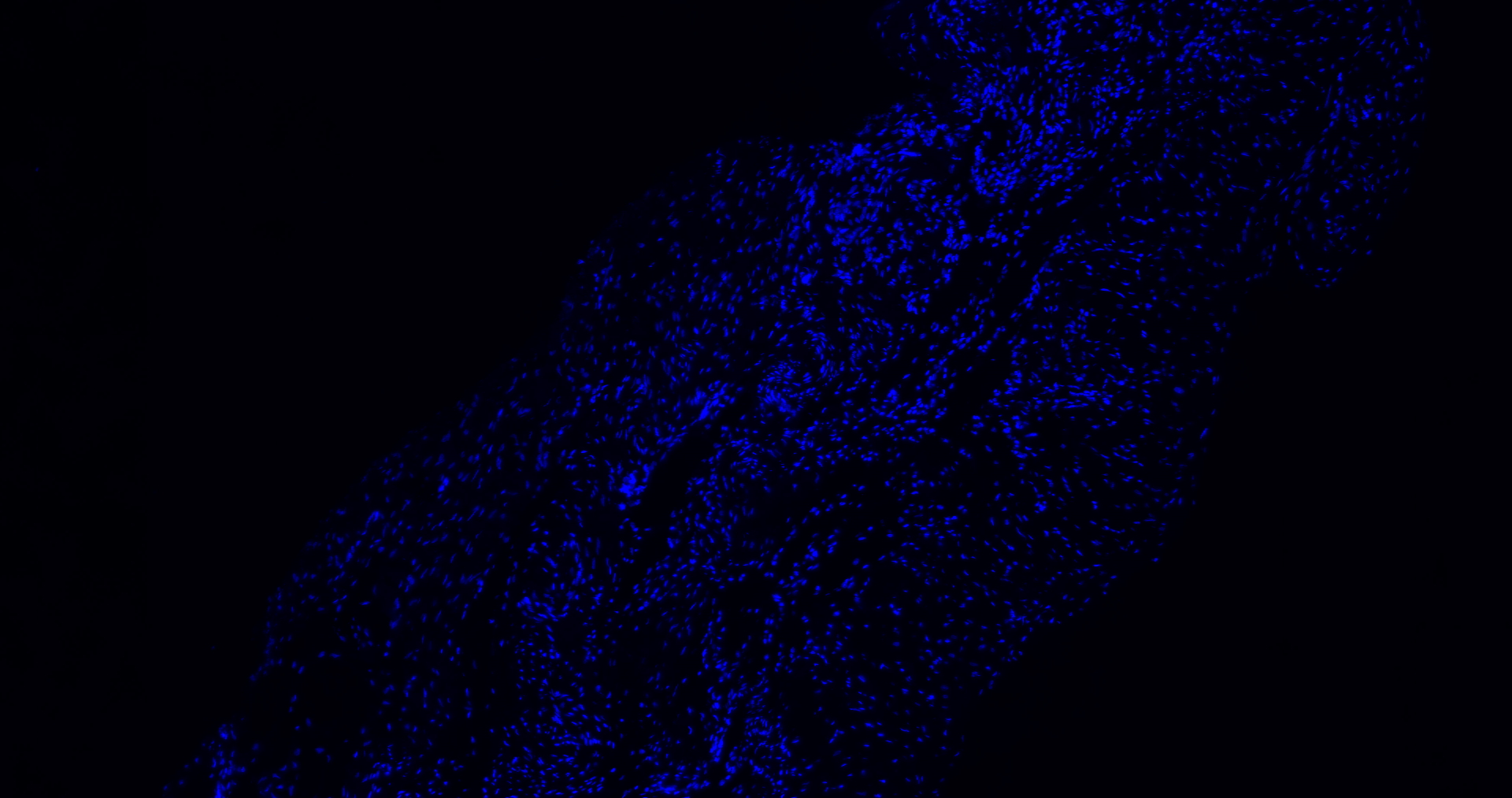

Supplement: S8 Fig — (ZIP) [file pone.0310897.s008.zip › S8 Fig/Fig5A3 DAPI/Model.tif]

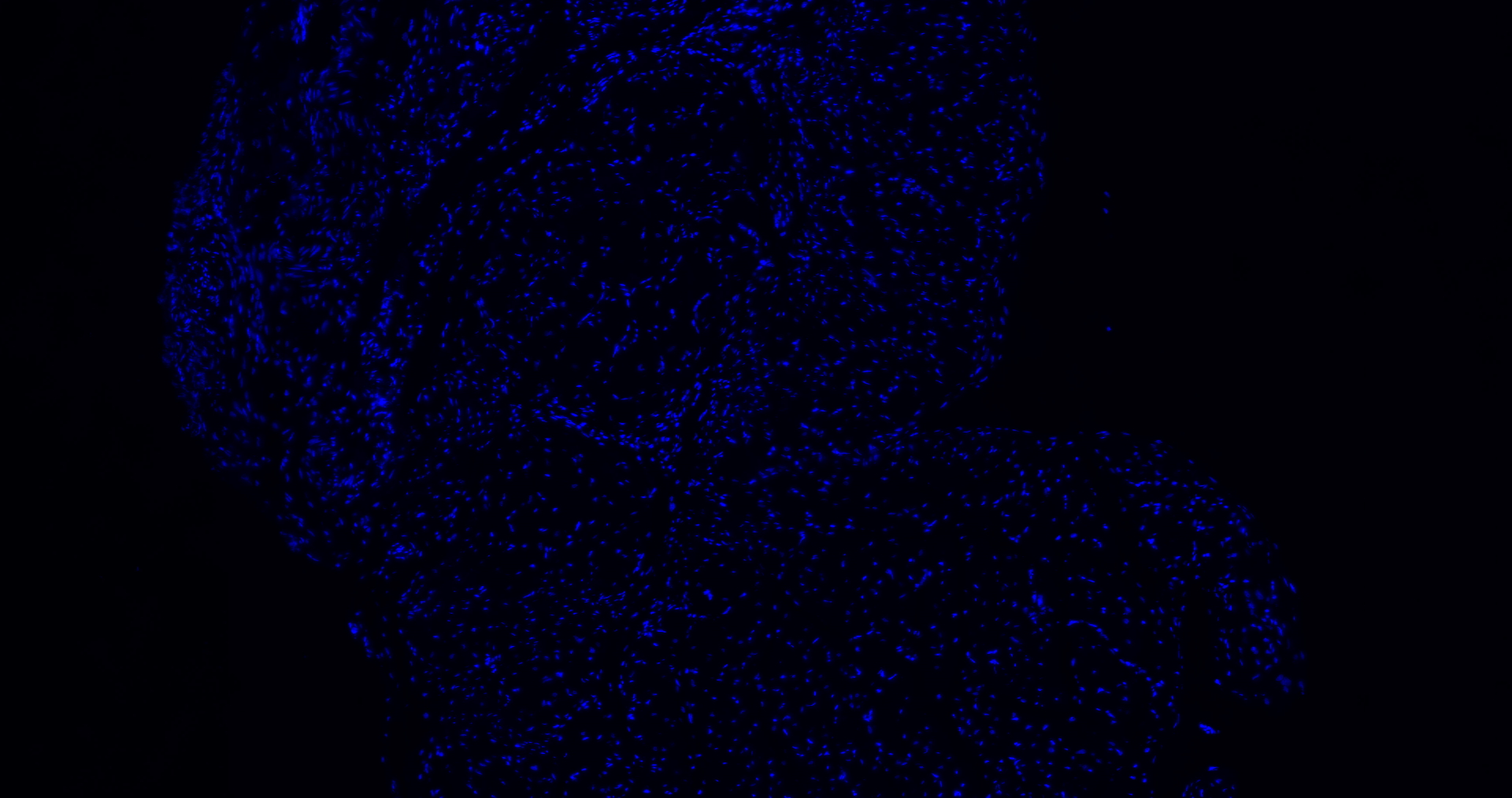

Supplement: S8 Fig — (ZIP) [file pone.0310897.s008.zip › S8 Fig/Fig5A3 DAPI/QL-H.tif]

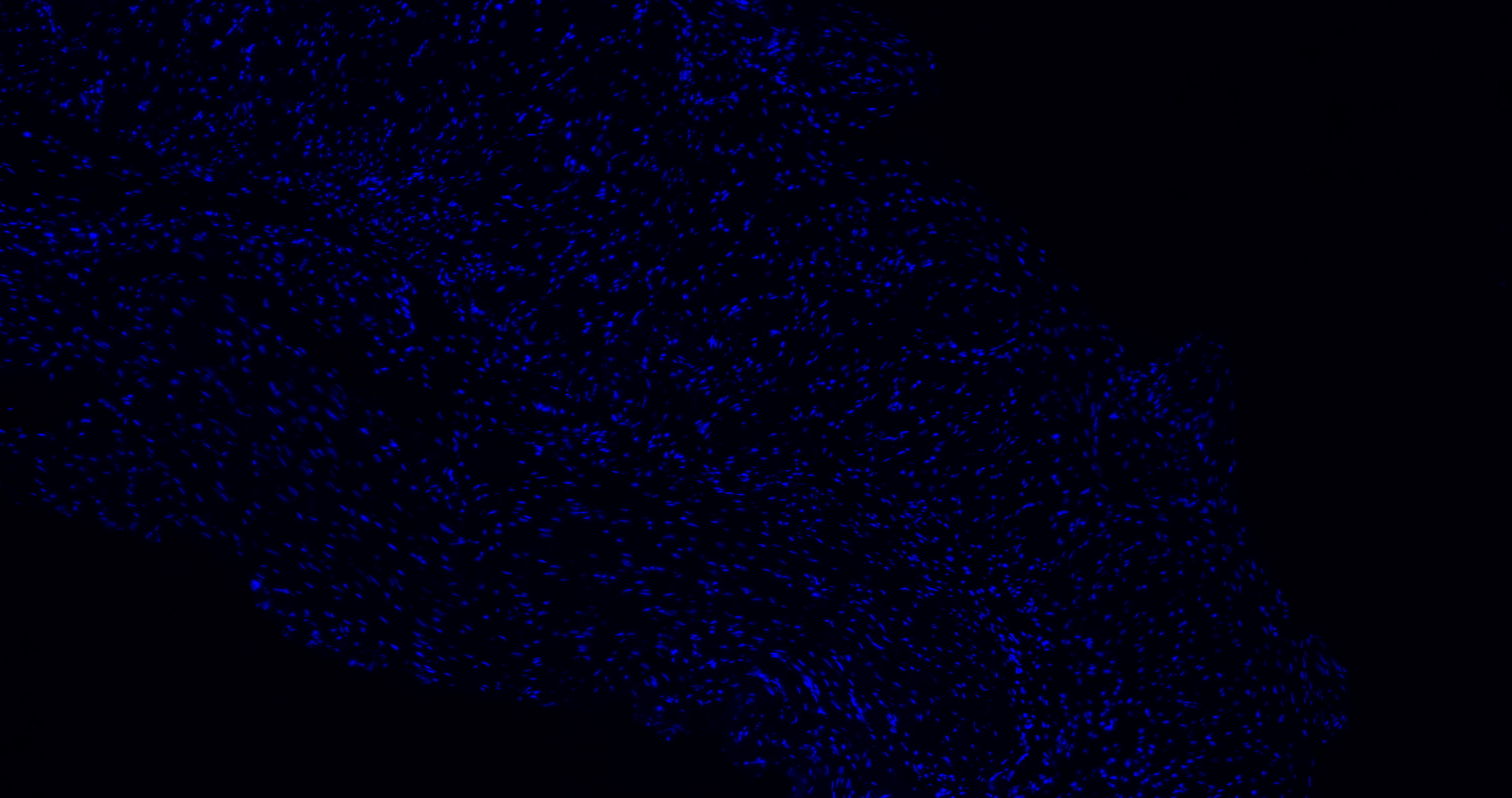

Supplement: S8 Fig — (ZIP) [file pone.0310897.s008.zip › S8 Fig/Fig5A3 DAPI/QL-L.tif]

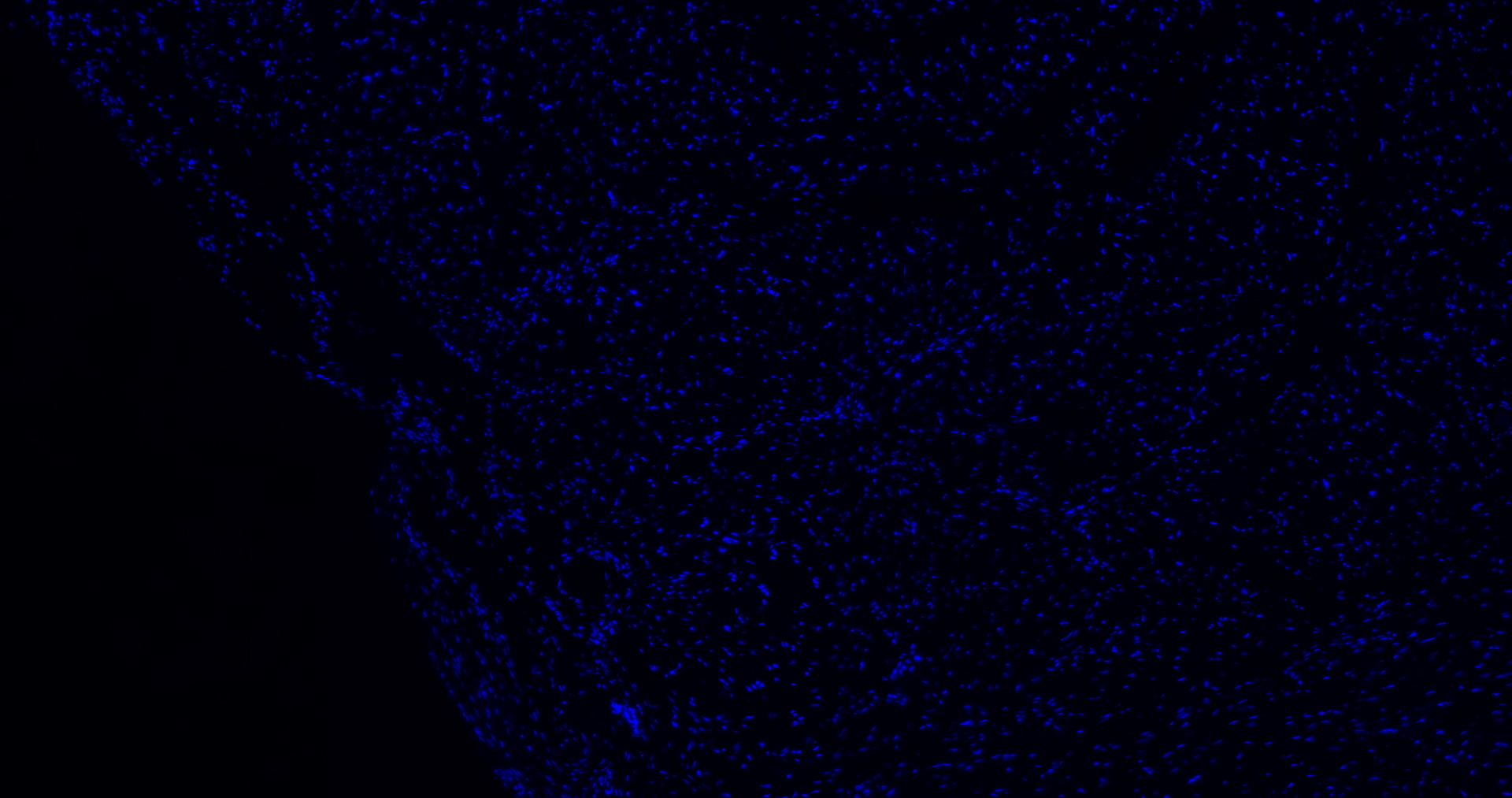

Supplement: S8 Fig — (ZIP) [file pone.0310897.s008.zip › S8 Fig/Fig5A3 DAPI/Sham.tif]

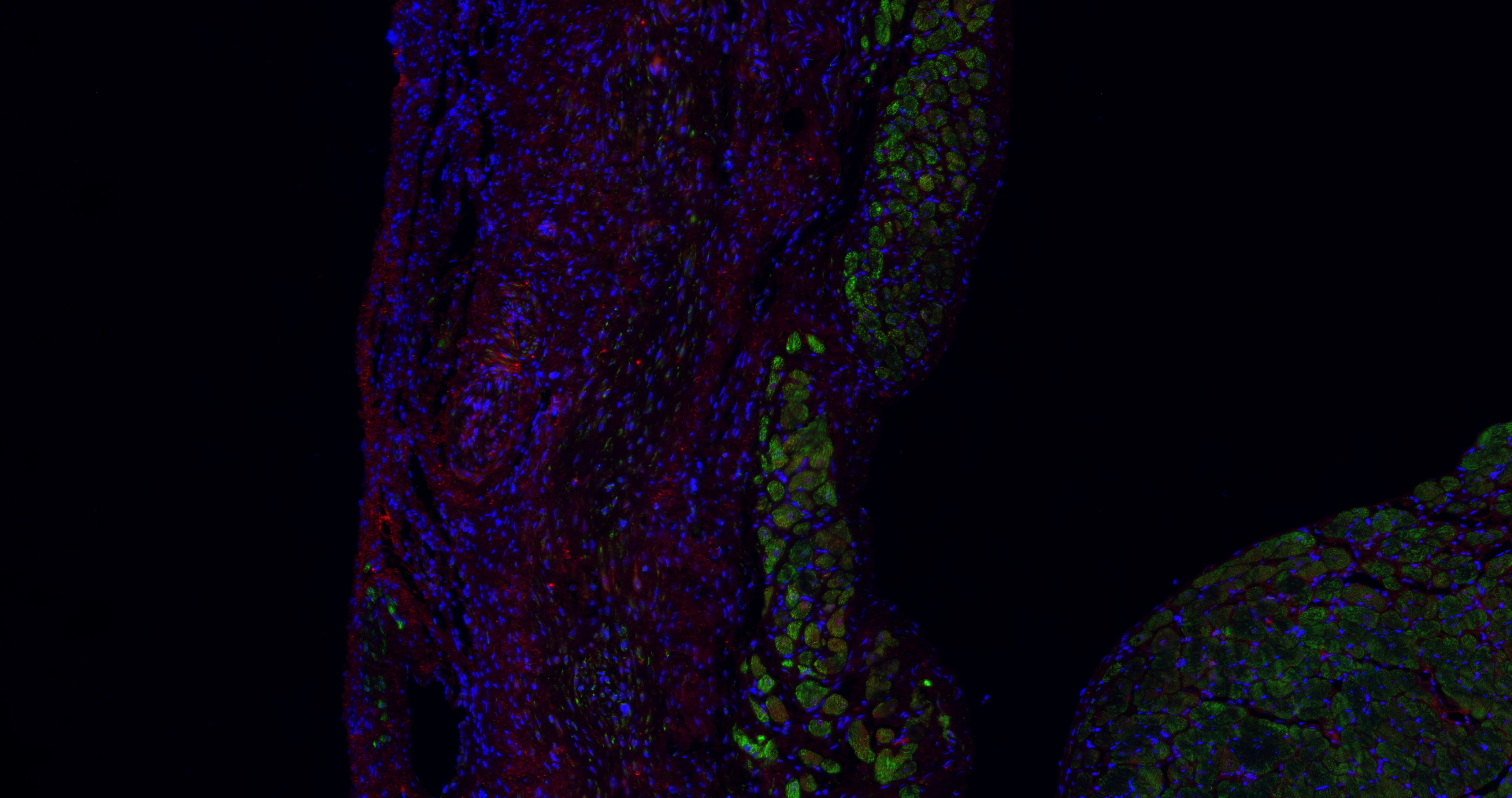

Supplement: S8 Fig — (ZIP) [file pone.0310897.s008.zip › S8 Fig/Fig5A4 Merge/Empagliflozin.tif]

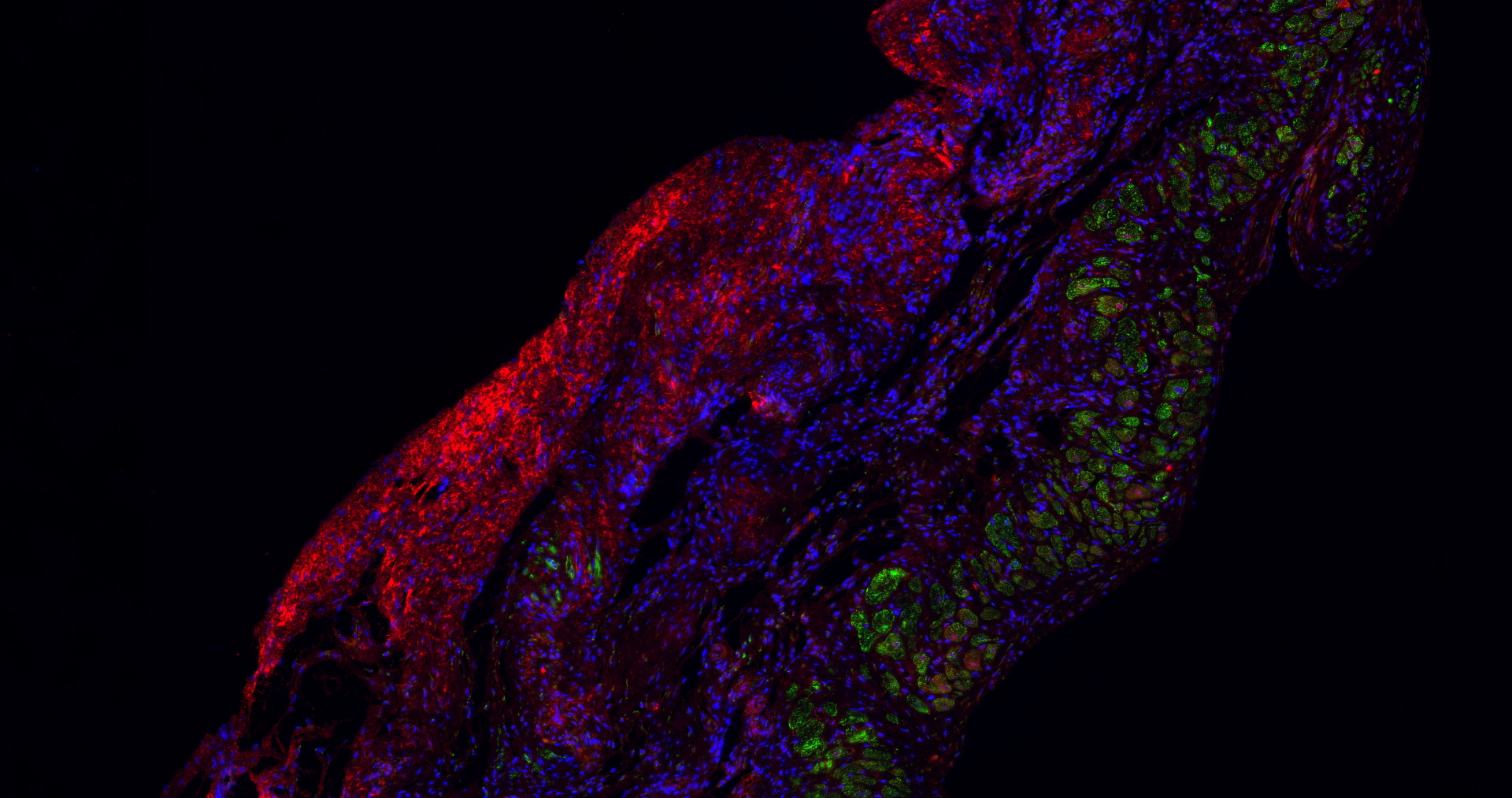

Supplement: S8 Fig — (ZIP) [file pone.0310897.s008.zip › S8 Fig/Fig5A4 Merge/Model.tif]

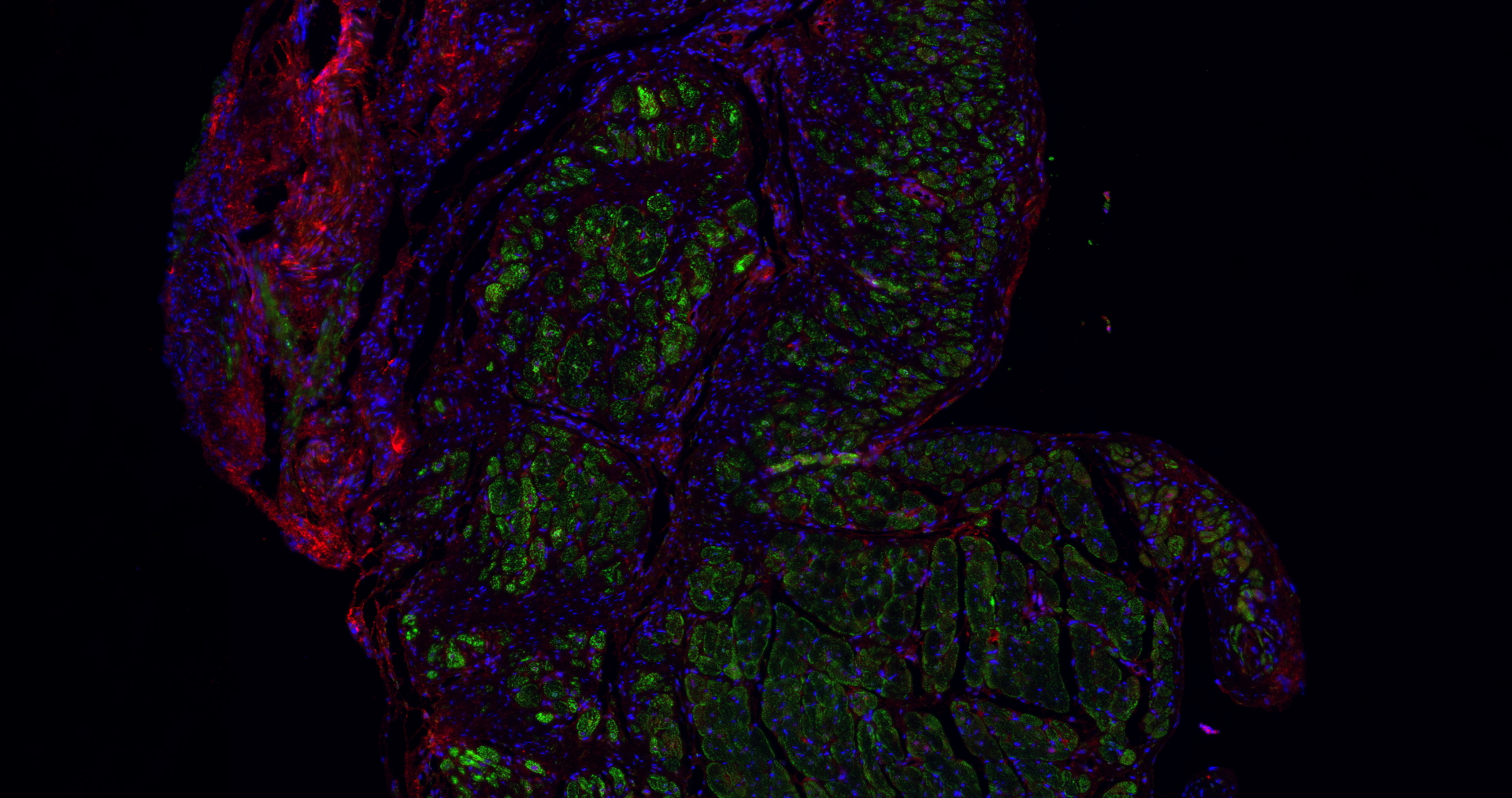

Supplement: S8 Fig — (ZIP) [file pone.0310897.s008.zip › S8 Fig/Fig5A4 Merge/QL-H.tif]

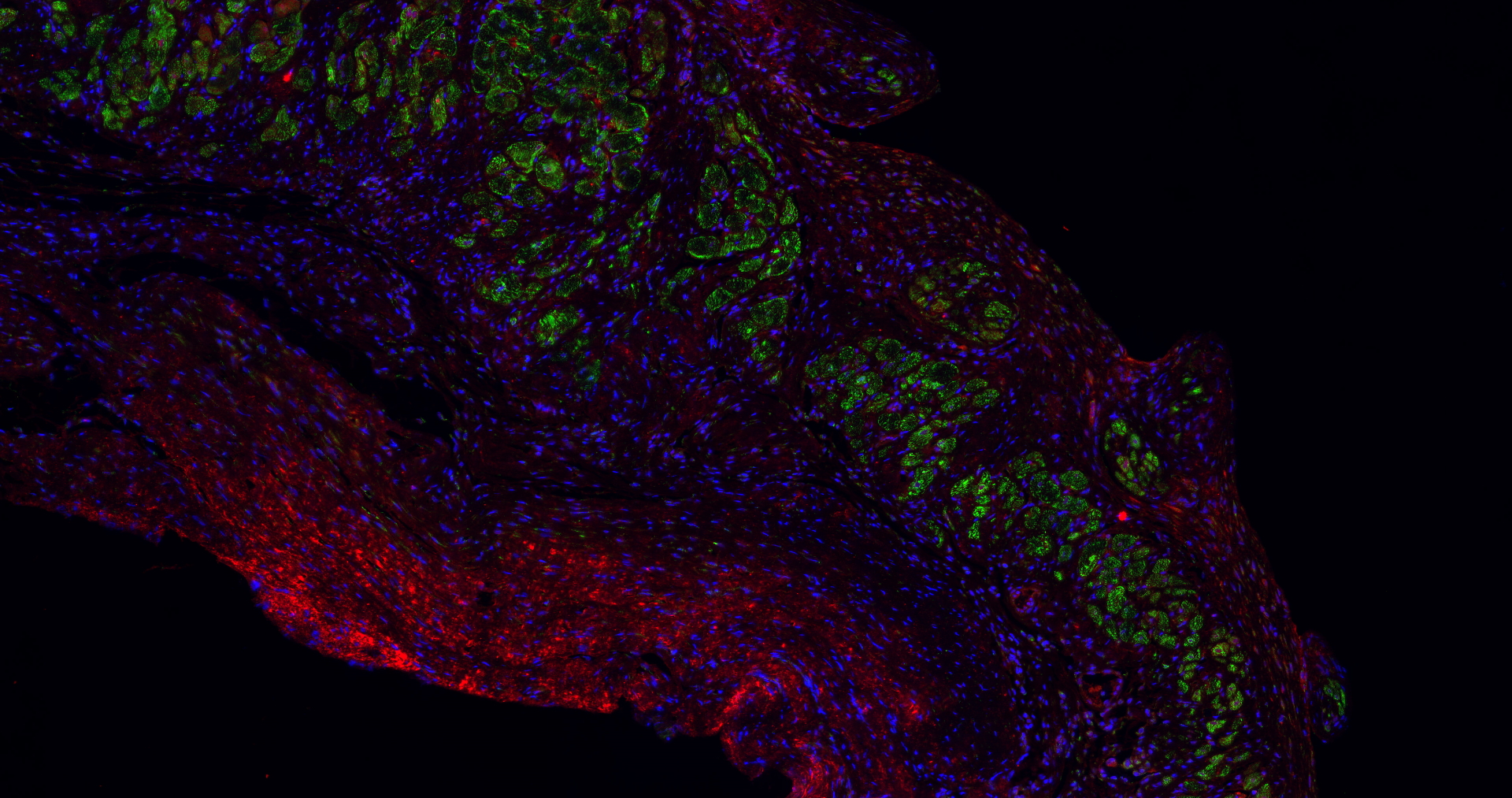

Supplement: S8 Fig — (ZIP) [file pone.0310897.s008.zip › S8 Fig/Fig5A4 Merge/QL-L.tif]

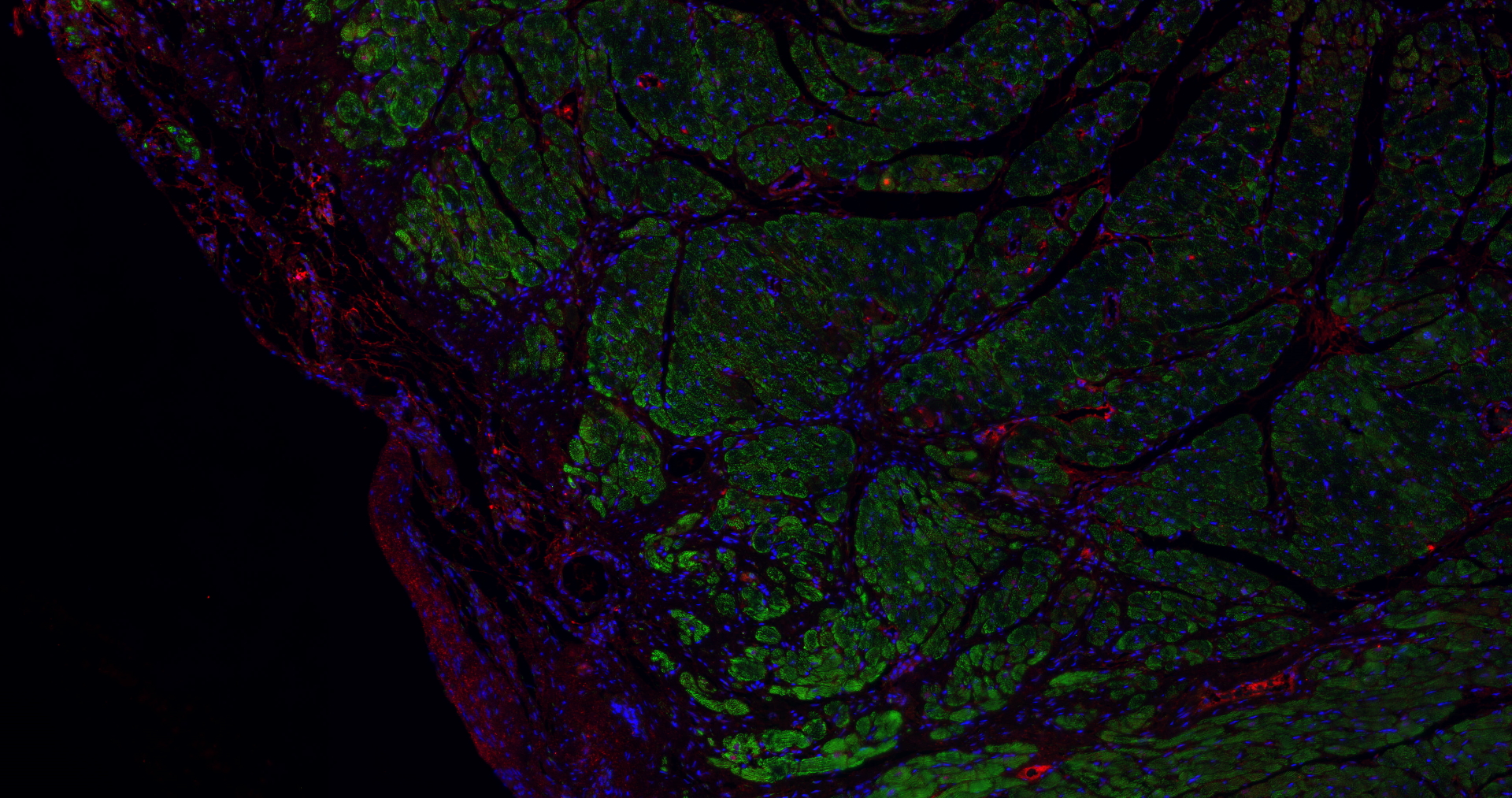

Supplement: S8 Fig — (ZIP) [file pone.0310897.s008.zip › S8 Fig/Fig5A4 Merge/Sham.tif]

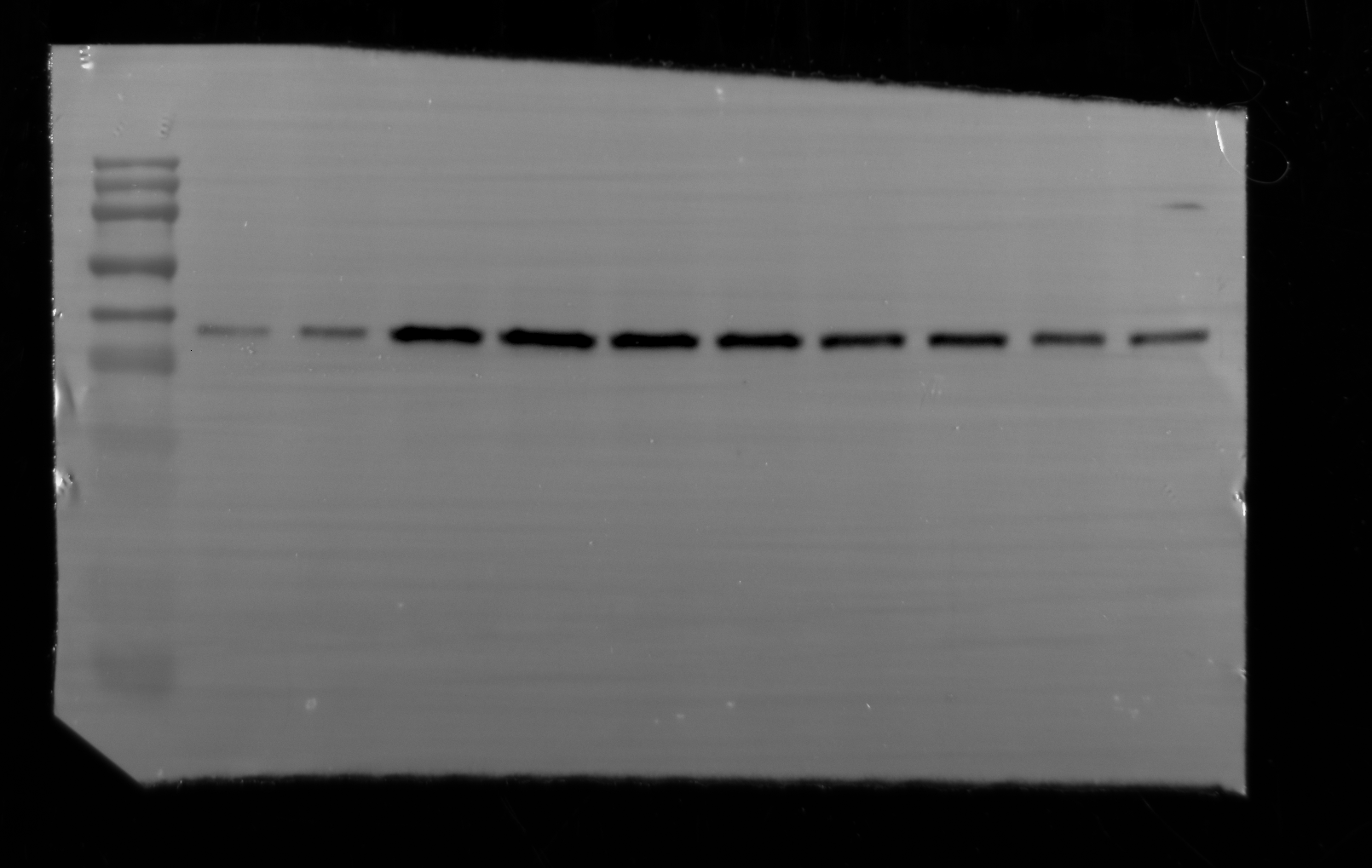

Supplement: S9 Fig — (A) Duplicate expression images for each target protein band. (B)Single expression image for each target protein band. (ZIP) [file pone.0310897.s009.zip › S9 Fig/A/AT1.tif]

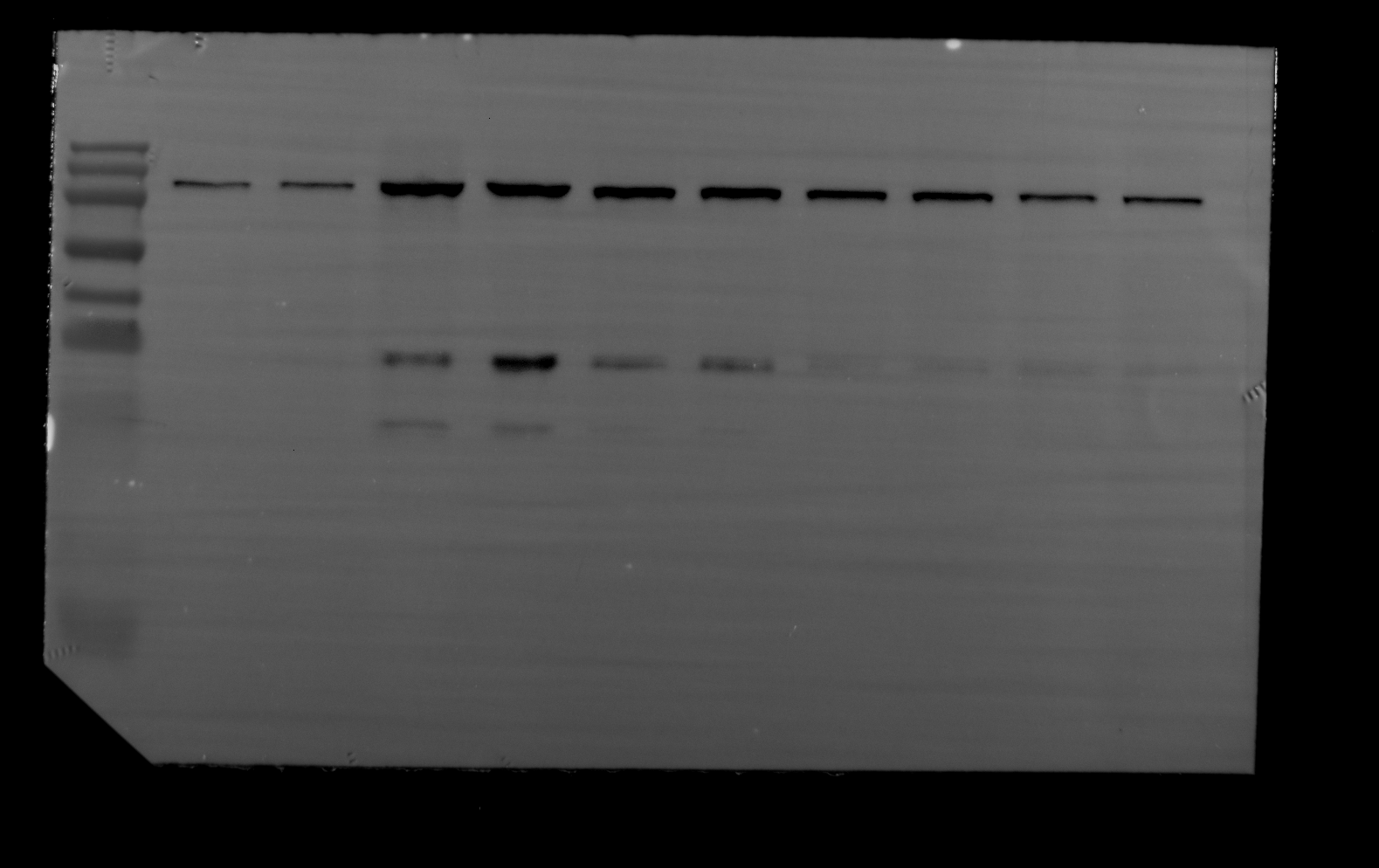

Supplement: S9 Fig — (A) Duplicate expression images for each target protein band. (B)Single expression image for each target protein band. (ZIP) [file pone.0310897.s009.zip › S9 Fig/A/Collagen I.tif]

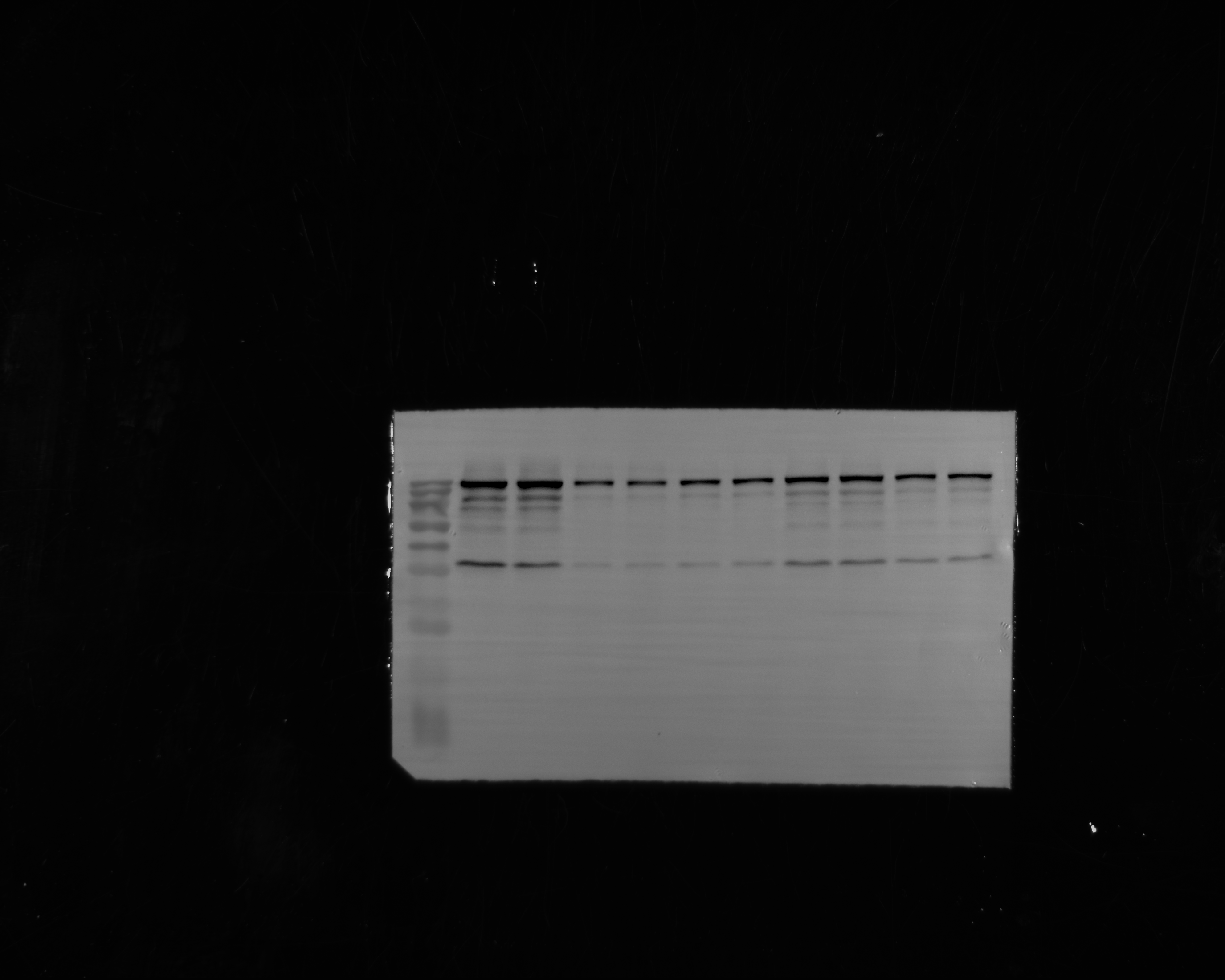

Supplement: S9 Fig — (A) Duplicate expression images for each target protein band. (B)Single expression image for each target protein band. (ZIP) [file pone.0310897.s009.zip › S9 Fig/A/Collagen II.tif]

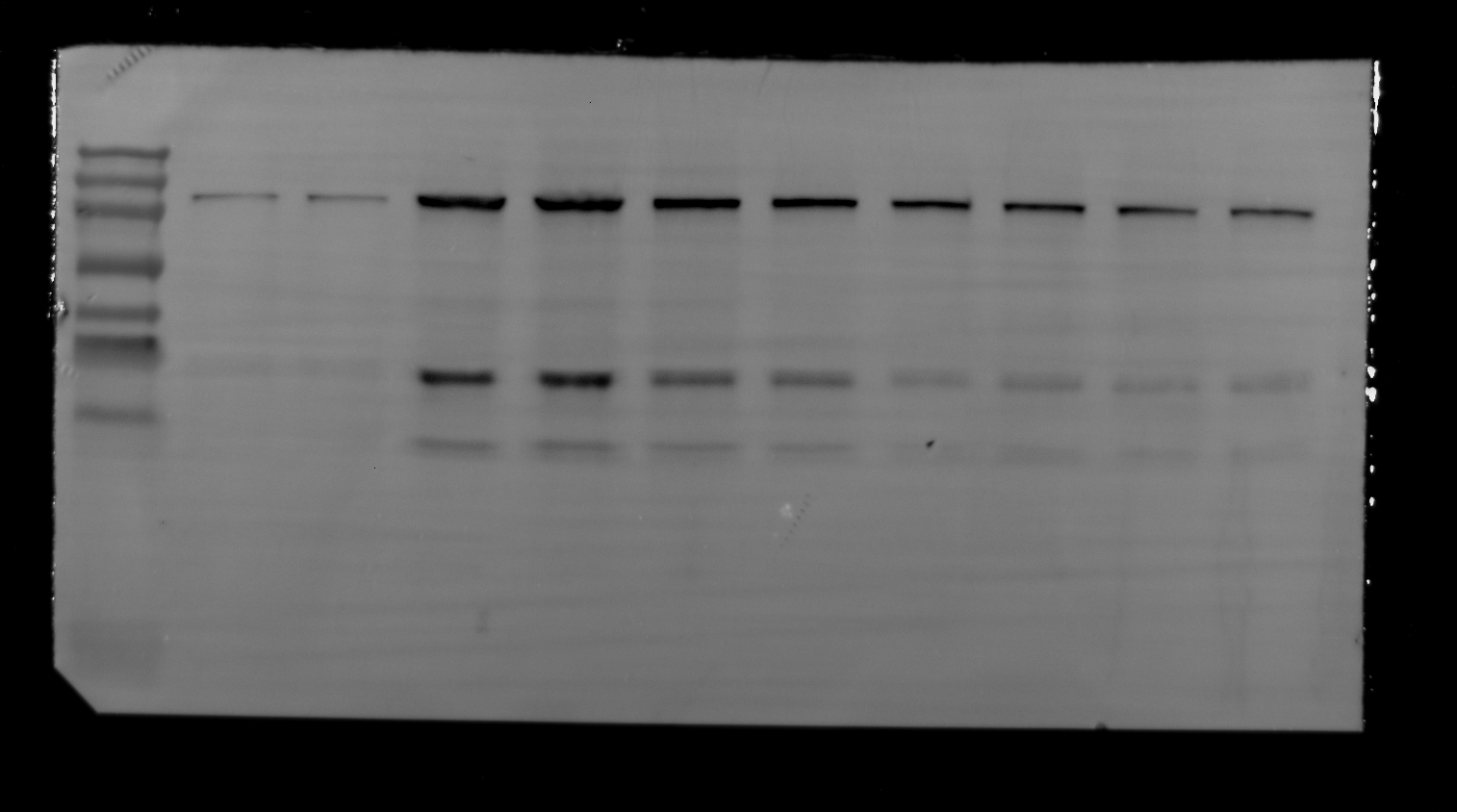

Supplement: S9 Fig — (A) Duplicate expression images for each target protein band. (B)Single expression image for each target protein band. (ZIP) [file pone.0310897.s009.zip › S9 Fig/A/Collagen III.tif]

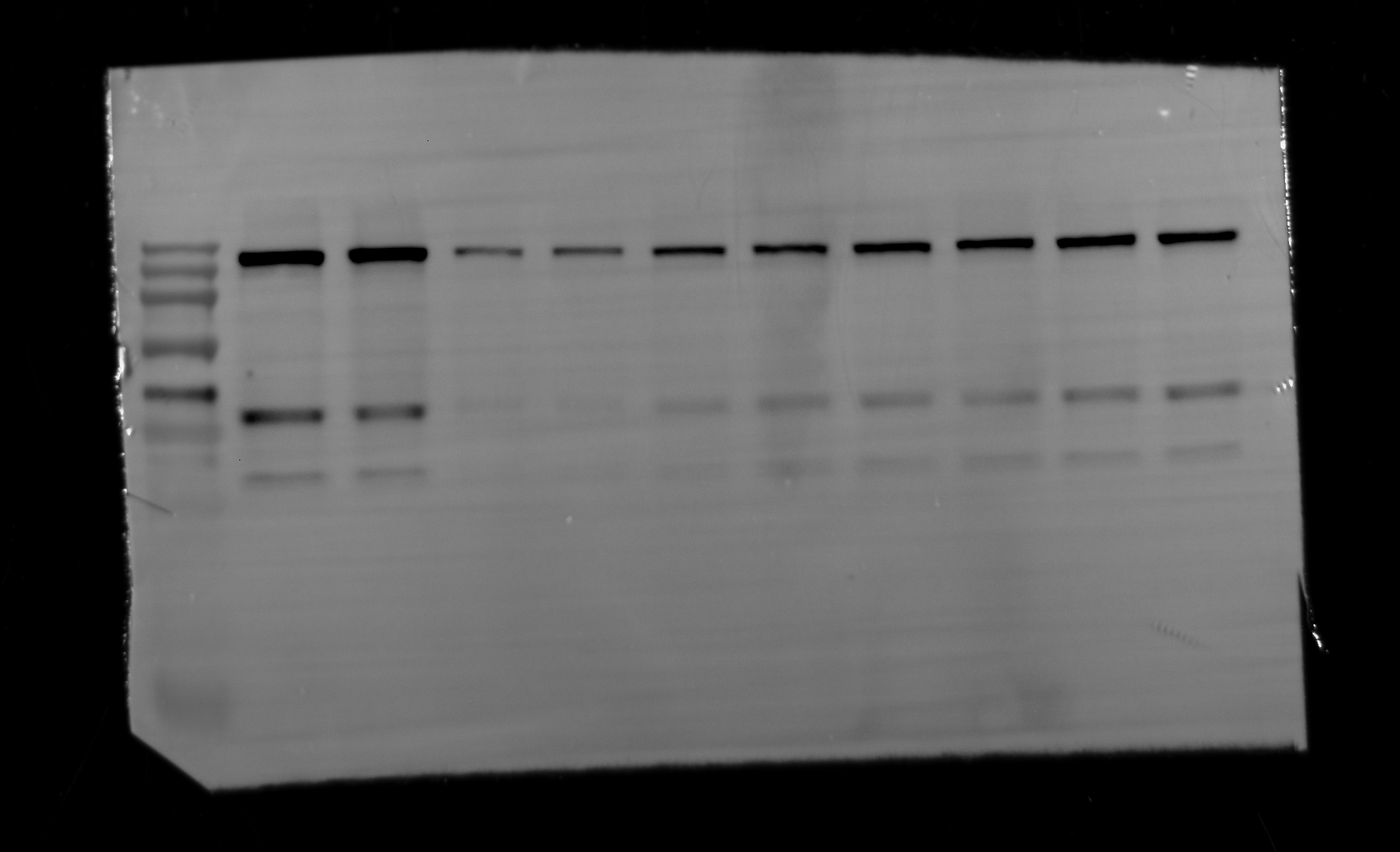

Supplement: S9 Fig — (A) Duplicate expression images for each target protein band. (B)Single expression image for each target protein band. (ZIP) [file pone.0310897.s009.zip › S9 Fig/A/Collagen IV.tif]

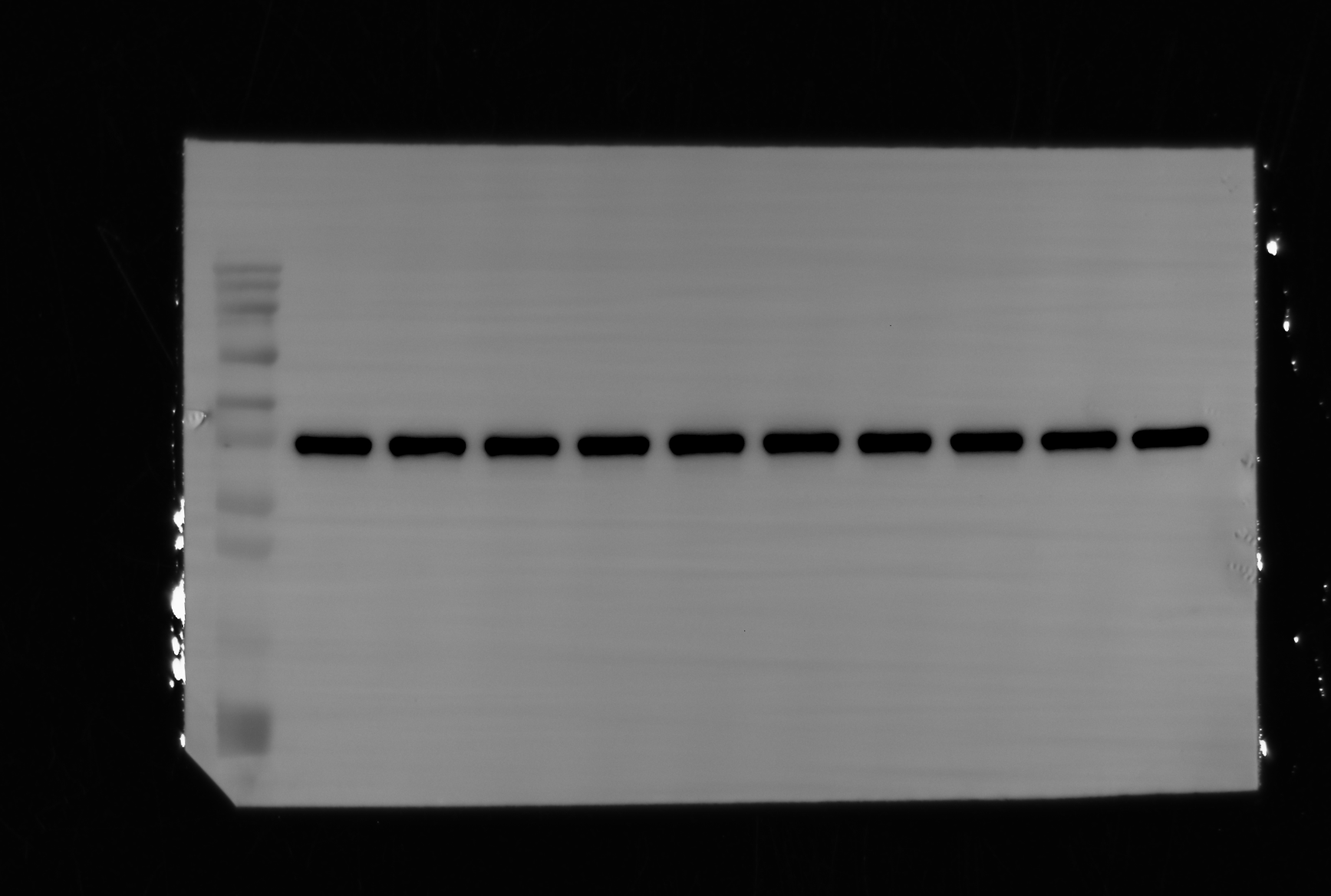

Supplement: S9 Fig — (A) Duplicate expression images for each target protein band. (B)Single expression image for each target protein band. (ZIP) [file pone.0310897.s009.zip › S9 Fig/A/GAPDH.tif]

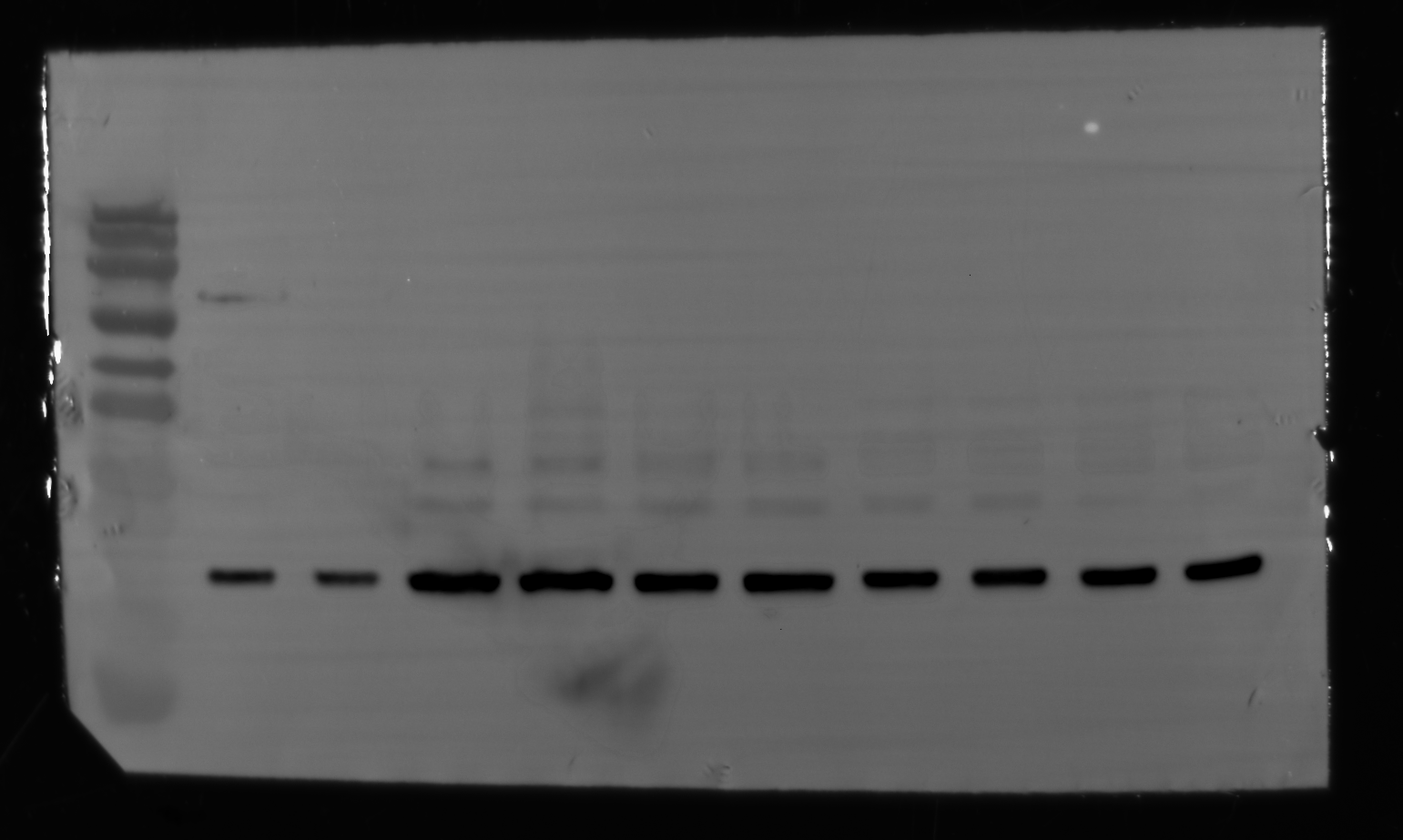

Supplement: S9 Fig — (A) Duplicate expression images for each target protein band. (B)Single expression image for each target protein band. (ZIP) [file pone.0310897.s009.zip › S9 Fig/A/TGF-β1.tif]

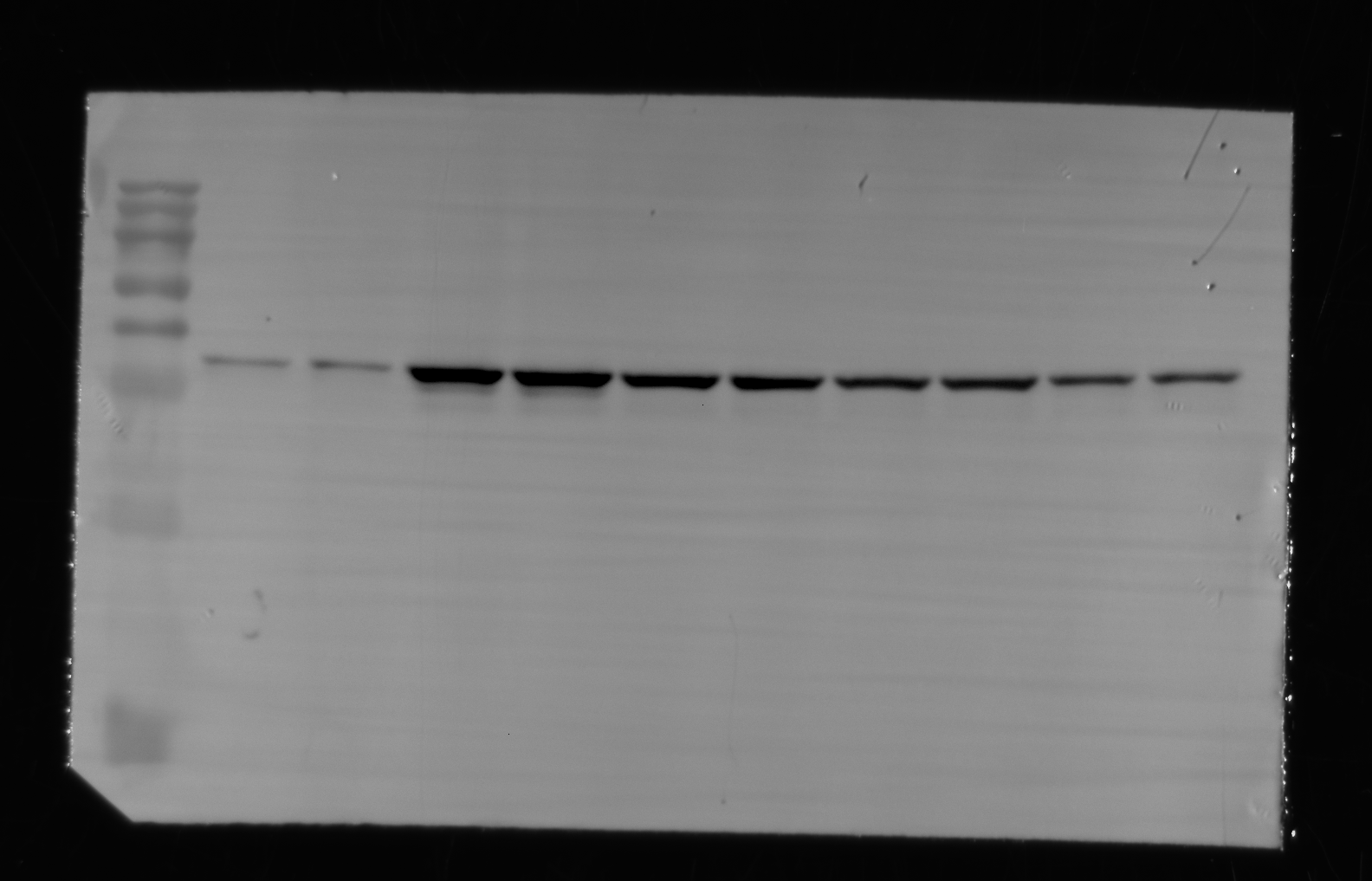

Supplement: S9 Fig — (A) Duplicate expression images for each target protein band. (B)Single expression image for each target protein band. (ZIP) [file pone.0310897.s009.zip › S9 Fig/A/α-SMA.tif]

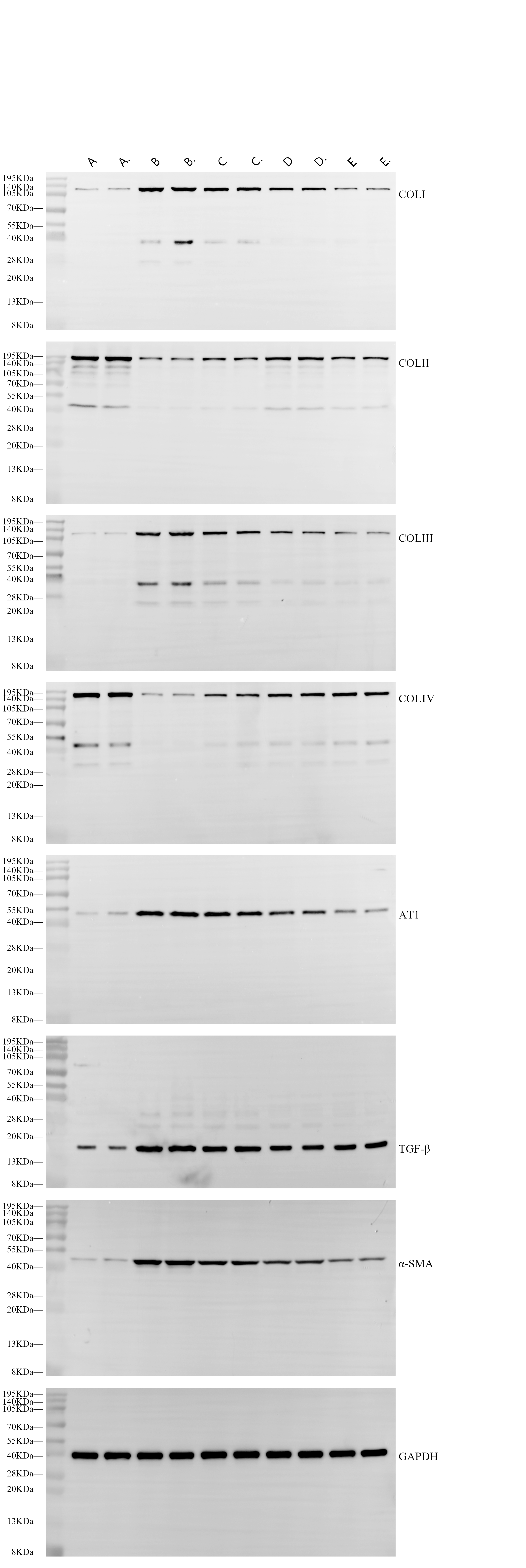

Supplement: S9 Fig — (A) Duplicate expression images for each target protein band. (B)Single expression image for each target protein band. (ZIP) [file pone.0310897.s009.zip › S9 Fig/A/整理图片1.tif]

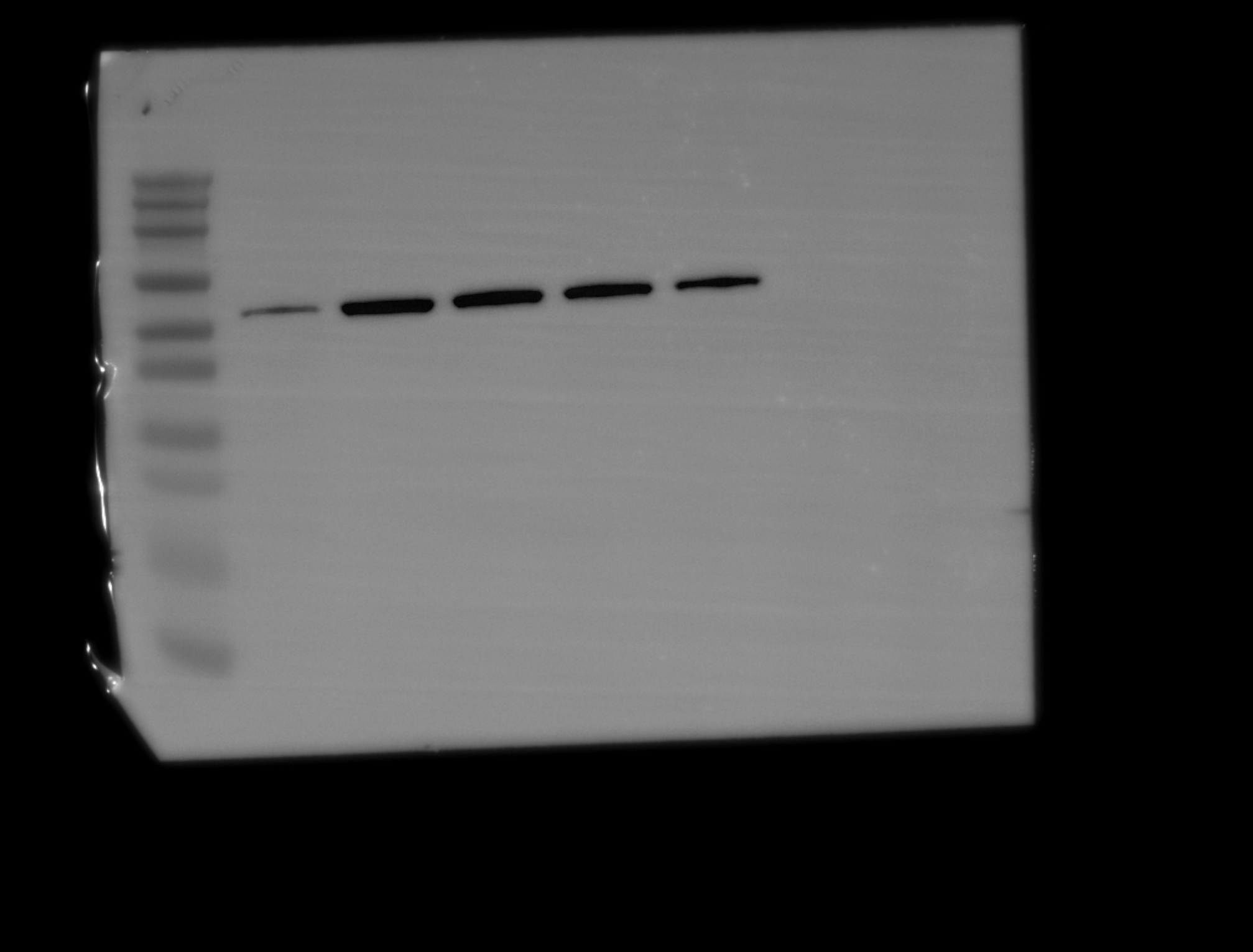

Supplement: S9 Fig — (A) Duplicate expression images for each target protein band. (B)Single expression image for each target protein band. (ZIP) [file pone.0310897.s009.zip › S9 Fig/B/AT1.tiff]

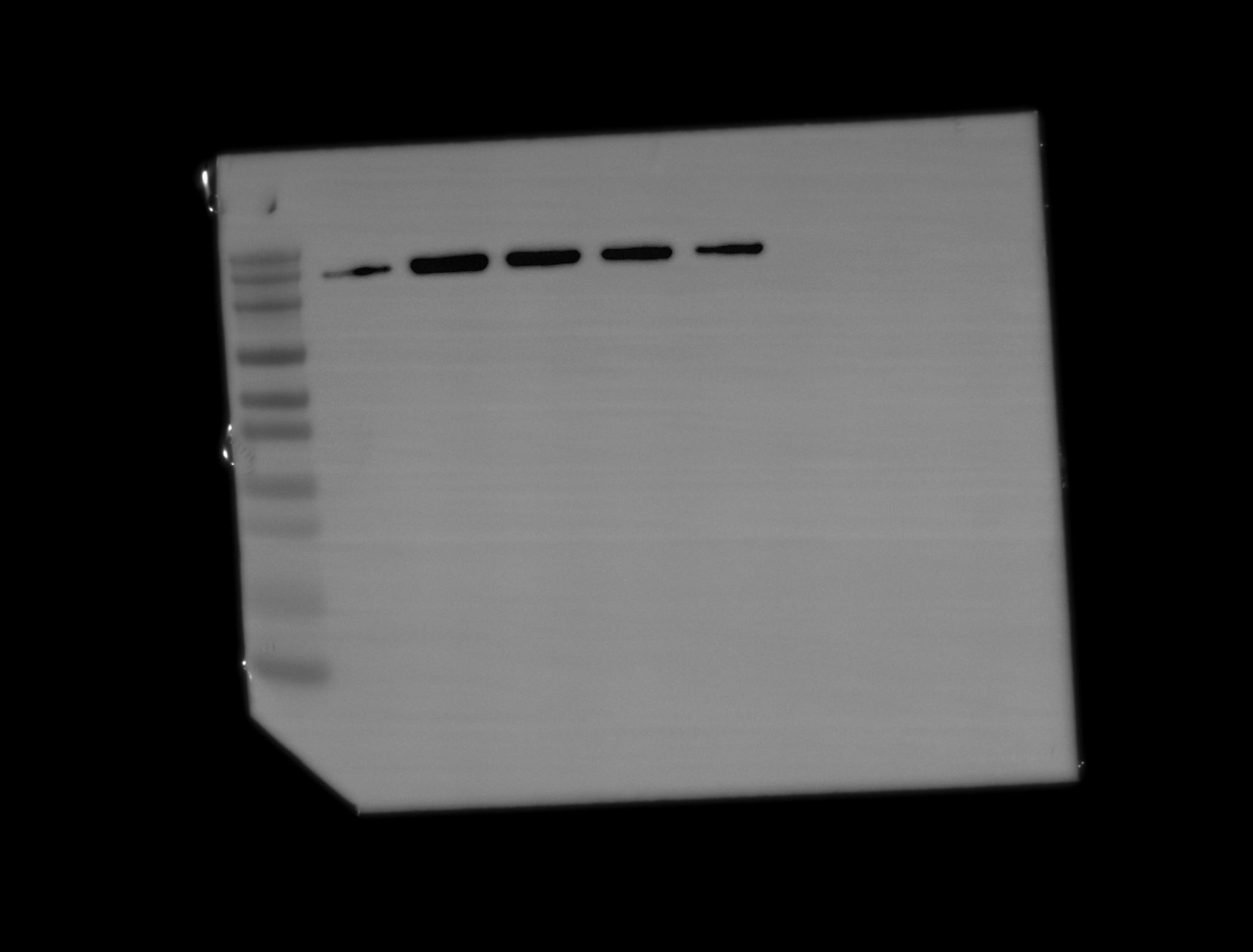

Supplement: S9 Fig — (A) Duplicate expression images for each target protein band. (B)Single expression image for each target protein band. (ZIP) [file pone.0310897.s009.zip › S9 Fig/B/Collagen I.tiff]

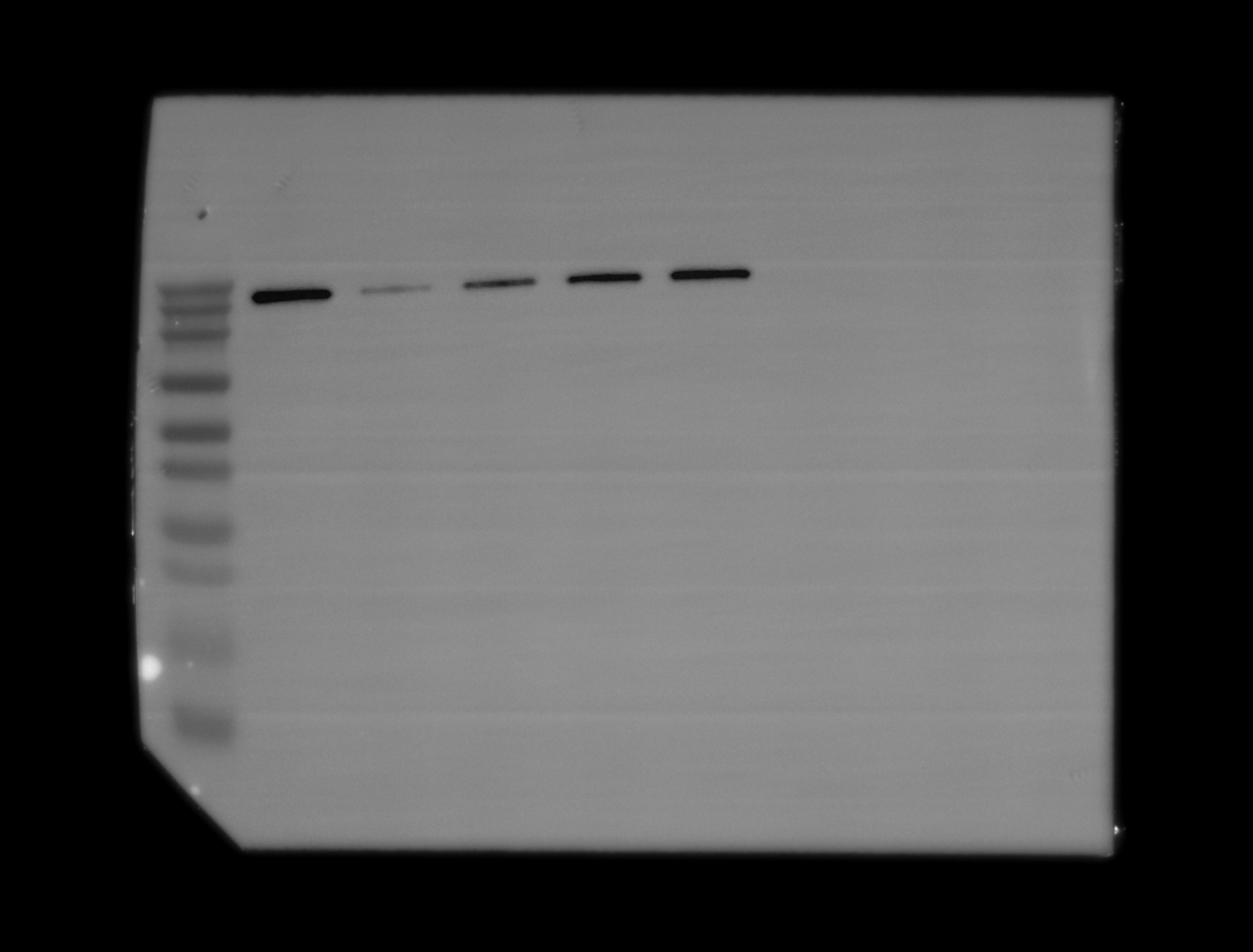

Supplement: S9 Fig — (A) Duplicate expression images for each target protein band. (B)Single expression image for each target protein band. (ZIP) [file pone.0310897.s009.zip › S9 Fig/B/Collagen IV.tiff]

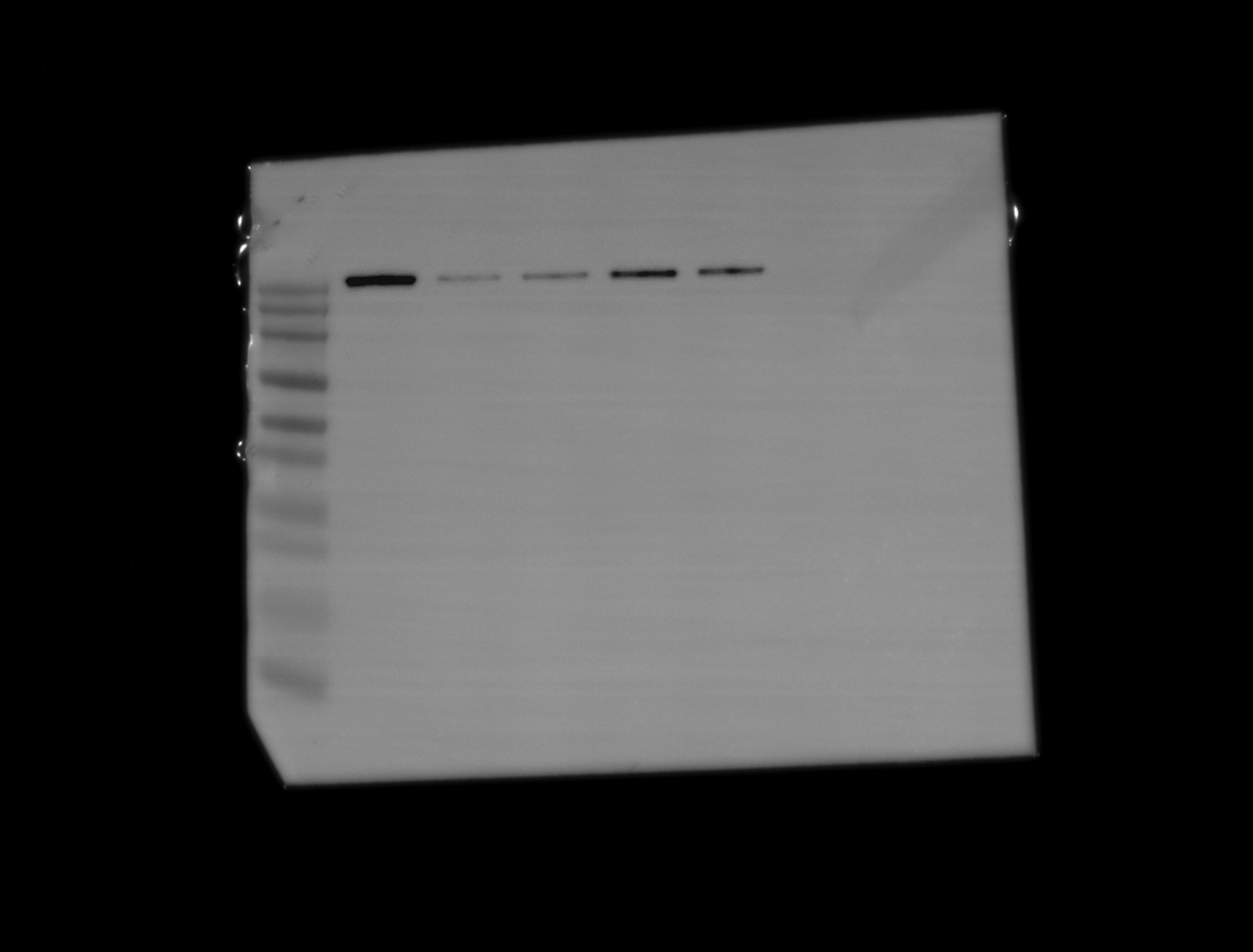

Supplement: S9 Fig — (A) Duplicate expression images for each target protein band. (B)Single expression image for each target protein band. (ZIP) [file pone.0310897.s009.zip › S9 Fig/B/Collagen II.tiff]

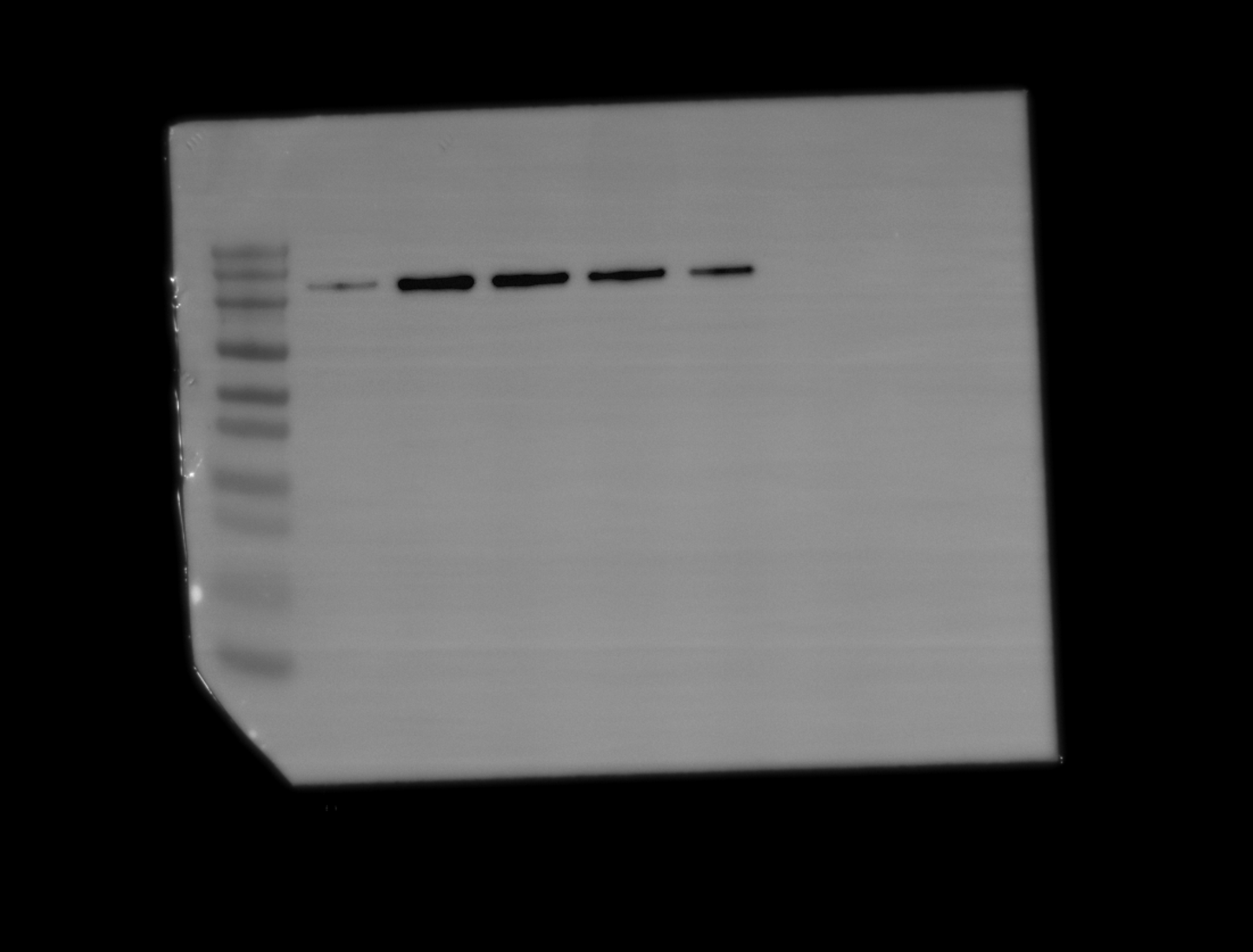

Supplement: S9 Fig — (A) Duplicate expression images for each target protein band. (B)Single expression image for each target protein band. (ZIP) [file pone.0310897.s009.zip › S9 Fig/B/Collagen III.tiff]

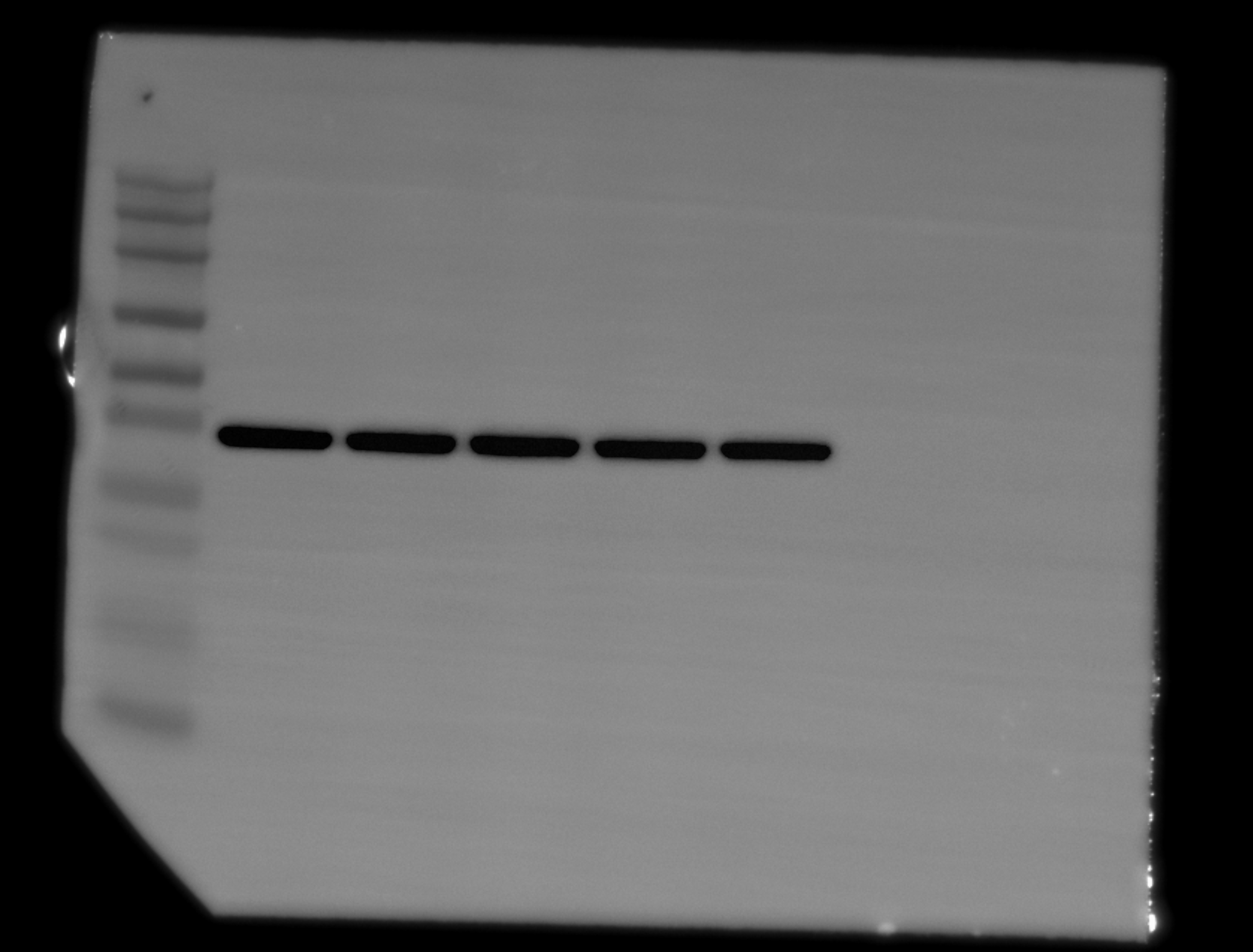

Supplement: S9 Fig — (A) Duplicate expression images for each target protein band. (B)Single expression image for each target protein band. (ZIP) [file pone.0310897.s009.zip › S9 Fig/B/GAPDH.tiff]

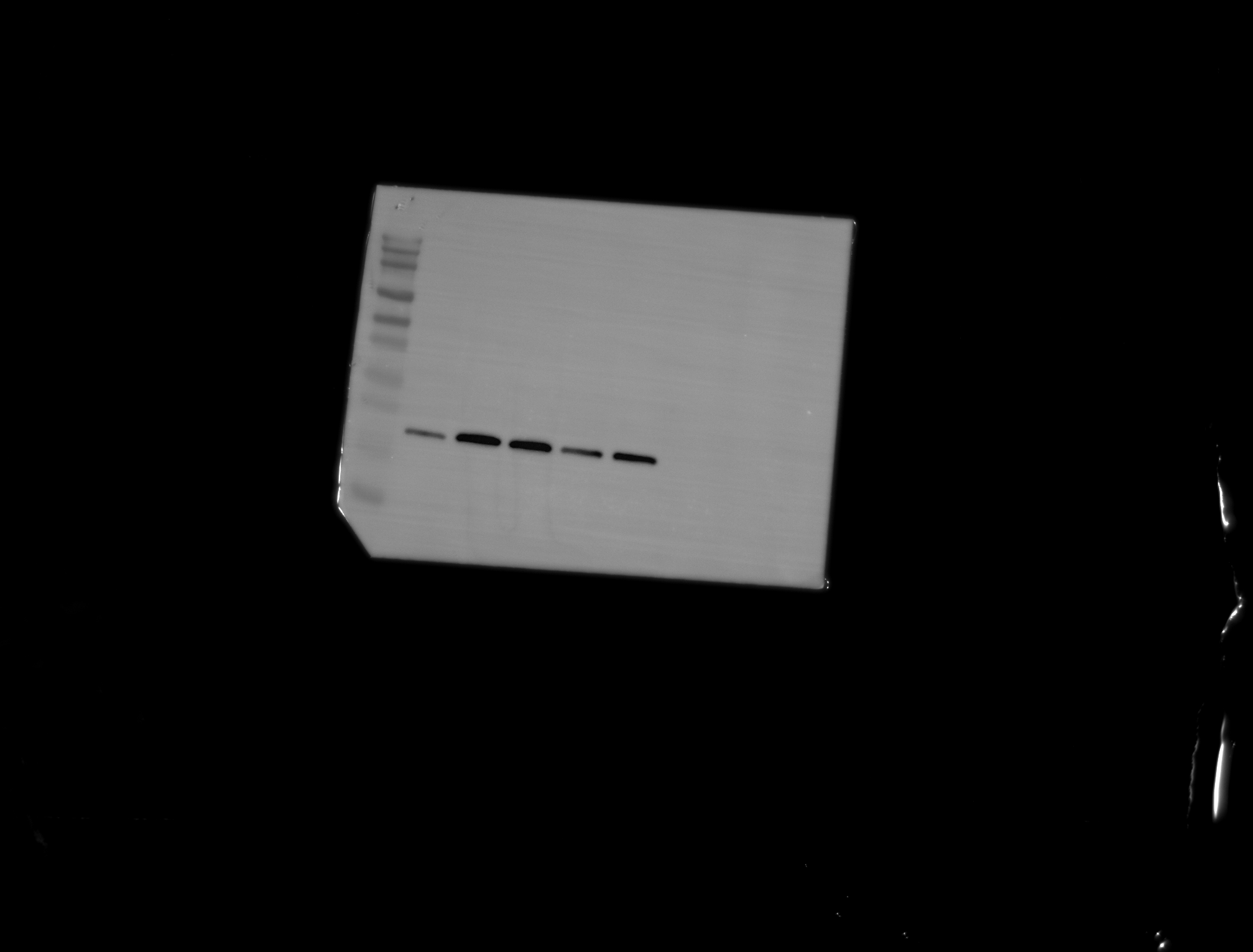

Supplement: S9 Fig — (A) Duplicate expression images for each target protein band. (B)Single expression image for each target protein band. (ZIP) [file pone.0310897.s009.zip › S9 Fig/B/TGF-β1.tiff]

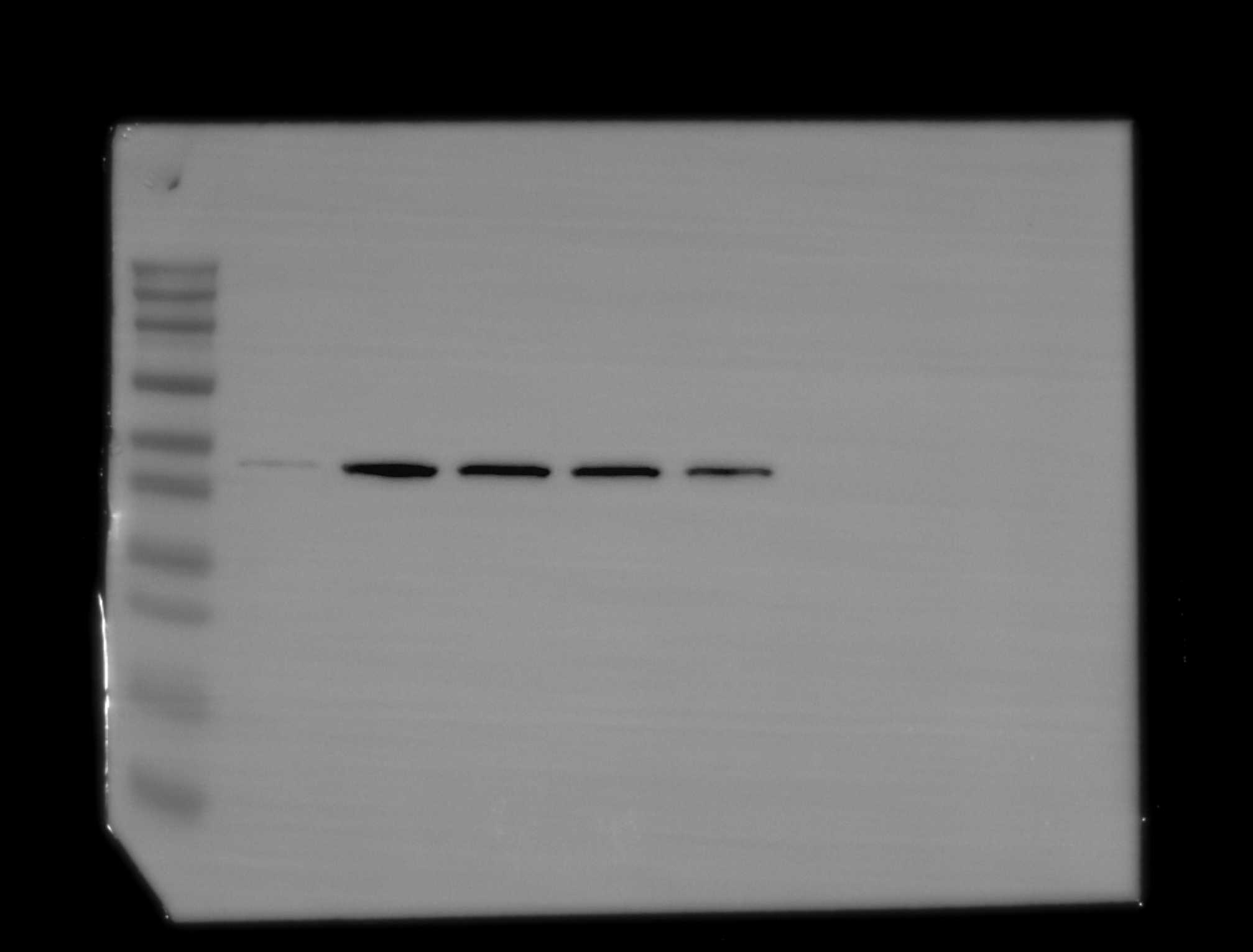

Supplement: S9 Fig — (A) Duplicate expression images for each target protein band. (B)Single expression image for each target protein band. (ZIP) [file pone.0310897.s009.zip › S9 Fig/B/α-SMA.tiff]
