# Supplementary material for: Qili Qiangxin capsule attenuates myocardial fibrosis by modulating collagen homeostasis post-infarction in rats
Source: PLoS One. 2024 Sep 27;19(9):e0310897. doi: 10.1371/journal.pone.0310897 (PMC11432860; doi:10.1371/journal.pone.0310897)

各组大鼠Ⅱ导联心电图

假手术组　　体重 259.7　解剖前体重 267.5

开胸前造模前心电图

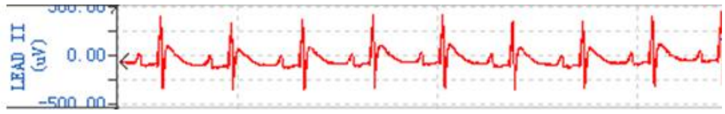

闭胸后造模后心电图

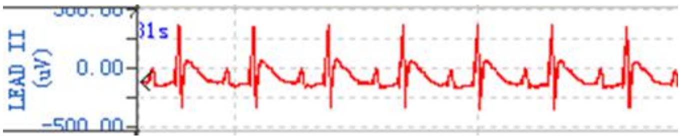

取材解剖前心电图

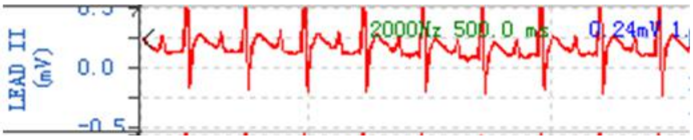

模型组　术前体重 264.4　解剖前体重 271.5

开胸前造模前心电图

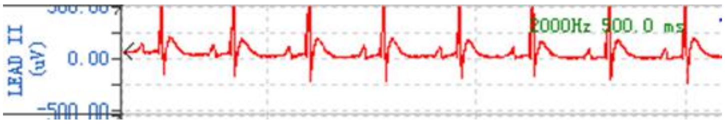

闭胸后造模后心电图

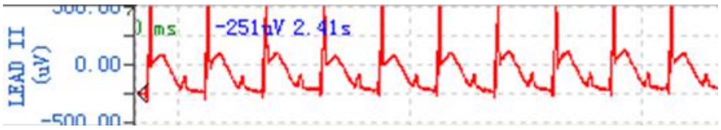

取材解剖前心电图

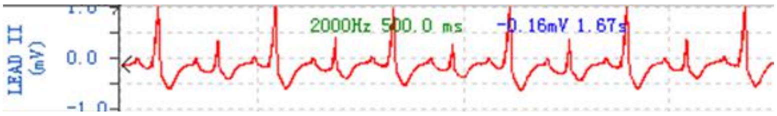

芪苈低剂量组　术前体重 221.6　解剖前体重 258.7

开胸前造模前心电图

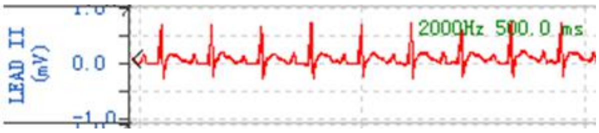

闭胸后造模后心电图

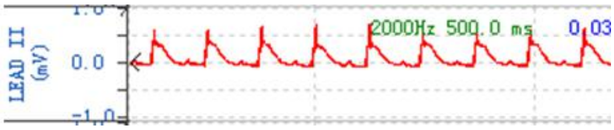

取材解剖前心电图

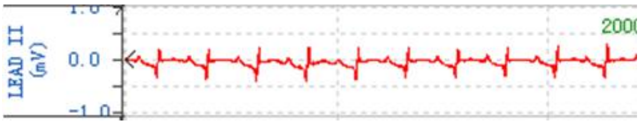

芪苈高剂量组 术前体重 260.0g　解剖前体重 284.4g

开胸前造模前心电图

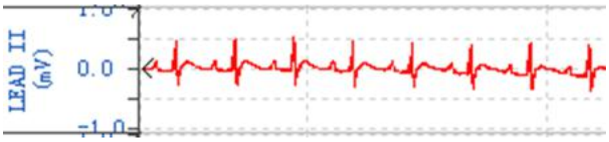

闭胸后造模后心电图

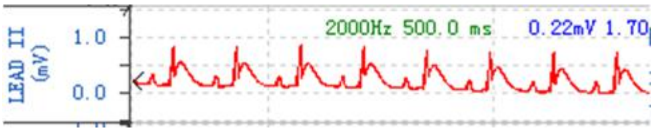

取材解剖前心电图

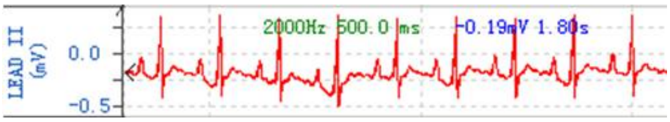

恩格列净组 术前体重 210.8　解剖前体重 250.9

开胸前造模前心电图

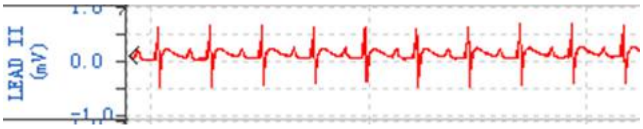

闭胸后造模后心电图

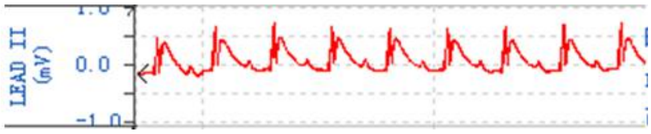

取材解剖前心电图

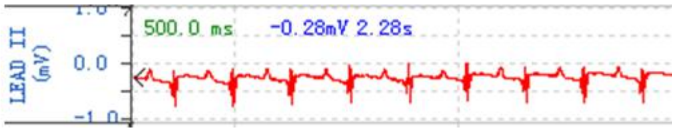

Supplement: S2 Fig — (A) Original electrocardiogram images of rats before combination for each group. (B) Original electrocardiogram images of rats in lead II. (ZIP) [file pone.0310897.s002.zip › S2 Fig/B raw images-Fig2 ECG.pdf]
