# Supplementary material for: Qili Qiangxin capsule attenuates myocardial fibrosis by modulating collagen homeostasis post-infarction in rats
Source: PLoS One. 2024 Sep 27;19(9):e0310897. doi: 10.1371/journal.pone.0310897 (PMC11432860; doi:10.1371/journal.pone.0310897)
Supplement: S10 Fig — (ZIP) [file pone.0310897.s010.zip › S10 Fig/The methods and results of qPCR experiments.pdf]

The methods and results of qPCR experiments.

Method Description: Total RNA was extracted using Trizol reagent (batch number: 15596018CN) (Invitrogen, Carlsbad, CA, USA). cDNA was synthesized by the One-Step gDNA Remover kit (batch number: G3337) purchased from Wuhan Servicebio Biotechnology Co., Ltd. (Wuhan, China). The mRNA level was analyzed by RT-PCR using primers synthesized from Beijing Tsingke Biotech Co., Ltd. (Beijing, China). The relative expression level of AT1R mRNA was normalized to GAPDH mRNA. Data were analyzed by the $2^{-\triangle\triangle CT}$ method. The primer for GAPDH was Forward primer: 5' CTGGAGAAACCTGCCAAGTATG3' and Reverse primer: 5'GGTGGAAGAATGGGAGTTGCT3'. The primer for AT1R was Forward primer: 5' CTCTGCCACATTCCCTGAGTTA3' and Reverse primer: 5' TGGGGCAGTCATCTTGGATTC3'.

Results Analysis: The results are presented in the Supporting Information files and the figure below (Figure 7A), which demonstrates that the gene expression of AT1R is consistent with the protein expression observed in our WB analysis. The RT-qPCR melting amplification curves for the housekeeping gene GAPDH and the target gene AT1R are illustrated in Figure 7B-E. The primers were used at appropriate concentrations, with no indication of genomic DNA contamination or nonspecific amplification, confirming the specificity of the reaction. The melting and amplification curves for both the GAPDH and AT1R indicate a stable amplification system, and the melting curves exhibit a clear single-peak pattern.

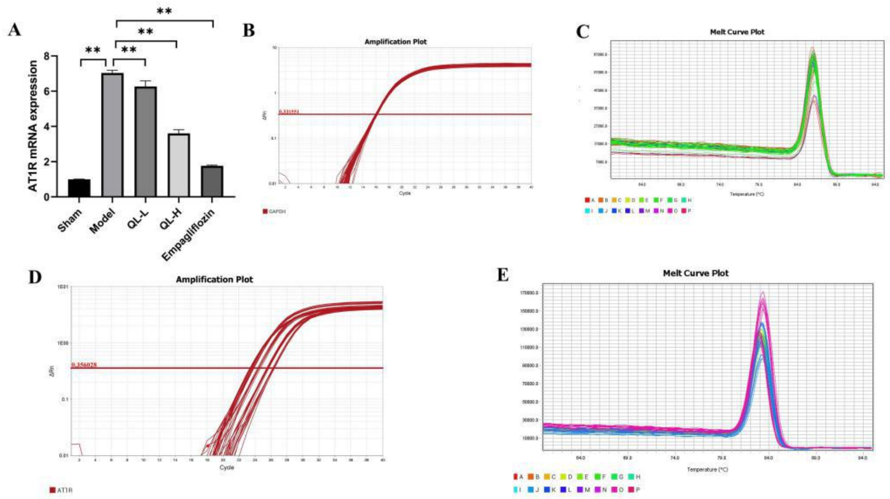

Figure 7. QLQX changed the expression of AT1R mRNA in RT-qPCR.

A, Expression of AT1R mRNA in RT-qPCR in each group. ** P<0.01. n=3 per group. Data were shown as the mean ± SD, and all by analysis of variance. **B,** The RT-qPCR amplification curve for the GAPDH. **C,** The RT-qPCR melting curve for the GAPDH. **D,** The RT-qPCR amplification curve for the AT1R. **E,** The RT-qPCR melting curve for the AT1R.
