# Supplementary material for: Qili Qiangxin capsule attenuates myocardial fibrosis by modulating collagen homeostasis post-infarction in rats
Source: PLoS One. 2024 Sep 27;19(9):e0310897. doi: 10.1371/journal.pone.0310897 (PMC11432860; doi:10.1371/journal.pone.0310897)
Supplement: S1 Table — (DOCX) [file pone.0310897.s012.docx]

Table 1

The components of Qili Qiangxin capsule.

| Scientific name | Local name | Family | Amount |
| --- | --- | --- | --- |
| *Astragalus membranaceus (Fisch.) Bge. var. mongholicus (Bge.) Hsiao* | Huangqi | *Fabaceae* | 450g |
| *Panax ginseng C. A. Mey* | Renshen | *Araliaceae* | 225g |
| *Salvia miltiorrhiza Bunge* | Danshen | *Lamiaceae* | 225g |
| *Alisma orientale (Sam.) Juzep* | Zexie | *Alismataceae* | 225g |
| *Rhizoma Atractylodis Macrocephalae* | Xiangjiapi | *Fabaceae* | 180g |
| *Lepidium apetalum Willd* | Tinglizi | *Brassicaceae* | 150g |
| *Aconitum carmichaelii Debx* | Fuzi | *Ranunculaceae* | 112.5g |
| *Cinnamomum cassia (L.) J. Presl* | Guizhi | *Lauraceae* | 90g |
| *Carthamus tinctorius L.* | Honghua | *Asteraceae* | 90g |
| *Citrus reticulata Blanco* | Chenpi | *Rutaceae* | 75g |
| *Rhizoma Polygonati* | Yuzhu | *Asparagaceae* | 75g |
